# Specific Post-Translational Modifications of VDAC3 in ALS-SOD1 Model Cells Identified by High-Resolution Mass Spectrometry

**DOI:** 10.3390/ijms232415853

**Published:** 2022-12-13

**Authors:** Maria Gaetana Giovanna Pittalà, Simona Reina, Stefano Conti Nibali, Annamaria Cucina, Salvatore Antonio Maria Cubisino, Vincenzo Cunsolo, Giuseppe Federico Amodeo, Salvatore Foti, Vito De Pinto, Rosaria Saletti, Angela Messina

**Affiliations:** 1 Organic Mass Spectrometry Laboratory, Department of Chemical Sciences, University of Catania, Via S. Sofia 64, 95123 Catania, Italy; 2 Department of Biomedical and Biotechnological Sciences, University of Catania, Via S. Sofia 64, 95123 Catania, Italy; 3 Department of Neurology, Columbia University, New York, NY 10032, USA; 4 Department of Biological, Geological and Environmental Sciences, University of Catania, Via S. Sofia 64, 95123 Catania, Italy

**Keywords:** voltage dependent anion channel, post-translational modifications, over-oxidation, deamidation, succination, amyotrophic lateral sclerosis, SOD1, mitochondria, neurodegeneration, high-resolution mass spectrometry

## Abstract

Damage induced by oxidative stress is a key driver of the selective motor neuron death in amyotrophic lateral sclerosis (ALS). Mitochondria are among the main producers of ROS, but they also suffer particularly from their harmful effects. Voltage-dependent anion-selective channels (VDACs) are the most represented proteins of the outer mitochondrial membrane where they form pores controlling the permeation of metabolites responsible for mitochondrial functions. For these reasons, VDACs contribute to mitochondrial quality control and the entire energy metabolism of the cell. In this work we assessed in an ALS cell model whether disease-related oxidative stress induces post-translational modifications (PTMs) in VDAC3, a member of the VDAC family of outer mitochondrial membrane channel proteins, known for its role in redox signaling. At this end, protein samples enriched in VDACs were prepared from mitochondria of an ALS model cell line, NSC34 expressing human SOD1G93A, and analyzed by nUHPLC/High-Resolution nESI-MS/MS. Specific over-oxidation, deamidation, succination events were found in VDAC3 from ALS-related NSC34-SOD1G93A but not in non-ALS cell lines. Additionally, we report evidence that some PTMs may affect VDAC3 functionality. In particular, deamidation of Asn215 alone alters single channel behavior in artificial membranes. Overall, our results suggest modifications of VDAC3 that can impact its protective role against ROS, which is particularly important in the ALS context. Data are available via ProteomeXchange with identifier PXD036728.

## 1. Introduction

Amyotrophic lateral sclerosis (ALS) is an adult neurodegenerative disease characterized by the progressive loss of upper and lower motor neurons in the spinal cord and brainstem [1]. From its onset, the disease progresses rapidly, leading the patient to death [2]. To date, there are no effective treatments to counteract the outcome of the disease.

About 10% of ALS cases are associated with genetic defects (familial ALS, fALS), while the most common forms are sporadic (sALS, about 90–95% of cases). Although various environmental factors have been indicated as being responsible for sALS, the importance of the genetic component has also been demonstrated for these more common forms of the disease [3].

The Cu/Zn superoxide dismutase (SOD1) gene is responsible for about 20% of fALS cases [4] and SOD1 misfolded wild-type (SOD1WT) toxicity is involved in the pathogenesis of sALS where the toxic gain of function is due to redox disturbance that changes the biophysical properties of native SOD1 protein [5].

The exact mechanism of neurotoxicity associated with SOD1 mutants remains unknown, although several hypotheses have been proposed to elucidate the toxic effects dependent on mutated SOD1 and the subsequent neurodegeneration observed in ALS. These include mitochondrial dysfunction, oxidative stress, excitotoxicity caused by aberrant glutamate signaling, endoplasmic reticulum stress, and protein aggregation [6].

In particular, in NSC34 cells expressing the human SOD1 G93A ALS-associated mutant, oxidative stress was greater than in normal NSC34 cells or those transfected with wild-type human SOD1 [7,8]. In addition, the affected tissues of ALS patients are characterized by extensive mitochondrial dysfunction and oxidative damage to proteins, lipids, and nucleic acids as a result of an imbalance between free radical production and degradation [6,9]. It has been shown that, in the spinal cord of ALS patients, VDAC1, the most abundant of the mitochondrial porins called Voltage-Dependent Anion selective Channels (VDAC), is the docking site on the surface of mitochondria for SOD1-G93A mutants [10]. The interaction of VDAC1 with mutant SOD1 hinders the binding of cytosolic hexokinase I (HKI) and probably other physiological interactors, causing several dramatic consequences for mitochondrial function [11]. VDACs are relevant components of the outer mitochondrial membrane (OMM) whose main role is to form water-filled pores that allow the exchange of metabolites and molecules to and from the mitochondrion. In mammals, three genes encode three highly conserved isoforms named VDAC1, VDAC2 and VDAC3, agreeing to the order of their discovery. Under physiological conditions, VDACs play numerous roles in cellular metabolism and mitochondrial function [12] by interacting with important molecules involved in survival pathways. On the other hand, defective VDAC channels are associated with a myriad of cellular disease states such as cancer, diabetes, viral infections, and neurodegenerative diseases such as ALS but also Alzheimer’s disease and Parkinson’s disease [13,14].

In general, these diseases are not directly related to a single defective VDAC isoform, but mitochondria in the tissues of affected patients show morphological and biochemical abnormalities with alterations in protein–protein interactions [15].

Post-translational modifications (PTMs) are implicated in many cellular events as they modulate and alter the range of possible functions of proteins as well as make changes to their structure [16]. The PTMs of a protein can, thus, affect its localization, turnover, activity status, and interactions with other proteins [17].

The full set of PTMs that regulate the interactions of VDACs with other cytosolic and mitochondrial proteins is still an understudied field [18]. In recent years, the development of nano-reverse phase ultra-high performance liquid chromatography (nanoRP-UHPLC) and ultrasensitive high resolution mass spectrometry (HRMS) methods has expanded knowledge in this field [19]. However, there are still few studies on PTMs of VDAC proteins in pathological conditions such as neurodegeneration [19].

Recently, using mass spectrometry-based methods, we demonstrated for the first time that VDAC1 from a SOD1-ALS model cell line undergoes specific deamidation of asparagine and glutamine residues [20]. Bioinformatic analysis revealed increased channel instability in deamidated VDAC1, which is responsible for a broad conformational change that may alter the physiological pool of interactors and increase the binding of ALS-related mutant forms of SOD1 [20].

Although, the role of VDAC1 in neurodegeneration is well known [12,21,22], the involvement of the other two isoforms in these pathways remains poorly defined.

This is probably due to the greater relative abundance of VDAC1 compared with the other isoforms. In particular, although VDAC3 is the least abundant and least studied isoform, important functional roles, as an oxidative stress sensor or tumor suppressor, have been proposed for this isoform in cellular and animal models [16,23].

In this work, by combining HRMS analysis with “*in-solution*” digestion of an enriched fraction of VDACs, in VDAC3 purified from NSC34-SOD1G93A model ALS-SOD1 cells, we identified specific irreversible PTMs that can destabilize the channel by impacting its function.

## 2. Results

This work follows our previous study on NSC34 model SOD1-ALS cells. In that work, we had identified specific post-translational modifications in VDAC1 that can lead to important changes in the channel structure and, thus, in the bioenergetic metabolism of ALS motor neurons [20]. Considering that changes found are most likely consequential to the high levels of oxidative stress typical of motor neurons affected by ALS, in this work we wanted to extend our analysis also to the VDAC3 isoform purified from ALS-related NSC34-SOD1G93A cells and, as a control, from NSC34-SOD1WT or NSC34 cells. Recently, we established the important role played by VDAC3, but not VDAC1, in the cellular response to ROS-induced oxidative stress, showing in particular that VDAC3 cysteines are essential for the ability of the protein to control mitochondrial ROS homeostasis [24]. In particular, by using HRMS analysis we searched oxidized or succinate cysteines, oxidized or dioxidized methionines, ubiquitinated lysines, phosphorylated serines/threonines/tyrosines, citrullinated arginines, cysteinylated and deamidated asparagines and glutamines. Protein reduction and alkylation were performed before the purification of VDACs from mitochondria, to rule out the possibility of non-specific and/or unwanted oxidation occurring during the purification protocol. Hydroxyapatite (HTP) eluates were digested in-solution using trypsin and chymotrypsin, and then the highly complex enzyme peptide mixtures were analyzed in triplicate (technical replicates) by liquid chromatography–high resolution mass spectrometry.

**Figure 1:**
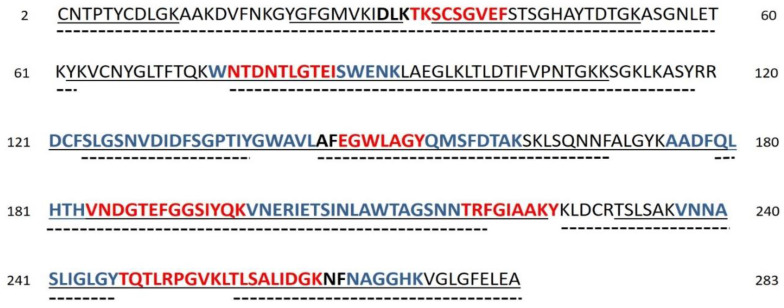
Sequence coverage map of VDAC3 from NSC34, NSC34-SOD1WT and NSC34-SOD1G93A cell lines obtained by tryptic and chymotryptic digestion. The solid line indicates the sequence that was obtained from tryptic peptides; dotted lines: sequence obtained from chymotryptic peptides. Unique tryptic (indicated in bold and blue) and chymotryptic (indicated in bold and black) peptides originating from missed cleavages were used to distinguish and cover the sequences shared by isoforms. Sequences shared by multiple isoforms are shown in red. Sequence numbering considered the starting methionine residue, which is eliminated during protein maturation.

The Mus musculus VDAC3 sequence (SwissProt Acc. N. Q60931) includes two methionines at positions 26 and 155, and six cysteines at positions 2, 8, 36, 65, 122, and 229. The numbering adopted here starts from Met^1^, which is actually absent in the mature protein as confirmed by our MS data (Figure 1) and similar to rat and human VDAC3 isoforms [25,26].

The results obtained from tryptic and chymotryptic fragments analyses yielded 98.9% protein coverage (279 of 282 amino acid residues) (Figure 1), with the exception of the dipeptide Arg119-Arg120 and Tyr225. Although some tryptic peptides are shared among the three VDAC isoforms, due to the identification of unique peptides originating from missed cleavages, the obtained sequence coverage unambiguously identifies the mouse VDAC3 protein (Figure 1). MS analysis performed on samples of VDAC3 prepared by the same procedure but purified from another set of cell cultures of NSC34, NSC34-SOD1WT, and NSC34-SOD1G93A (biological replicates) confirmed the results obtained and the reproducibility of the experimental data produced (data not shown).

### 2.1. Mass Spectrometry Analysis of VDAC3 from NSC34 Cell Line

In the nanoRP-UHPLC/High-Resolution ESI-MS/MS analysis of the enzyme digests, Met^26^ and Met^155^ were identified in the normal form (Table S1, fragment 2, and Table S2, fragments 4, 21), and also in the sulfoxide form (Table S3, fragments 1, 5, 6, and Table S4, fragments 1, 2). Figure S1A,B shows the full scan and fragment ion mass spectra of the doubly charged molecular ion of the peptide GYGFGMVK with Met^26^ modified as methionine sulfoxide (Figure S1A), and the triply charged molecular ion of the peptide D*C*FSLGSNVDIDFSGPTIYGWAVLAFEGWLAGYQMSFDTAK with Met^155^ as methionine sulfoxide (Figure S1B). This oxidation was confirmed by the presence in the MS/MS spectra of the characteristic neutral loss of 64 Da corresponding to the ejection of methanesulfenic acid from the side chain of MetO [27].

Although from these data it was not possible to obtain a precise determination of the relative amount of Met and Met sulfoxide, by comparing the absolute intensities of the multiply charged molecular ions of the respective peptides, a rough estimate of their relative abundance could be achieved. These calculations indicated a ratio of about 10:1 and 0.5:1 MetO/Met for Met^26^ (Table 1, Table 2, Tables S5 and S6) and Met^155^ (Table 2 and Table S6), respectively.

Furthermore, partial oxidation of Cys^36^, Cys^65^, and Cys^229^ to sulfonic acid (Table S3, fragments 2–4, and Table S4, fragment 3) was revealed (Figure S2A–C). Again, from the absolute intensities of the multiply charged molecular ions of the tryptic peptides with Cys oxidized to sulfonic acid compared with the same fragments with Cys in carboxyamidomethylated form, an Ox/Red ratio of about 0.08, 0.9, and 0.1 was observed for Cys^36^, Cys^65^, and Cys^229^, respectively (Table 1 and Table 2). As for the other cysteine residues, Cys 2, 8, and 122 were detected exclusively in the carboxyamidomethylated form (Table S1, fragment 1; Table S2, fragments 1–3; Table S3, fragment 5), with Cys^2^ totally acetylated (Table S1, fragment 1, and Table S2, fragments 1, 2) (Figure S3).

### 2.2. Mass Spectrometry Analysis of VDAC3 from NSC34-SOD1WT Cell Line

The PTMs identified on purified VDAC3 from NSC34-SOD1WT control cells, which express the wt form of human SOD1, are similar to those of VDAC3 from parental NSC34 control cells and similar to what has been shown previously for the VDAC1 isoform [20]. Specifically, in addition to the peptides containing methionines 26 and 155 in normal form (Table S7, fragments 2, 11, and Table S8, fragments 6, 7), peptides with these residues oxidized to methionine sulfoxide (Table S9, fragment 5, and Table S10, fragments 1, 2, and Figure S4A,B) were also detected. The MetO/Met ratio was approximately 12:1 for Met^26^ (Table 2 and Table S12) and 7:1 for Met^155^ (Table 1 and Table S11), respectively.

In addition, the partial oxidation of Cys^36^ (Table S9, fragment 2), Cys^65^ (Table S9, fragments 3 and 4) and Cys^229^ (Table S10, fragment 3) to sulfonic acid was determined (Figure S5A–C). The Ox/Red ratios of cysteines 36, 65 and 229 were 0.05, 0.6 and 0.5, respectively (Table 1, Table 2, Tables S11 and S12). In contrast, cysteines 8 and 122 were exclusively in the carboxyamidomethylated form (Table S7, fragments 1, 11 and Table S8, fragments 1–4).

**Figure 2:**
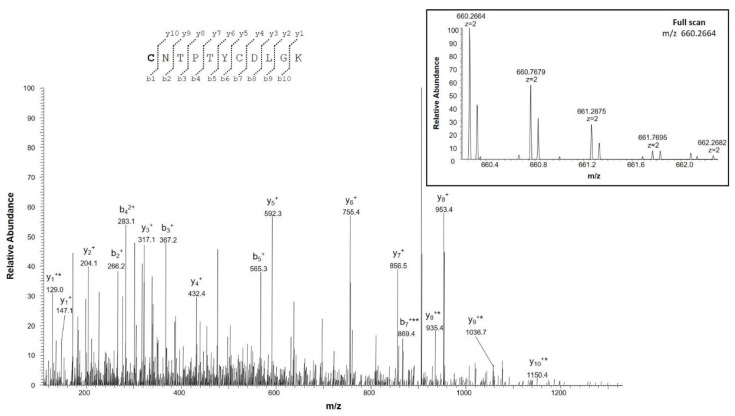
MS/MS spectrum of the doubly charged molecular ion at *m/z* 660.2664 (calculated 660.2663) of the N-terminal tryptic peptide of VDAC3 from NSC34-SOD1WT cell line with cysteine residue 2 in the form of sulfonic acid and cysteine residue 8 in the carboxyamidomethylated form. The inset shows the full scan mass spectrum of the molecular ion. Fragment ions originating from the neutral loss of H_2_O are indicated by an asterisk. The fragment ion originating from the neutral loss of NH_3_ is indicated by two asterisks.

Furthermore, unlike in NSC34 cells, we identified Cys^2^ in both reduced and acetylated form (Table S7, fragment 1, and Table S8, fragments 1–3) (Figure S6), and in both non-acetylated and oxidized to sulfonic acid (Table S9, fragment 1, and Figure 2).

### 2.3. Mass Spectrometry Analysis of VDAC3 from NSC34-SOD1G93A Cell Line

The same HRMS investigative approach was used to analyze PTMs of VDAC3 extracted from the ALS model NSC34-SOD1G93A cells, stably expressing the human SOD1G93A mutant. We identified Met^26^ and Met^155^ in the fully oxidized sulfoxide form (Table 1 and Table 2, Table S13, fragments 2, 7, 8, Table S14, fragment 1) (Figure S7A,B). Moreover, cysteines 36, 65, and 229 were detected in both normal (Table S15, fragments 2–4, and Table S16, fragments 6, 37) and trioxidized form (Table S13, fragments 3–5, and Table S14, fragment 2) (Figure S8A–C), with an Ox/Red ratio of 0.06, 0.7, and 0.3, respectively (Table 1, Table 2, Tables S17 and S18).

In addition, complete carboxyamidomethylation of cysteines 8 and 122 was determined (Table S15, fragment 1; Table S16, fragments 1–4; Table S13, fragment 7). As in NSC34-SOD1WT cells, Cys^2^ was identified in both reduced and acetylated form (Table S15, fragment 1; Table S16, fragments 1–3) (Figure S9), and both oxidized to sulfonic acid and were not acetylated (Table S13, fragment 1) (Figure S10).

### 2.4. Other Post-Translational Modifications of VDAC3

#### 2.4.1. Succination, Ubiquitin and Ubiquitination, Phosphorylation, Citrullination, Cysteinylation, and Dioxidation

In addition to oxidations, other types of PTMs were sought in VDAC3 purified from all NSC34 cell lines analyzed. Specifically, we ascertained that in each of them, Cys^65^ is succinated (Tables S19A–C and S20A–C) and in the NSC34-SOD1G93A ALS model cell line, this modification was interestingly present in greater amounts than in control cells (Table 3 and Figure S11A–C).

Furthermore, no ubiquitin and ubiquitination of lysine residues, phosphorylation of serine, threonine or tyrosine, citrullination of arginine, cysteinylation, and dioxidation of methionines to sulfone were found.

#### 2.4.2. Identification of Deamidation Sites

**Figure 3:**
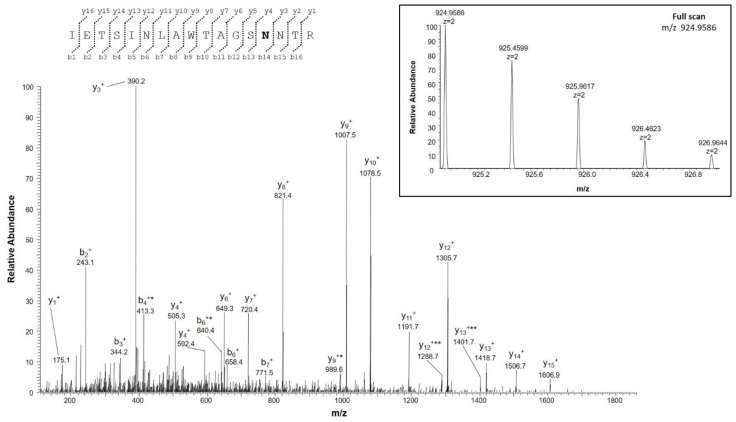
MS/MS spectrum of the doubly charged molecular ion at *m/z* 924.9586 (calculated 924.9582) of the VDAC3 tryptic peptide from NSC34-SOD1G93A cell line containing the asparagine residue 215 in the deamidated form. The inset shows the full scan mass spectrum of molecular ion. Fragment ions originated from the neutral loss of H_2_O are indicated by an asterisk. Fragment ions originated from the neutral loss of NH_3_ are indicated by two asterisks.

In this study, deamidation of asparagine and glutamines were identified exclusively in VDAC3 from the NSC34-SOD1G93A cell line, although deamidated glutamines were found in low amounts and the relative deamidate/normal ratio was, thus, not determined. Interestingly, we found that Asn^215^ was converted to aspartate in significant amounts (deam/normal ratio 0.1) only in ALS cell model (Table 4 and Table S21, Table S22, fragments 4; Figure 3).

In contrast, asparagines 167, 238 and 239 were deamidated at minor level (deam/normal ratio 0.01) (Table 4 and Table S21, Table S22, fragments 3 and 5; Figure S12A–C), and asparagines 76, 106 and 168 were converted to aspartate only in trace amounts (deam/norm ratio 0.002–0.003) (Table 4 and Table S21, Table S22, fragments 1, 2, 3; Figure S12D–F).

In addition to Asn/Asp deamidation events, we analyzed VDAC3 for the presence of succinimide. Usually, succinimide intermediates are formed in the cell as a result of spontaneous deamidation of asparagine and dehydration of aspartic acid [28], and its accumulation represents a sign of aging or a response to cellular stress condition [29]. We identified succinimide at positions 167 and 168 of VDAC3 from all three cell lines studied (Tables S23–S25; Figure S13A–G), although with a different succ/norm ratio, and at position 238 only in the NSC34-SOD1WT cell line (Table S24, fragment 2; Figure S13H). It is worth noting that in the control NSC34 and NSC34-SOD1WT cell lines, asparagine-derived succinimide intermediates were visible in trace amounts (suc/norm ratio ranging from 0.002:1 to 0.004:1) (Table 5, Tables S26 and S27), whereas in the ALS model cell line NSC34-SOD1G93A they were observed in significantly larger amounts (Table 5 and Table S28).

Furthermore, the tryptic peptide Trp^75^-Lys^90^ containing Asp^76^ as a succinimide intermediate and Asp^79^ in the form of aspartate methyl ester, derived from the hydrolysis of succinimide to L-IsoAsp followed by enzymatic methylation [30], was detected only in the NSC34 and NSC34-SOD1WT cell lines (Table 6 and Figure S14A,B).

### 2.5. Bioinformatic Prediction of VDAC3 N215D Mutant Structure

To evaluate the stability of VDAC3 N215D mutant, a computational structural prediction analysis was performed. The human VDAC3 (hVDAC3) structure was rebuilt by homology modelling starting from the VDAC1 PDB file (the crystallographic structure of VDAC3 has not yet been determined), while the VDAC3 N215D mutation was inserted using, as reference, the VDAC3 PDB file previous generated. Specifically, we used a simulation of the structure of human VDAC3 as the reference structure given the high percentage of identity (98.58%) and similarity (99%) with the mouse (Figure S15).

**Figure 4:**
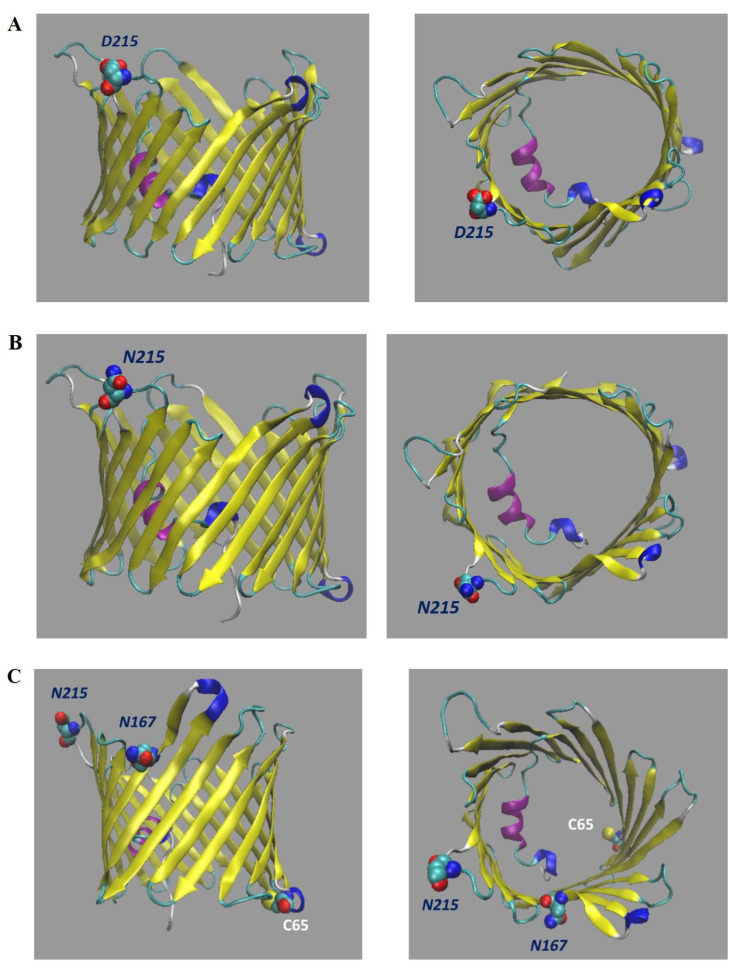
(**A**) Structural predictions of hVDAC3 N215D. Side view (on the left) and top view (on the right) were obtained by VMD software. In both figures, the top side represent the cytosolic environment. (**B**) Structural predictions of hVDAC3 wild type. Side view (on the left) and top view (on the right) were obtained by VMD software. In both figures, the top side represent the cytosolic environment. (**C**) Structural predictions of hVDAC3 N215D showing the localization of N167 and Cys65 residues. Side view (on the left) and top view (on the right) were obtained by VMD software. In both figures, the top side represent the cytosolic environment.

To assess the effect of the N215D mutation on channel conformation and stability, the tertiary structure model of human VDAC3 (hVDAC3) taken as a reference (Q9Y277.1) was modified by replacing Asn^215^ with aspartate, thus mimicking deamidation at the same position (Figure 4A,B).

Ramachandran plots (RP) were also generated to evaluate the predict conformation of the mutated VDAC3 channel by determining the orientation of each amino acid residue relative to the reference wild-type (wt) structure (Figure 4C).

The RP for VDAC3 wt indicates that almost all amino acid residues are distributed in favored regions (95.01%) (Table S29 and Figure S16). The remaining residues are either arranged in allowed regions (4.27%) but with a very high degree of stericity, or distributed in outlier (not allowed) conformations (0.72%).

The three amino acids located in regions are disallowed in the RP of VDAC3 N215D are Gly^23^, Gly^38^, Lys^53^. These amino acids are found spread out in the channel and away from the Asn^215^ residue (Figure S17). Therefore, it is unlikely that these residues can influence the structure of the channel or have an effect on Asn^215^, also because two of the three residues are glycine.

No major differences were observed in amino acid arrangement by comparing the RP of VDAC3 wt with that of VDAC3 N215D (Figure 4C). The percentages of each individual region of both RP are shown in Table S29.

Overall, the single deamidation event on Asn^215^ does not seem to affect the channel stability or produce a substantial modification in the VDAC3 channel structure. This result is not surprising, considering that the mutated residue resides not in the pore wall or in the alpha-helical stretch but in a loop.

### 2.6. Electrophysiological Properties of VDAC3 N215D Mutant

**Figure 5:**
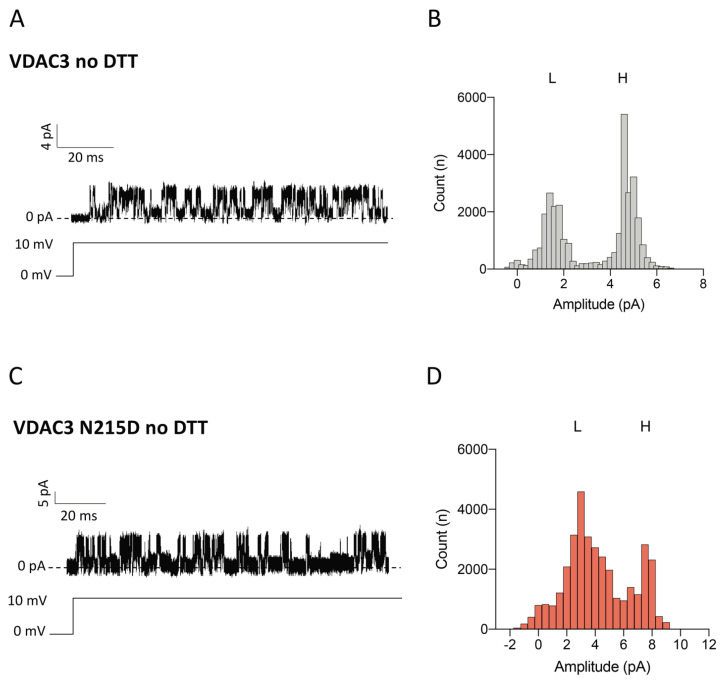
Amplitude distribution of VDAC3 wt and N215D channels in non-reducing conditions. (**A**) Representative current trace of VDAC3 wt in 1 M KCl at + 10 mV applied. (**B**) Amplitude histograms of single channel recording data of VDAC3 wt. (**C**) Representative current trace of VDAC3 N215D in 1 M KCl at +10 mV applied. (**D**) Amplitude histograms of single channel recording data of VDAC3 N215D.

Considering the specific PTMs found in VDAC3 from the NSC34 SOD1-G93A cell line, we wanted to investigate their possible impact on channel functionality. Since it is impossible to produce recombinant proteins with specific oxidated or succinated residues, we focused on evaluating the effects of the most significant deamidation event found in VDAC3, namely the Asn/Asp substitution at the residue 215 residue. For this purpose, we first expressed, purified and refolded the mVDAC3 N215D mutant. Then, using a Planar Lipid Bilayer (PLB) instrument, the electrophysiological properties of the VDAC3 mutant were thoroughly investigated after reconstitution in a lipid bilayer. Measurements of single channel conductance, registered in 1 M KCl upon non-reducing conditions, revealed a slight increase in the pore diameter of VDAC3 mutant (approx. 600 pS) compared to the wild-type protein, mVDAC3 wt (approx. 450 pS). Histograms of the amplitude values as a function of the number of events indicate that N215D mutation also affects electrophysiological stability of the channel (Figure 5). As shown in Figure 5B,D, the presence of Asp^215^ residue caused a different distribution of low-and high-conducting states of VDAC3. Under applied voltage (+10 mV), the mutant protein widened the distribution of the amplitude values corresponding to low-conducting state/s (L), concurrently doubling the conductance in the low-conductance state to approx. 3 pA and abolishing the main peak of VDAC3 wild type (VDAC3 wt) high conducting state (H). These results suggest that VDAC3 N215D undergoes more rapid fluctuations between the high and the low conducting state/s than the wild-type protein. The effect of asparagine deamination on the voltage dependence of VDAC3 was monitored by triangular voltage ramps in the presence or absence of DTT.

**Figure 6:**
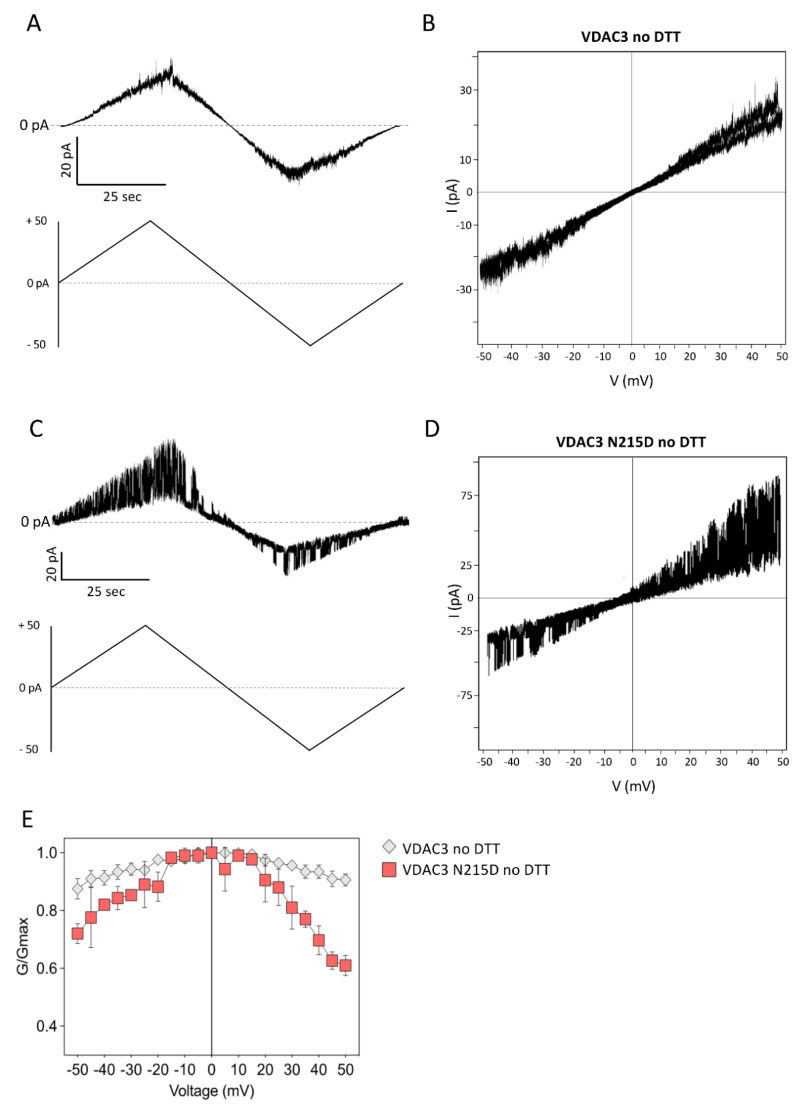
Voltage dependence analysis of VDAC3 wt and N215D in non-reducing conditions. (**A**) Voltage ramp from 0 to ± 50 mVA of VDAC3 wt. (**B**) Current vs. voltage (I–V) plot of VDAC3 wt. (**C**) Voltage ramp from 0 to ± 50 mVA of N215D VDAC3. (**D**) Current vs. voltage (I–V) plot of of N215D VDAC3. (**E**) Voltage dependence graph of wt and mutated VDAC3. The normalized average conductance G/G_0_ was plotted as a function of applied voltage (Vm).

As already reported elsewhere [31,32], in non-reducing conditions, the VDAC3 was found to be insensitive to the applied voltage as the channel current steadily increased and decreased in the range of ±50 mV (Figure 6A) without any variation in the slope of the current vs. voltage (I–V) plot (Figure 6B).

Consistent with the previous findings, VDAC3 N215D demonstrated a dramatic fluctuation in pore conductance upon raising the transmembrane voltage: continuous and very fast switching from open to closed states was observed mostly at positive potential (Figure 6C). The I–V plot shown in Figure 6D further emphasizes the current transitions corresponding to substantial changes in the slope of the curve. The graph of the normalized average conductance G/G_0_ plotted as a function of membrane voltage (Vm) (Figure 6E) depicts an almost flat line for wild-type VDAC3.

Vice versa, the G/G_0_ ratio of VDAC3 N215D does not remain constant and looks more like the characteristic bell-shaped curve of VDAC channels, with lower conductance values at higher membrane potentials.

**Figure 7:**
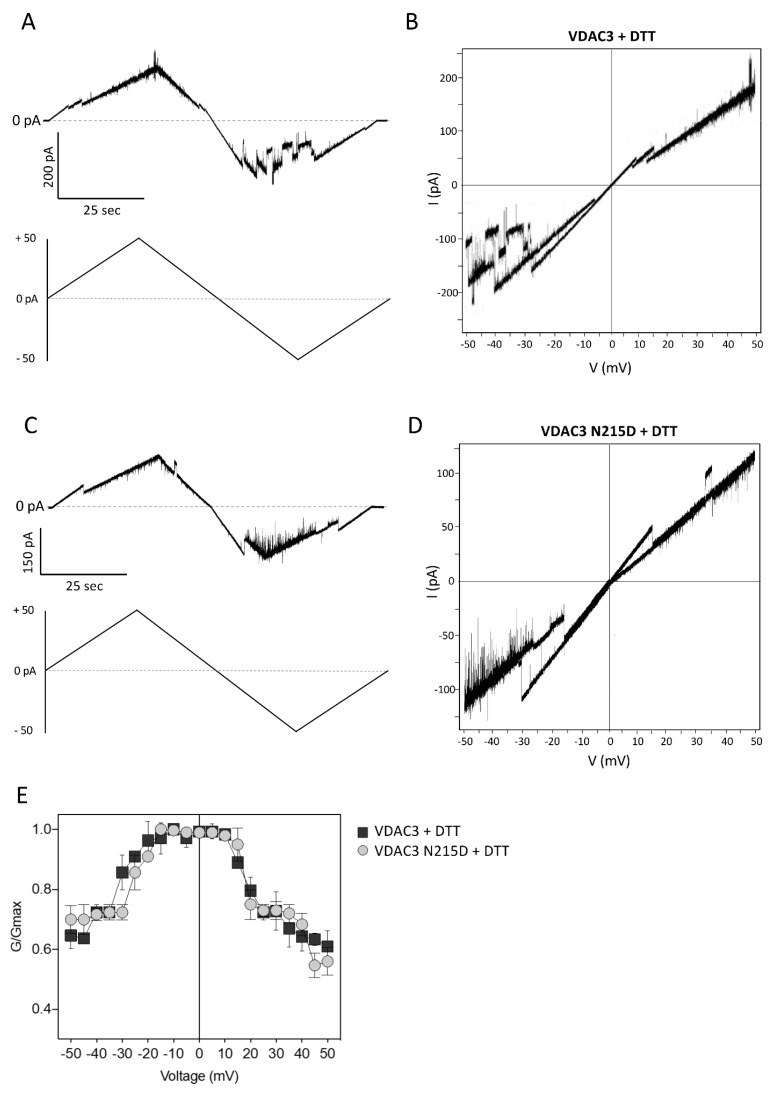
Voltage dependence analysis of VDAC3 wt and N215D preincubated with 5 mM DTT. (**A**) Voltage ramp from 0 to ± 50 mVA of VDAC3 wt. (**B**) Current vs. voltage (I–V) plot of VDAC3 wt. (**C**) Voltage ramp from 0 to ± 50 mVA of N215D VDAC3. (**D**) Current vs. voltage (I–V) plot of N215D VDAC3. (**E**) Voltage dependence graph of wt and mutated VDAC3. The normalized average conductance G/G_0_ was plotted as a function of applied voltage (Vm).

Voltage dependence analysis was also performed after pre-incubating VDAC3 wt and N215D with 5 mM DTT. As shown in Figure 7A,B, treatment of the protein with a reductant completely changes the current profile of VDAC3 in response to a voltage ramp from 0 to ± 50 mV: channel closure is readily observed at both positive and negative potentials. Under these experimental conditions, however, there are no appreciable differences in the voltage response of VDAC3 N215D (Figure 7C,D) compared with the wild-type protein, as demonstrated by the current vs. voltage (I–V) plot of the mutant that almost completely overlaps those of VDAC3 wt (Figure 7E). The only remark is that negative voltages starting from—40 mV close the VDAC3 N215D channel more steadily than the wild-type one (Figure 7B,D). 

## 3. Discussion

Multiple dysregulated mechanisms have been implicated in the pathogenesis of ALS, as of several neurodegenerative diseases. In particular, a mechanistic interplay between protein misfolding, oxidative stress and mitochondrial dysfunction in ALS has been highlighted. VDAC channel, the main protein at the outer mitochondrial membrane, is known to interact with SOD1 mutants linked to ALS. Recently, a specific range of non-enzymatic PTMs were identified in VDAC1 from NSC34-SOD1G93A cells, suggesting the appearance of important structural changes in the channel well correlated with defective mitochondrial metabolism in ALS motor neurons.

Unlike enzymatic PTMs, non-enzymatic PTMs mostly include irreversible chemical modifications on proteins, mediated by reactive compounds as ROS [33,34]. Despite these PTMs represent signs of aging or endogenous cell chemical damage, their role in neurodegenerative contexts has not yet been properly investigated.

Considering all of the above, in this work we investigated PTMs of the VDAC3 isoform from the NSC34 SOD1-G93A ALS cell model. Although the high sequence similarity between VDAC1 and VDAC3 is indicative of a similar pore-forming structure, a physiological unique role of redox sensor has been suggested for the VDAC3 isoform [23,35].

A function also supported by previous interactomics data shows the involvement of VDAC3 in important cellular signaling pathways [36].

Additionally, of interest are scientific validations of interactions between the SOD1 enzyme (whose mutants cause ALS1) and various VDAC3 interactors (PRDX5, TBP1, 37LRP, EF1D) reported at https://www.uniprot.org, accessed on 1 December 2021. Taken together, this information, combined with the already known presence of specific PTMs in VDAC1 from NSC34 SOD1G93A, suggests a role of the VDAC3 isoform in the pathogenesis of ALS.

In this work, to identify PTMs in VDAC3, we used high-resolution mass spectrometry [19,20,25,26], an extremely refined tool that provides particularly useful information for the interpretation of the biological functions of the proteins studied. Moreover, thanks to an “*in-solution*” digestion of the HTP eluate, we overcame the problems associated with the purification of membrane proteins, such as their very high hydrophobicity and low solubility. In addition, the difficulty in isolating individual VDAC isoforms resulted in the need to analyze them as components of a complex mixture, with HRMS investigation of the VDAC3 protein being particularly difficult because this isoform is generally the least expressed among VDACs. More importantly, these refined technical procedures avoided the dangers of unwanted oxidation due the sample preparation step as in [20].

In this study we obtained an excellent sequence coverage (about 99%), with the only uncovered moieties corresponding to Arg^119^-Arg^120^ and Tyr^225^. Furthermore, the oxidation state of methionine and cysteine residues was precisely determined. Interestingly, we found in the VDAC3 sequence both Met^26^ and Met^155^ fully oxidized to methionine sulfoxide exclusively in NSC34-SOD1G93A cells, while in non-ALS control cell lines they were found to be partially oxidized. Met^26^ localizes in the cytosolic rim of VDAC3, at the beginning of the first beta strand and facing partly inward in the pore. A position that could expose this residue to the effect of excess of ROS accumulated in the compartments as a result of pathology. As VDAC1 from the same ALS cell model [20], oxidative modifications of Met^155^ could be indeed explained by its localization in β-sheet 10, facing the lipid bilayer. Thus, it is possible that OMM lipids peroxidized by ALS pathogenesis [37] can modify susceptible residues oriented towards the membrane.

We found no relevant differences in the over-oxidation of cysteines between VDAC3 purified from ALS model or non-ALS NSC34 cells. Indeed, Cys^36^, Cys^65^ and Cys^229^, all of which faced the IMM, were detected either in tri-oxidized and reduced (carboxyamidomethylated) forms in both ALS and control cells, with a similar Ox/Red ratio for homologous cysteines. This most likely means that the level of cysteine oxidation has already reached the maximum stage compatible with the functionality of the protein.

Furthermore, Cys^8^ and Cys^122^, respectively located in the N-terminal alpha helix and within an IMM-facing loop, are always in the carboxyamidomethylated form, thus suggesting their potential availability to form disulfide bridges.

In addition, Cys^2^ was found to be both reduced and acetylated (the “normal” situation) and, unexpectedly, oxidized to sulfonic acid and was not acetylated, neither in cells expressing human SOD1 wt nor the mutated form. The presence of a cysteine at the N-terminus is a unique feature of the VDAC3 isoform. Evidently, just the peculiar positioning of this cysteine at the beginning of the N-terminal alpha helix could already promote its over-oxidation in cells that over-express SOD1wt. Indeed, high levels of native SOD1 are known to result in misfolding and aggregation of SOD1 itself, correlating with forms of sALS [5].

The oxidative stress condition, typical of all forms of ALS, as well as the results obtained in the previous work on VDAC1 [20], prompted us to search the sequence of VDAC3 for asparagine and glutamine deamidation events in addition to oxidations. Asparagine and glutamine deamidation are spontaneous, non-enzymatic and post-biosynthetic modifications affecting structure, stability, folding, and aggregation of proteins [38,39,40,41]. Besides playing a role in cataract formation, its importance has also been established in Alzheimer’s, Parkinson’s, and ALS as other degenerative diseases [20,42,43,44]. Moreover, being a modification that proteins accumulate with cellular aging and following pathological conditions, it could be useful as a biomarker of disease [45,46]. The deamidation mechanism involves the formation of an unstable cyclic succinimidyl intermediate, which is hydrolyzed to yield Asp or isoAsp, with an approximate ratio of 3:1 [47,48]. Isoaspartic acid is an isomer of aspartic acid with the C_β_ incorporated into the backbone. These isopeptide bonds are known to impair protein structure/function or enhance the aggregation process, contributing to protein aging and folding disorders typical of neurodegenerative diseases. In this regard, it has been reported that in erythrocytes of ALS patients there is an accumulation of L-isoaspartyl residues [30]. Consistent with the known data, our analysis showed a higher amount of succinimidyl intermediate in the VDAC3 of the NSC34-SOD1G93A cell line than in the other two NSC34 non-SLA cell lines. Additionally, we detected the presence of asparagine 79 as aspartate methyl ester in the NSC34 cells and in those expressing the SOD1 wt, but not in the cell line expressing the mutated SOD1 G93A. This can be explained considering that the free α-carbonyl group of the dangerous L-isoAsp is methylated by the enzyme L-isoaspartyl/(D-asp) methyltransferase (PIMT) and S-adenosylmethionine (AdoMet) as a methyl donor. Subsequently, hydrolysis of the methyl ester leads to the reformation of the intermediate L-succinimide which is then partly hydrolyzed to L-Asp, thus completing the physiological repair reaction that counteracts the accumulation of dysfunctional proteins [49,50,51]

In addition, only in NSC34-SOD1G93A cells did we detect very high levels (10% of molecules) of deamidated Asn^215^ (Table 4 and Figure 4C). Considering the potential impact of this modification on protein structure/function, we assessed the stability of the VDAC3 N215D mutant by performing a molecular modeling analysis. For this purpose, we replaced Asn^215^ with an aspartate in the structure model of human VDAC3. The results show no significant changes in the pore structure of the VDAC3 N125D mutant. In both Ramachandran plots of VDAC3 wt and N215D there is a predominance of residues located in the β region, suggesting for both structures the typical β-barrel motif that characterizes all isoforms of VDAC (Figure S15). Notably, the Asn/Asp substitution would not seem to result in any change in structure, and this could be due to the position that residue 215 has within the structure. Indeed, this asparagine is located in the center of a loop connecting two β-strands (Figure 4C), with the side chain protruding toward the cytosol, with no apparent involvement in the formation of the pore wall. In addition, results on mVDAC3 N215D were produced using the model of human VDAC3, reconstructed by homology to the crystallographic structure of human VDAC1, as the reference structure. This model, although predictively very similar, may, in reality, have some differences.

Then, considering the little information provided by homology analysis, we wanted to further investigate whether deamidation of Asn215 was capable of producing effects on protein function. Therefore, we produced, purified and folded the mVDAC3 N215D mutant protein and analyzed its electrophysiological characteristics at PLB. Through this analysis, we ascertained that the N215D mutation results in a significative increase in the fluctuation of pore conductance under non-reducing conditions. In contrast, the voltage dependence analysis performed under reducing conditions indicates that the VDAC3 mutant has a behavior mostly overlapping with the wt protein. Furthermore, VDAC3 N215D shows voltage dependence already in non-reducing conditions, at a variance of the wt VDAC3, indicating that the residue may be involved in the voltage-sensor structure. On the other hand, in reducing conditions, no special difference between the wt and pathological forms are strikingly apparent.

As previously pointed out, Asn^215^ localizes in a loop formed by six amino acids (212–217) and is exposed toward the cytosolic environment, i.e., in a water-exposed context where ROS can make their effect felt faster. Although the other asparagines of VDAC3 would also appear to be mostly exposed toward the cytosolic environment, they exhibit lower levels of deamidation than Asn^215^. This difference might depend on the nearby amino acids as well as on the specific interactors with which each residue is physiologically engaged [35]. Specifically, Asn^215^ might be in a suitable position to establish interactions with cytosolic molecules important for VDAC3 function and whole organelle homeostasis.

It is noteworthy that the higher level of succinimide intermediate was detected for Asn^167^ (much less for Asn^168^) of VDAC3 from ALS model cells compared with the protein purified from control lines (Table 5). This finding could depend on the fact that the aspartyl and iso-aspartyl residues produced by deamidation of Asn^167^ dehydrate faster (or hydrate more slowly) in the reaction that still reforms the intermediate succinimide. The reduced levels of Asp produced by deamidation from Asn^167^ could be explained as follows, as the positioning in the pore of this residue could also favor the formation of the intermediate. Indeed, Asn^167^ localizes in the central portion of the 11th beta strand, protruding toward the membrane lipid environment, preceded by two polar amino acids (serine and glutamine) (Figure 4C). The latter condition is favorable for the deamidation reaction. Furthermore, the exposure of Asn^167^ toward the lipid bilayer allows us to hypothesize that OMM lipids peroxidized by ALS pathogenesis [36] may modify sensitive membrane-oriented residues, as previously reported for VDAC1 from the same cell type [20].

Another interesting result, only discovered in the VDAC3 from NSC34-SOD1G93A cells, concerns the much higher levels of Cys^65^ succination compared with the control NSC34 cell lines. Succination, or the formation of S-(2-succino) cysteine (2SC), is a non-enzymatic and irreversible modification that physiologically occurs by fumarate adduction to the sulfhydryl group of Cys only, through a Michael-type reaction [52]. Succination preferentially targets cysteine residues with low pKa values (up to 3–4), such as those located in protein active sites or placed in a suitable biological context [53]. For this reason, 2SC is a recognized biomarker of metabolic stress and, more specifically, mitochondrial stress. In addition, this PTM increases with aging, when there is an excess of nutrients, a high ATP/ADP and/or NADH/NAD+ ratio, or, more generally, when there is an abnormal increase in Krebs cycle intermediates. It is, therefore, associated with mitochondrial dysfunction and increased ROS, resulting in neuronal damage [54].

It is, thus, not surprising that we found high levels of succination in VDAC3 of the ALS model cell line. Specifically, succinated Cys^65^ found in VDAC3 is located on the edge of the pore looking toward the IMM, usually in itself a highly oxidizing environment, but even more exacerbated in the ALS pathological context due to the impairment of antioxidant defenses. It is noteworthy that several proteins known to interact with VDAC3 have been found succinated in other cell types [35,55]. Interestingly, among the interactors of VDAC3, succination of Protein Disulfide Isomerase was reported, thus providing a link between mitochondrial stress and endoplasmic reticulum stress [56]. Succination of α- and β-tubulins is also known [57] as a mechanism that can inhibit their polymerization and, thus, alter mitochondrial dynamics. The latter aspect has an important consequence for ALS, and neurodegeneration in general, as neurons are cells incredibly dependent on their cytoskeletal transport mechanism. Indeed, in the cell body and axons of mature neurons, the cytoskeleton enables fundamental processes, such as movement of every component, delivery of new proteins and organelles to distal sites, as well as removals of aged ones [58].

## 4. Materials and Methods

### 4.1. Chemicals

All chemicals were of the highest purity commercially available and were used without further purification. Ammonium bicarbonate, calcium chloride, phosphate-buffered saline (PBS), Tris-HCl, Triton X-100, sucrose, mannitol, ethylene glycol tetraacetic acid (EGTA), ethylenediaminetetraacetic acid (EDTA), formic acid (FA), dithiothreitol (DTT) and iodoacetamide (IAA) were obtained from Sigma-Aldrich (Milan, Italy). High-glucose DMEM (Dulbecco’s Modified Eagle Medium) and fetal bovine serum (FBS) were obtained from Gibco-Thermo Fisher Scientific (Milan, Italy). DMEM F12 and tetracycline-free FBS were obtained from Euro Clone. G418 and Doxycycline were obtained from Carlo Erba and Sigma-Aldrich, respectively. Trypsin/EDTA (for cell cultures) and penicillin-streptomycin (P/S) were purchased from Invitrogen. All other stock solutions for cell cultures were from Euroclone (Milan, Italy). Modified porcine trypsin and chymotrypsin were purchased from Promega (Milan, Italy). Water and acetonitrile (OPTIMA^®^ LC/MS grade) for LC/MS analyses were provided from Fisher Scientific (Milan, Italy).

### 4.2. NSC34 Cell Lines

NSC34 cell lines (mouse motor neuron-like) obtained from CELLutions Biosystem Inc. were stably transfected with pTet-ON plasmid (Clontech, Mountain View, CA, USA) harboring sequences encoding human SOD1 wt (NSC34-SOD1WT) or G93A (NSC34-SOD1G93A) and used as non-ALS motor neuron line and ALS motor neuron line, respectively [59]. The cell lines were maintained as in Magrì et al. [60].

### 4.3. Extraction of Mitochondrial Proteins from NSC34 Cells under Reducing Conditions

Extraction of mitochondrial proteins from NSC34 cells under reducing conditions was performed as described in [20].

### 4.4. Liquid Chromatography and Tandem Mass Spectrometry (LC–MS/MS) Analysis and Database Search

Mass spectrometry data were acquired and processed as described in [20].

#### Identification of Deamidation Sites on VDAC3

A freely available command-line script for Python 2.x (https://github.com/dblyon/deamidation), accessed on 1 December 2021 which uses the MaxQuant “evidence.txt” file, was used to estimate the percentage of deamidation in VDAC 3 for each cell line, as in [20].

### 4.5. Expression, Purification and Refolding of Recombinant VDAC3 Proteins

The sequence encoding mouse VDAC3 (mVDAC3) was cloned in pET21a vector (Novagen) as reported in [23]. Mutagenesis of mVDAC3 was achieved using a specific couple of primers designed to replace Asn^215^ residue with Asp according to the protocol already described in [61]. The single mutation was confirmed by DNA sequencing.

Vectors containing native or mutagenized mVDAC3 (i.e., VDAC3 wt and VDAC3 N215D, respectively) coding sequence were used to transform Escherichia coli BL21(DE3) cells. Recombinant VDAC3 proteins expression, purification and refolding were carried out as in [11].

### 4.6. Lipid Bilayer Experiments

Planar lipid bilayer experiments were performed as described previously [62,63]. Artificial membranes made of 1% (*w*/*v*) diphytanoyl phosphatidylcholine (DiphPC) (Avanti Polar Lipids, Alabaster, AL, USA) in n-decane were painted on a 200 µm hole in a Derlin cuvette (Warner Instruments, Hamden, CT, USA). All the experiments were carried out at RT. Membrane capacitances of 100–150 pF were established for appropriate lipid bilayers. Mutant or native mVDAC3 were added from the protein stock solution of 1 mg/mL to the cis side of the cuvette filled with symmetrical 1 M KCl/10 mM HEPES pH 7.0. The single channel conductance of the pores was measured upon application of a fixed membrane potential (+10 mV) [58]. The voltage dependence was calculated by applying a triangular voltage ramp from 0 to ±50 mV of 100 ms duration, with a frequency of 10 mHz. At least three independent experiments were performed for each protein. A Bilayer Clamp amplifier (Warner Instruments) at 100 ms/point and filtered at 300 Hz was used for data acquisition. Analyses were performed with the pClamp software (Ver-10; Molecular Devices, San Jose, CA, USA).

### 4.7. Modelling and Bioinformatics Analysis

The structures of VDAC3 N215D shown in this work were obtained computationally with MODELLER software v9.24 [64] using the structure of human VDAC1 WT as a template. N215D mutations were introduced by substitution of the selected amino acid residue in the FASTA sequence. The same software was used for evaluation of the energetic score associated with each structure. Graphical representation was obtained by using VMD—Visual Molecular Dynamic software (available at: https://www.ks.uiuc.edu/Research/vmd/), accessed on 1 January 2022. The root means square deviation (RMSD) analysis and the Ramachandran plots (RPs) were obtained both through the free online software at https://zlab.umassmed.edu/bu/rama/, accessed on 1 January 2022 and by using specific tools in the VMD software.

## 5. Conclusions

In this work, combining HRMS analysis with “*in-solution*” digestion of an enriched VDACs fraction, we found specific and irreversible PTMs in VDAC3 purified from the ALS model cell line. In particular, in VDAC3 we identified channel oxidation, deamidation and succination events and provided experimental evidence of functional changes of deamidated VDAC3. The impact of the most abundant deamidation event was verified by reconstituting its activity in a functional assay.

Overall, our data complete the picture we have drawn with previous results about VDAC1 from the same cell type. Specifically, the post-translational modifications identified and discussed here may represent sufficient conditions to alter the physiological pool of interactors of VDAC3 and modify its specific ability to buffer ROS, possibly impacting IMM redox signaling in ALS. 

## Figures and Tables

**Table 1:** Ox/Red ratio (average and standard deviation) of the absolute intensities of the molecular ions of sulfur containing tryptic peptides found in the analysis of VDAC3 from NSC34, NSC34-SOD1WT, NSC34-SOD1G93A cell lines reduced with DTT, carboxyamidomethylated and digested in-solution.

Peptide	Position in the Sequence	Calculated Monoisotopic *m/z*	Cell Lines	Ratio Ox/Red	
				Avg	Dev St
GYGFG**M**VK	21–28	437.7103 (2+)	NSC34	10.1	0.85
GYGFGMVK		429.7128 (2+)	NSC34-SOD1G93A	- Met^26^ totally oxidized	-
S**C**SGVEFSTSGHAYTDTGK	35–53	991.4079 (2+)	NSC34	0.08	0.01
			NSC34-SOD1WT	0.05	0.01
S***C***SGVEFSTSGHAYTDTGK		995.9260 (2+)			
			NSC34-SOD1G93A	0.05	0.01
YKV**C**NYGLTFTQK	62–74	806.8877 (2+)	NSC34	0.87	0.06
			NSC34-SOD1WT	0.6	0.10
YKV***C***NYGLTFTQK		811.4060 (2+)			
			NSC34-SOD1G93A	0.67	0.12
D*C*FSLGSNVDIDFSGPTIYGWAVLAFEGWLAGYQ**M**SFDTAK	121–161	1518.0315 (3+)	NSC34-SOD1WT	6.63	1.33
D*C*FSLGSNVDIDFSGPTIYGWAVLAFEGWLAGYQMSFDTAK		1512.6995 (3+)	NSC34-SOD1G93A	- Met^155^ totally oxidized	-

***C***: cysteine carboxyamidomethylated; **C**: cysteine oxidized to sulfonic acid; **M**: methionine sulfoxide.

**Table 2:** Ox/Red ratio (average and standard deviation) of the absolute intensities of the molecular ions of sulfur containing chymotryptic peptides found in the analysis of VDAC3 from NSC34, NSC34-SOD1WT, NSC34-SOD1G93A cell lines reduced with DTT, carboxyamidomethylated and digested in-solution.

Peptide	Position in the Sequence	Calculated Monoisotopic *m/z*	Cell Lines	Ratio Ox/Red	
				Avg	Dev St
GFG**M**VKIDL	23–31	498.2657 (2+)	NSC34	11.73	2.23
			NSC34-SOD1WT	12.07	1.27
GFGMVKIDL		490.2682 (2+)			
			NSC34-SOD1G93A	- Met^26^ totally oxidized	-
Q**M**SFDTAKSKL	154–164	636.3192 (2+)	NSC34	0.50	0.30
QMSFDTAKSKL		628.3217 (2+)			
KLD**C**RTSL	226–233	492.2455 (2+)	NSC34	0.10	0
			NSC34-SOD1WT	0.53	0.15
KLD***C***RTSL		496.7638 (2+)			
			NSC34-SOD1G93A	0.33	0.15

***C***: cysteine carboxyamidomethylated; **C**: cysteine oxidized to sulfonic acid; **M**: methionine sulfoxide.

**Table 3:** Ox/Red ratio (average and standard deviation) of the absolute intensities of the molecular ions of tryptic peptides containing succinated and non-succinated cysteines found in the analysis of VDAC3 from NSC34, NSC34-SOD1WT, NSC34-SOD1G93A cell lines reduced with DTT, carboxyamidomethylated and digested in-solution.

Peptide	Position in the Sequence	Calculated Monoisotopic *m/z*	Cell Lines	Ratio Succinated/Red	
				Avg	Dev St
YKV$\underset{=}{C}$NYGLTFTQK	62–74	560.9367 (3+)	NSC34	0.3	0
			NSC34-SOD1WT	0.40	0.10
YKV***C***NYGLTFTQK		811.4060 (2+)			
			NSC34-SOD1G93A	1.07	0.12

***C***: cysteine carboxyamidomethylated; $\underset{=}{C}$: cysteine succinated.

**Table 4:** Ox/Red ratio (average and standard deviation) of the absolute intensities of the molecular ions of tryptic peptides containing deamidated and non-deamidated asparagine found in the analysis of VDAC3 from NSC34-SOD1G93A cell line reduced with DTT, carboxyamidomethylated and digested in-solution.

Peptide	Position in the Sequence	Calculated Monoisotopic *m/z*	Ratio Deam/Norm	
			Avg	Dev St
W**N**TDNTLGTEISWENK	75–90	954.9344 (2+)	0.003	0
WNTDNTLGTEISWENK		954.4424 (2+)		
LTLDTIFVP**N**TGK	97–109	710.3904 (2+)	0.003	0
LTLDTIFVPNTGK		709.8984 (2+)		
LSQ**N**NFALGYK	164–174	628.3197 (2+)	0.01	0
LSQNNFALGYK				
LSQN**N**FALGYK		627.8277 (2+)	0.003	0
LSQNNFALGYK				
IETSINLAWTAGS**N**NTR	202–218	924.9582 (2+)	0.1	0
IETSINLAWTAGSNNTR		924.4662 (2+)		
V**N**NASLIGLGYTQTLRPGVK	237–256	701.3920 (3+)	0.01	0
VNNASLIGLGYTQTLRPGVK				
VN**N**ASLIGLGYTQTLRPGVK		701.0640 (3+)	0.01	0
VNNASLIGLGYTQTLRPGVK				

**N**: asparagine deamidated.

**Table 5:** Ox/Red ratio (average and standard deviation) of the absolute intensities of the molecular ions of tryptic peptides containing succinimide intermediate found in the analysis of VDAC3 from NSC34, NSC34-SOD1WT, NSC34-SOD1G93A cell lines reduced with DTT, carboxyamidomethylated and digested in-solution.

Peptide	Position in the Sequence	Calculated Monoisotopic *m/z*	Cell Lines	Ratio Succinimide/Norm	
				Avg	Dev St
LSQ$\underset{=}{\mathrm{NN}}$FALGYK	164–174	619.3144 (2+)	NSC34	0.002	0
			NSC34-SOD1WT	0.003	0
LSQNNFALGYK		627.8277 (2+)			
			NSC34-SOD1G93A	0.01	0
V$\underset{=}{N}$NASLIGLGYTQTLRPGVK	237–256	1042.5788 (2+)	NSC34-SOD1WT	0.003	0
VNNASLIGLGYTQTLRPGVK		1051.0924 (2+)			

$\underset{=}{N}$: succinimide intermediate; $\underset{=}{\mathrm{NN}}$: N167 or N168 residue in the succinimide intermediate form.

**Table 6:** Retention time, experimentally measured and calculated monoisotopic *m/z* of the molecular ions, position in the sequence, absolute intensities and peptide sequence of tryptic fragment containing asparagine residue 76 in succinimide intermediate form and asparagine residue 79 in isoaspartate methyl ester form found in the analysis of VDAC3 from NSC34 and NSC34-SOD1WT cell lines reduced with DTT, carboxyamidomethylated and digested in-solution.

Cell Lines	Peptide	Position in the Sequence	Rt (min)	Calculated Monoisotopic *m/z*	Absolute Intensity
NSC34	W$\underset{=}{N}$TD$\underset{﹋}{N}$TLGTEISWENK	75–90	52.07	953.4408 (2+)	6.7 · 10^3^
			51.55		3.9 · 10^3^
NSC34-SOD1WT			51.56		1.1 · 10^5^
			51.32		1.3 · 10^5^
			51.04		6.1 · 10^4^

$\underset{=}{N}$: succinimide intermediate; $\underset{﹋}{N}$: isoaspartate methyl ester.

## Data Availability

Data are available via ProteomeXchange with identifier PXD036728.

## Supplementary Materials

The following supporting information can be downloaded at: www.mdpi.com/article/10.3390/ijms232415853/s1.

## References

[1] Rowland L.P., Shneider N.A. (2001). Amyotrophic Lateral Sclerosis. N. Engl. J. Med..

[2] Pasinelli P., Brown R.H. (2006). Molecular biology of amyotrophic lateral sclerosis: Insights from genetics. Nat. Rev. Neurosci..

[3] Brown R.H., Al-Chalabi A. (2017). Amyotrophic Lateral Sclerosis. N. Engl. J. Med..

[4] Kaur S.J., McKeown S.R., Rashid S. (2016). Mutant SOD1 mediated pathogenesis of Amyotrophic Lateral Sclerosis. Gene.

[5] Sanghai N., Tranmer G.K. (2021). Hydrogen Peroxide and Amyotrophic Lateral Sclerosis: From Biochemistry to Pathophysiology. Antioxidants.

[6] Prudencio M., Borchelt D.R. (2011). Superoxide dismutase 1 encoding mutations linked to ALS adopts a spectrum of misfolded states. Mol. Neurodegener..

[7] Mondola P., Damiano S., Sassa A., Santillo M. (2016). The Cu, Zn Superoxide Dismutase: Not Only a Dismutase Enzyme. Front. Physiol..

[8] Wang F., Lu Y., Qi F., Su Q., Wang L., You C., Che F., Yu J. (2014). Effect of the human SOD1-G93A gene on the nrf2/ARE signaling pathway in NSC-34 cells. Mol. Med. Rep..

[9] Marden J.J., Harraz M.M., Williams A.J., Nelson K., Luo M., Paulson H., Engelhardt J.F. (2007). Redox modifier genes in amyotrophic lateral sclerosis in mice. J. Clin. Investig..

[10] Israelson A., Arbel N., Da Cruz S., Ilieva H., Yamanaka K., Shoshan-Barmatz V., Cleveland D.W. (2010). Misfolded Mutant SOD1 Directly Inhibits VDAC1 Conductance in a Mouse Model of Inherited ALS. Neuron.

[11] Shteinfer-Kuzmine A., Argueti S., Gupta R., Shvil N., Abu-Hamad S., Gropper Y., Hoeber J., Magrì A., Messina A., Kozlova E.N. (2019). A VDAC1-Derived N-Terminal Peptide Inhibits Mutant SOD1-VDAC1 Interactions and Toxicity in the SOD1 Model of ALS. Front. Cell. Neurosci..

[12] Shoshan-Barmatz V., De Pinto V., Zweckstetter M., Raviv Z., Keinan N., Arbel N. (2010). VDAC, a multi-functional mitochondrial protein regulating cell life and death. Mol. Asp. Med..

[13] Magri A., Messina A. (2017). Interactions of VDAC with Proteins Involved in Neurodegenerative Aggregation: An Opportunity for Advancement on Therapeutic Molecules. Curr. Med. Chem..

[14] Shoshan-Barmatz V., Ben-Hail D. (2012). VDAC, a multi-functional mitochondrial protein as a pharmacological target. Mitochondrion.

[15] Reina S., Guarino F., Magrì A., De Pinto V. (2016). VDAC3 As a Potential Marker of Mitochondrial Status Is Involved in Cancer and Pathology. Front. Oncol..

[16] Wang Y.-C., Peterson S.E., Loring J.F. (2013). Protein post-translational modifications and regulation of pluripotency in human stem cells. Cell Res..

[17] Duan G., Walther D. (2015). The Roles of Post-translational Modifications in the Context of Protein Interaction Networks. PLoS Comput. Biol..

[18] Reina S., Pittalà M.G.G., Guarino F., Messina A., De Pinto V., Foti S., Saletti R. (2020). Cysteine Oxidations in Mitochondrial Membrane Proteins: The Case of VDAC Isoforms in Mammals. Front. Cell Dev. Biol..

[19] Pittalà M.G.G., Nibali S.C., Reina S., Cunsolo V., Di Francesco A., De Pinto V., Messina A., Foti S., Saletti R. (2021). VDACs Post-Translational Modifications Discovery by Mass Spectrometry: Impact on Their Hub Function. Int. J. Mol. Sci..

[20] Pittalà M.G.G., Reina S., Cubisino S.A.M., Cucina A., Formicola B., Cunsolo V., Foti S., Saletti R., Messina A. (2020). Post-Translational Modification Analysis of VDAC1 in ALS-SOD1 Model Cells Reveals Specific Asparagine and Glutamine Deamidation. Antioxidants.

[21] Magrì A., Reina S., De Pinto V. (2018). VDAC1 as Pharmacological Target in Cancer and Neurodegeneration: Focus on Its Role in Apoptosis. Front. Chem..

[22] Smilansky S., Dangoor L., Nakdimon I., Ben-Hail D., Mizrachi D., Shosha-Barmatz V. (2015). The Voltage-Dependent Anion Channel 1 mediates amyloid beta toxicity and represents a potential target for Alzheimer’s disease theraphy. JBC.

[23] Reina S., Checchetto V., Saletti R., Gupta A., Chaturvedi D., Guardiani C., Guarino F., Scorciapino M.A., Magrì A., Foti S. (2016). VDAC3 as a sensor of oxidative state of the intermembrane space of mitochondria: The putative role of cysteine residue modifications. Oncotarget.

[24] Reina S., Nibali S.C., Tomasello M.F., Magrì A., Messina A., De Pinto V. (2022). Voltage Dependent Anion Channel 3 (VDAC3) protects mitochondria from oxidative stress. Redox Biol..

[25] Saletti R., Reina S., Pittalà M.G., Belfiore R., Cunsolo V., Messina A., De Pinto V., Foti S. (2017). High resolution mass spectrometry characterization of the oxidation pattern of methionine and cysteine residues in rat liver mitochondria voltage-dependent anion selective channel 3 (VDAC3). Biochim. Biophys. Acta (BBA) Biomembr..

[26] Pittalà M.G.G., Saletti R., Reina S., Cunsolo V., De Pinto V., Foti S. (2020). A High Resolution Mass Spectrometry Study Reveals the Potential of Disulfide Formation in Human Mitochondrial Voltage-Dependent Anion Selective Channel Isoforms (hVDACs). Int. J. Mol. Sci..

[27] Guan Z., Yates N.A., Bakhtiar R. (2003). Detection and characterization of methionine oxidation in peptides by collision-induced dissociation and electron capture dissociation. J. Am. Soc. Mass Spectrom..

[28] Cournoyer J.J., Pittman J.L., Ivleva V.B., Fallows E., Waskell L., Costello C.E., O’Connor P.B. (2005). Deamidation: Differentiation of aspartyl from isoaspartyl products in peptides by electron capture dissociation. Protein Sci..

[29] Jové M., Pradas I., Mota-Martorell N., Cabré R., Ayala V., Ferrer I., Pamplona R. (2020). Succination of Protein Thiols in Human Brain Aging. Front. Aging Neurosci..

[30] D’Angelo S., Trojsi F., Salvatore A., Daniele L., Raimo M., Galletti P., Monsurrò M.R. (2013). Accumulation of altered aspartyl residues in erythrocyte membrane proteins from patients with sporadic amyotrophic lateral sclerosis. Neurochem. Int..

[31] Checchetto V., Reina S., Magrì A., Szabo I., De Pinto V. (2014). Recombinant Human Voltage Dependent Anion Selective Channel Isoform 3 (hVDAC3) Forms Pores with a Very Small Conductance. Cell Physiol. Biochem..

[32] Nibali S.C., Di Rosa M.C., Rauh O., Thiel G., Reina S., De Pinto V. (2021). Cell-free electrophysiology of human VDACs incorporated into nanodiscs: An improved method. Biophys. Rep..

[33] Liddy K.A., White M.Y., Cordwell S.J. (2013). Functional decorations: Post-translational modifications and heart disease delineated by targeted proteomics. Genome Med..

[34] Tomin T., Schittmayer M., Honeder S., Heininger C., Birner-Gruenberger R. (2019). Irreversible oxidative post-translational modifications in heart disease. Expert Rev. Proteom..

[35] Okazaki M., Kurabayashi K., Asanuma M., Saito Y., Dodo K., Sodeoka M. (2015). VDAC3 gating is activated by suppression of disulfide-bond formation between the N-terminal region and the bottom of the pore. Biochim. Biophys. Acta.

[36] Messina A., Reina S., Guarino F., Magrì A., Tomasello F., Clark R.E., Ramsay R.R., De Pinto V. (2014). Live cell interactome of the human voltage dependent anion channel 3 (VDAC3) revealed in HeLa cells by affinity purification tag technique. Mol. BioSyst..

[37] Ravera S., Bonifacino T., Bartolucci M., Milanese M., Gallia E., Provenzano F., Cortese K., Panfoli I., Bonanno G. (2018). Char-acterization of the Mitochondrial Aerobic Metabolism in the Pre- and Perisynaptic Districts of the SOD1G93A Mouse Model of Amyotrophic Lateral Sclerosis. Mol. Neurobiol..

[38] Hains P.G., Truscott R.J.W. (2010). Age-Dependent Deamidation of Life long Proteins in the Human Lens. Investig. Ophthalmol. Vis. Sci..

[39] Hooi M.Y.S., Raftery M.J., Truscott R.J.W. (2012). Racemization of Two Proteins over Our Lifespan: Deamidation of Asparagine 76 in γS Crystallin Is Greater in Cataract than in Normal Lenses across the Age Range. Investig. Ophthalmol. Vis. Sci..

[40] Truscott R.J.W. (2010). Are Ancient Proteins Responsible for the Age-Related Decline in Health and Fitness?. Rejuvenation Res..

[41] Lindner H., Helliger W. (2001). Age-dependent deamidation of asparagine residues in proteins. Exp. Gerontol..

[42] Robinson N.E., Robinson M.L., Schulze S.E.S., Lai B.T., Gray H.B. (2009). Deamidation ofα-synuclein. Protein Sci..

[43] Shimizu T., Watanabe A., Ogawara M., Mori H., Shirasawa T. (2000). Isoaspartate Formation and Neurodegeneration in Alzheimer’s Disease. Arch. Biochem. Biophys..

[44] Vigneswara V., Cass S., Wayne D., Bolt E.L., Ray D.E., Carter W.G. (2013). Molecular Ageing of Alpha- and Beta-Synucleins: Protein Damage and Repair Mechanisms. PLoS ONE.

[45] Bastrup J., Kastaniegaard K., Asuni A.A., Volbracht C., Stensballe A. (2020). Proteomicand Unbiased Post-Translational Modification Profiling of Amyloid Plaques and Surrounding Tissue in a Transgenic Mouse Model of Alzheimer’s Disease. J. Alzheimer’s Dis..

[46] Wilmarth P.A., Tanner S., Dasari S., Nagalla S.R., Riviere M.A., Bafna V., Pevzner P.A., David L.L. (2006). Age-related changes in human crystallins determined from comparative analysis of post-translational modifications in young and aged lens: Does deamidation contribute to crystallin insolubility?. J. Proteome Res..

[47] Catak S., Monard G., Aviyente V., Ruiz-Lo´pez M.F. (2009). Deamidation of Asparagine Residues: Direct Hydrolysis versus Succinimide-Mediated Deamidation Mechanisms. J. Phys. Chem..

[48] Geiger T., Clarke S. (1987). Deamidation, isomerization, and racemization at asparaginyl and aspartyl residues in peptides. Succinimide-linked reactions that contribute to protein degradation. J. Biol. Chem..

[49] Reissner K.J., Aswad D.W. (2003). Deamidation and isoaspartate formation in proteins: Unwanted alterations or surreptitious signals?. Cell Mol. Life Sci..

[50] Shimizu T., Matsuoka Y., Shirasawa T. (2005). Biological Significance of Isoaspartate and Its Repair System. Biol. Pharm. Bull..

[51] Galletti P., De Bonis M.L., Sorrentino A., Raimo M., D’Angelo S., Scala I., Andria G., D’Aniello A., Ingrosso D., Zappia V. (2007). Accumulation of altered aspartyl residues in erythrocyte proteins from patients with Down’s syndrome. FEBS J..

[52] Alderson N.L., Wang Y., Blatnik M., Frizzell N., Walla M.D., Lyons T.J., Alt N., Carson J.A., Nagai R., Thorpe S.R. (2006). S-(2-Succinyl)cysteine: A novel chemical modification of tissue proteins by a Krebs cycle intermediate. Arch. Biochem. Biophys..

[53] Saletti R., Reina S., Pittalà M.G., Magrì A., Cunsolo V., Foti S., De Pinto V. (2018). Post-translational modifications of VDAC1 and VDAC2 cysteines from rat liver mitochondria. Biochim. Biophys. Acta Bioenerg..

[54] Guan R., Wang J., Cai Z., Li Z., Wang L., Li Y., Xu J., Li D., Yao H., Liu W. (2020). Hydrogen sulfide attenuates cigarette smoke-induced airway remodeling by upregulating SIRT1 signaling pathway. Redox Biol..

[55] Merkley E.D., Metz T.O., Smith R.D., Baynes J.W., Frizzell N. (2014). The succinated proteome. Mass Spectrom. Rev..

[56] Manuel A.M., Walla M.D., Faccenda A., Martin S.L., Tanis R.M., Piroli G.G., Adam J., Kantor B., Mutus B., Townsend D.M. (2017). Succination of Protein Disulfide Isomerase Links Mitochondrial Stress and Endoplasmic Reticulum Stress in the Adipocyte During Diabetes. Antioxid. Redox Signal.

[57] Piroli G.G., Manuel A.M., Walla M.D., Jepson M.J., Brock J.W.C., Rajesh M.P., Tanis R.M., Cotham W.E., Frizzell N. (2014). Identification of protein succination as a novel modification of tubulin. Biochem. J..

[58] Twelvetrees A.E. (2020). The lifecycle of the neuronal microtubule transport machinery. Semin. Cell Dev. Biol..

[59] Ferri A., Cozzolino M., Crosio C., Nencini M., Casciati A., Gralla E.D., Rotilio G., Valentine J.S., Carrì M.T. (2006). Familial ALS-superoxide dismutases associate with mitochondria and shift their redox potentials. Proc. Natl. Acad. Sci. USA.

[60] Magrì A., Belfiore R., Reina S., Tomasello M.F., Di Rosa M.C., Guarino F., Leggio L., De Pinto V., Messina A. (2016). Hexokinase I N-terminal based peptide prevents the VDAC1-SOD1 G93A interaction and re-establishes ALS cell viability. Sci. Rep..

[61] Aiello R., Messina A., Schiffler B., Benz R., Tasco G., Casadio R., De Pinto V. (2004). Functional characterization of a second porin isoform in Drosophila melanogaster–DmPorin2 forms voltage-independent cation-selective pores. J. Biol. Chem..

[62] Reina S., Magrì A., Lolicato M., Guarino F., Impellizzeri A., Maier E., Benz R., Ceccarelli M., De Pinto V., Messina A. (2013). Deletion of β-strands 9 and 10 converts VDAC1 voltage-dependence in an asymmetrical process. Biochim. Biophys. Acta Bioenerg..

[63] Guardiani C., Magrì A., Karachitos A., Di Rosa M.C., Reina S., Bodrenko I., Messina A., Kmita H., Ceccarelli M., De Pinto V. (2018). yVDAC2, the second mitochondrial porin isoform of Saccharomyces cerevisiae. Biochim. Biophys. Acta Bioenerg..

[64] Webb B., Sali A. (2016). Comparative Protein Structure Modeling Using MODELLER. Curr. Protoc..

