# Supplementary material for: Specific Post-Translational Modifications of VDAC3 in ALS-SOD1 Model Cells Identified by High-Resolution Mass Spectrometry
Source: Int J Mol Sci. 2022 Dec 13;23(24):15853. doi: 10.3390/ijms232415853 (PMC9784795; doi:10.3390/ijms232415853)
Supplement: Supplementary file 1 [file ijms-23-15853-s001.zip › ijms-1993097-supplementary.pdf]

Supplementary

Specific Post-translational Modifications of VDAC3 in ALS-SOD1 Model Cells Identified by High-Resolution Mass Spectrometry

Maria Gaetana Giovanna Pittalà [1], Simona Reina [2], Stefano Conti Nibali [2], Annamaria Cucina [1], Salvatore Antonio Maria Cubisino [2], Vincenzo Cunsolo [1], Giuseppe Federico Amodeo [3], Salvatore Foti [1], Vito De Pinto [2], Rosaria Saletti [1,*] and Angela Messina [4]

[1] Organic Mass Spectrometry Laboratory, Department of Chemical Sciences, University of Catania, Via S. Sofia 64, 95123 Catania, Italy
[2] Department of Biomedical and Biotechnological Sciences, University of Catania, Via S. Sofia 64, 95123 Catania, Italy
[3] Department of Neurology, Columbia University, New York, NY 10032, USA
[4] Department of Biological, Geological and Environmental Sciences, University of Catania, Via S. Sofia 64, 95123 Catania, Italy
* Correspondence: author: rsaletti@unict.it; Tel.: +39-095-738-5026 (R. S.).

SUPPLEMENTARY MATERIALS

FIGURES

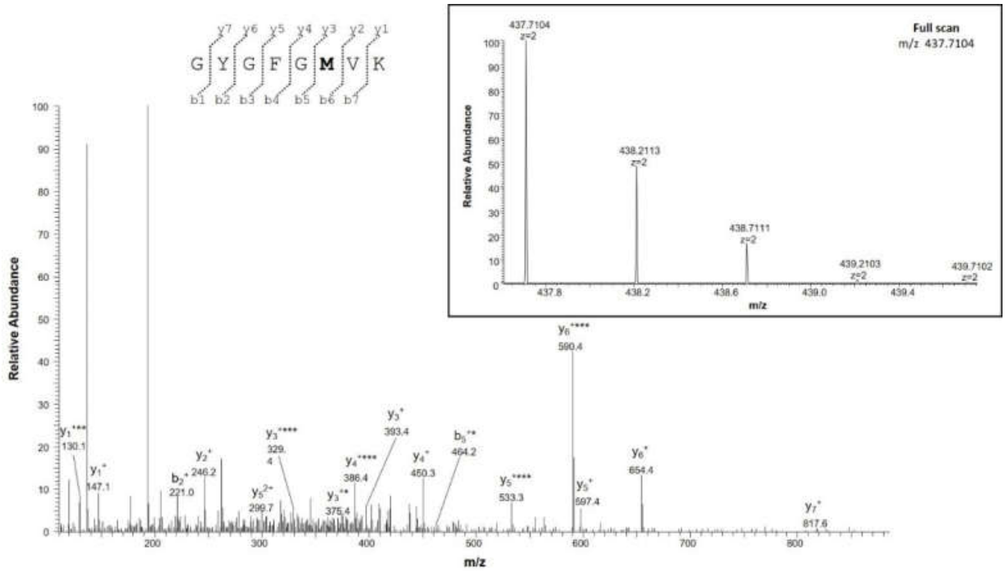

(A)

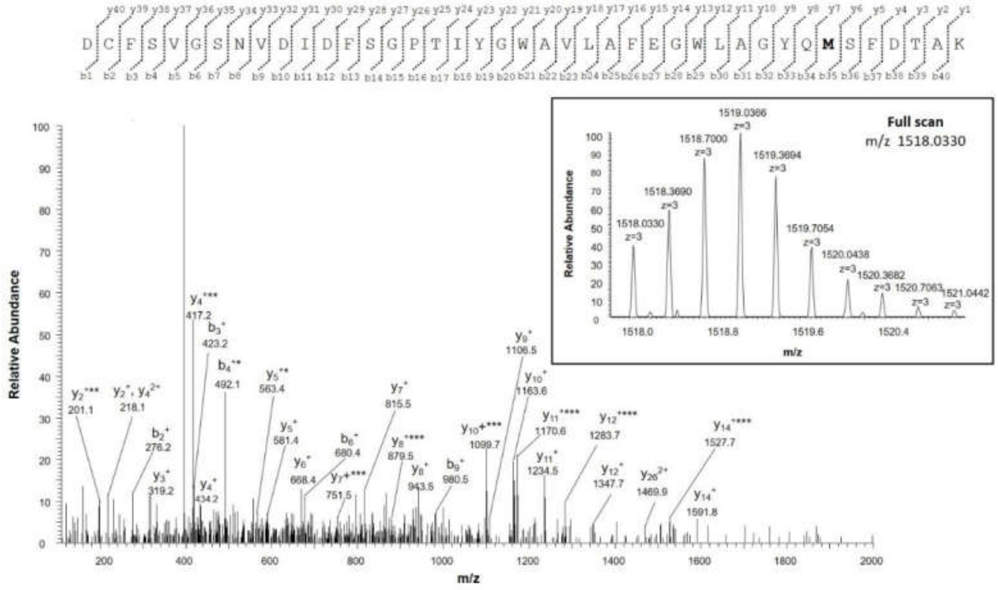

(B)

Supplementary Figure S1. A. MS/MS spectrum of the doubly charged molecular ion at m/z 437.7104 (calculated 437.7103) of the VDAC3 tryptic peptide from NSC34 cell line containing the methionine residue 26 in the oxidized form of methionine sulfoxide. The inset shows the full scan mass spectrum of molecular ion. Fragment ions originated from the neutral loss of H_2O are indicated by an asterisk. Fragment ion originated from the neutral loss of NH_3 is indicated by two asterisks Fragment ions originated from the neutral loss of methanesulfenic acid (CH_3SOH, 64 Da) are indicated by three asterisks. **B.** MS/MS spectrum of the triply charged molecular ion at m/z 1518.0330 (calculated 1518.0315) of the VDAC3 tryptic peptide from NSC34 cell line containing the methionine residue 155 in the oxidized form of methionine sulfoxide. The inset shows the full scan mass spectrum of molecular ion. Fragment ions originated from the neutral loss of H_2O are indicated by an asterisk. Fragment ions originated from the neutral loss of NH_3 are indicated by two asterisks Fragment ions originated from the neutral loss of methanesulfenic acid (CH_3SOH, 64 Da) are indicated by three asterisks.

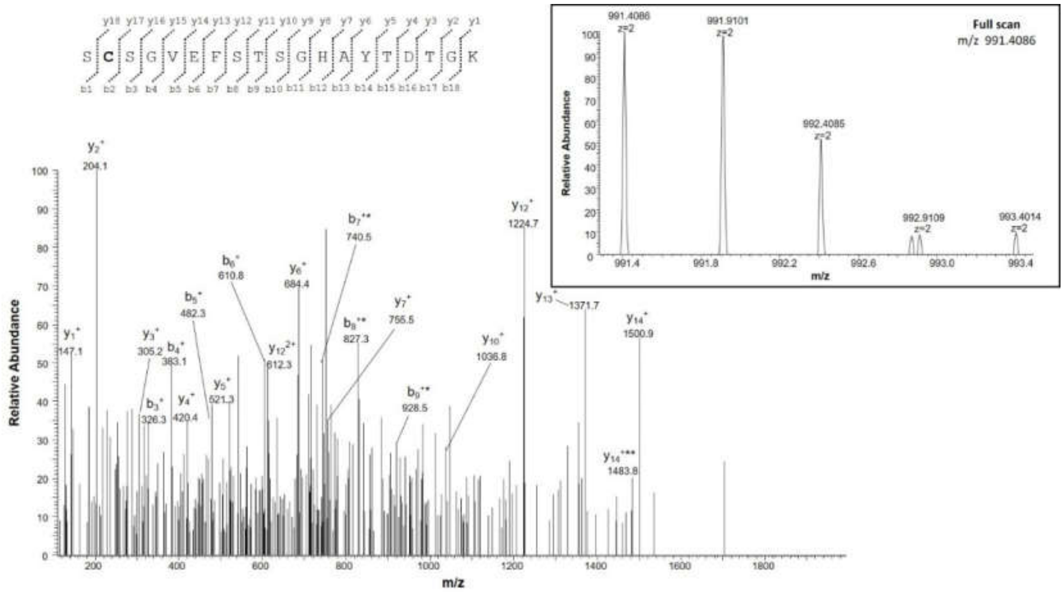
S C S G V E F S T S G H A Y T D T G K
y18 y17 y16 y15 y14 y13 y12 y11 y10 y9 y8 y7 y6 y5 y4 y3 y2 y1
b1 b2 b3 b4 b5 b6 b7 b8 b9 b10 b11 b12 b13 b14 b15 b16 b17 b18
Relative Abundance
m/z
y2+ 204.1
y1+ 147.1
y3+ 305.2
b3+ 326.3
b4+ 383.1
y4+ 420.4
b5+ 482.3
y5+ 521.3
b6+ 610.8
y12 2+ 612.3
y6+ 684.4
b7+* 740.5
y7+ 755.5
b8+* 827.3
b9+* 928.5
y10+ 1036.8
y12+ 1224.7
y13+ 1371.7
y14+ 1500.9
y14+** 1483.8
Full scan
m/z 991.4086
991.4086 z=2
991.9101 z=2
992.4085 z=2
992.9109 z=2
993.4014 z=2

(A)

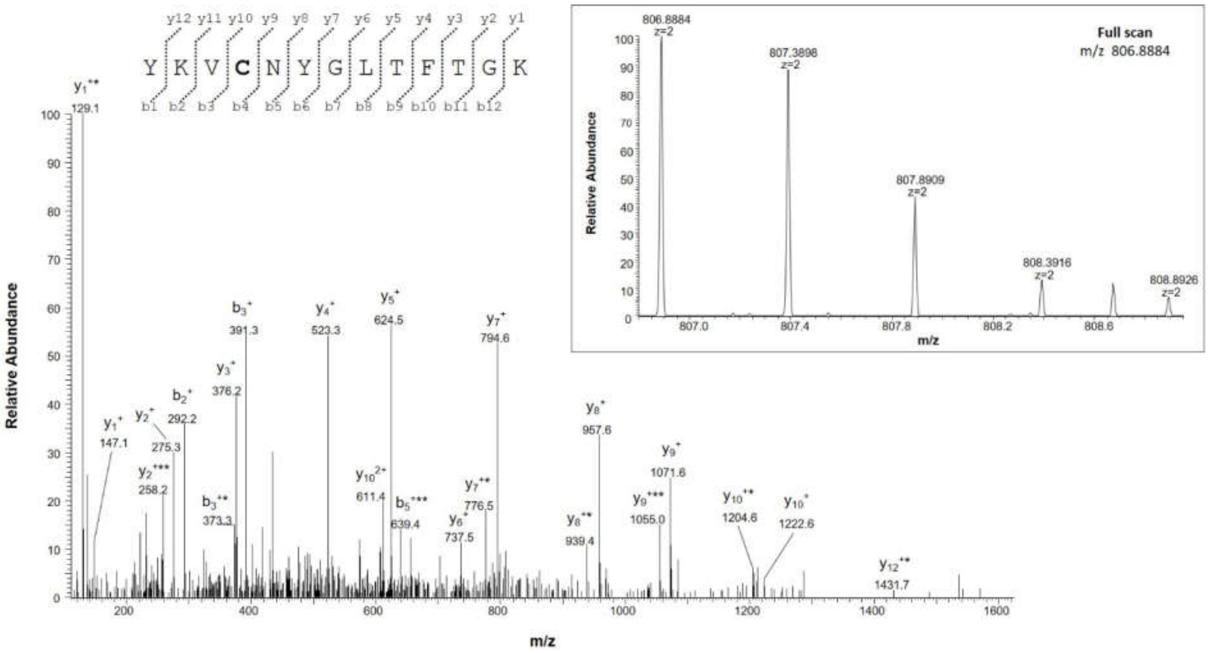
Y K V C N Y G L T F T G K
y12 y11 y10 y9 y8 y7 y6 y5 y4 y3 y2 y1
b1 b2 b3 b4 b5 b6 b7 b8 b9 b10 b11 b12
Relative Abundance
m/z
y1+* 129.1
y1+ 147.1
y2+ 275.3
y2+** 258.2
b2+ 292.2
b3+* 373.3
y3+ 376.2
b3+ 391.3
y4+ 523.3
y5+ 624.5
y10 2+ 611.4
b5+** 639.4
y6+ 737.5
y7+* 776.5
y7+ 794.6
y8+ 957.6
y8+* 939.4
y9+** 1055.0
y9+ 1071.6
y10+* 1204.6
y10+ 1222.6
y12+* 1431.7
Full scan
m/z 806.8884
806.8884 z=2
807.3898 z=2
807.8909 z=2
808.3916 z=2
808.8926 z=2

(B)

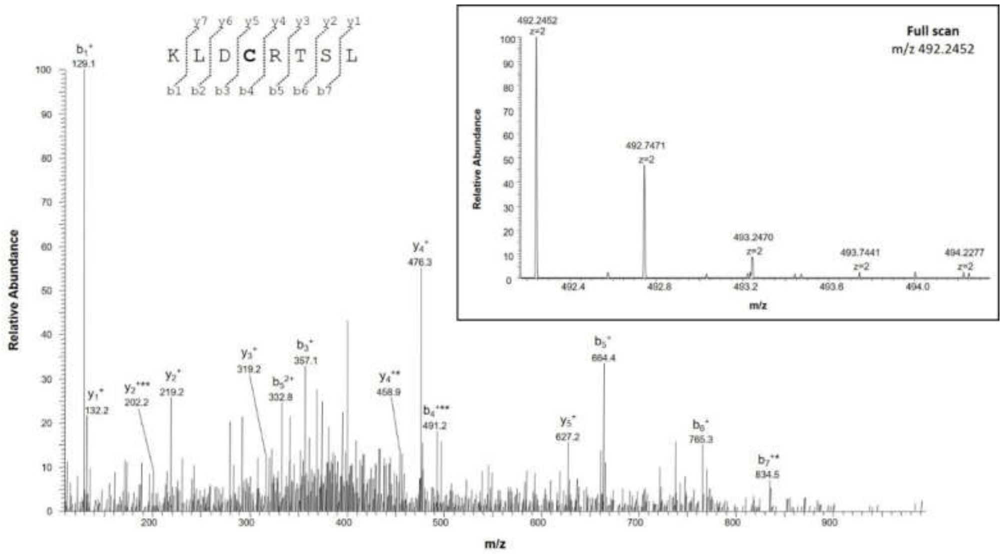

(C)

Supplementary Figure S2. A. MS/MS spectrum of the doubly charged molecular ion at m/z 991.4086 (calculated 991.4079) of the VDAC3 tryptic peptide from NSC34 cell line containing cysteine residue 36 in the form of sulfonic acid. The inset shows the full scan mass spectrum of molecular ion. Fragment ions originated from the neutral loss of H_2O are indicated by an asterisk. Fragment ion originated from the neutral loss of NH_3 is indicated by two asterisks. **B.** MS/MS spectrum of the doubly charged molecular ion at m/z 806.8884 (calculated 806.8877) of the VDAC3 tryptic peptide from NSC34 cell line containing cysteine residue 65 in the form of sulfonic acid. The inset shows the full scan mass spectrum of molecular ion. Fragment ions originated from the neutral loss of H_2O are indicated by an asterisk. Fragment ions originated from the neutral loss of NH_3 are indicated by two asterisks. **C.** MS/MS spectrum of the doubly charged molecular ion at m/z 492.2452 (calculated 492.2455) of the VDAC3 chymotryptic peptide from NSC34 cell line containing cysteine residue 229 in the form of sulfonic acid. The inset shows the full scan mass spectrum of molecular ion. Fragment ions originated from the neutral loss of H_2O are indicated by an asterisk. Fragment ions originated from the neutral loss of NH_3 are indicated by two asterisks.

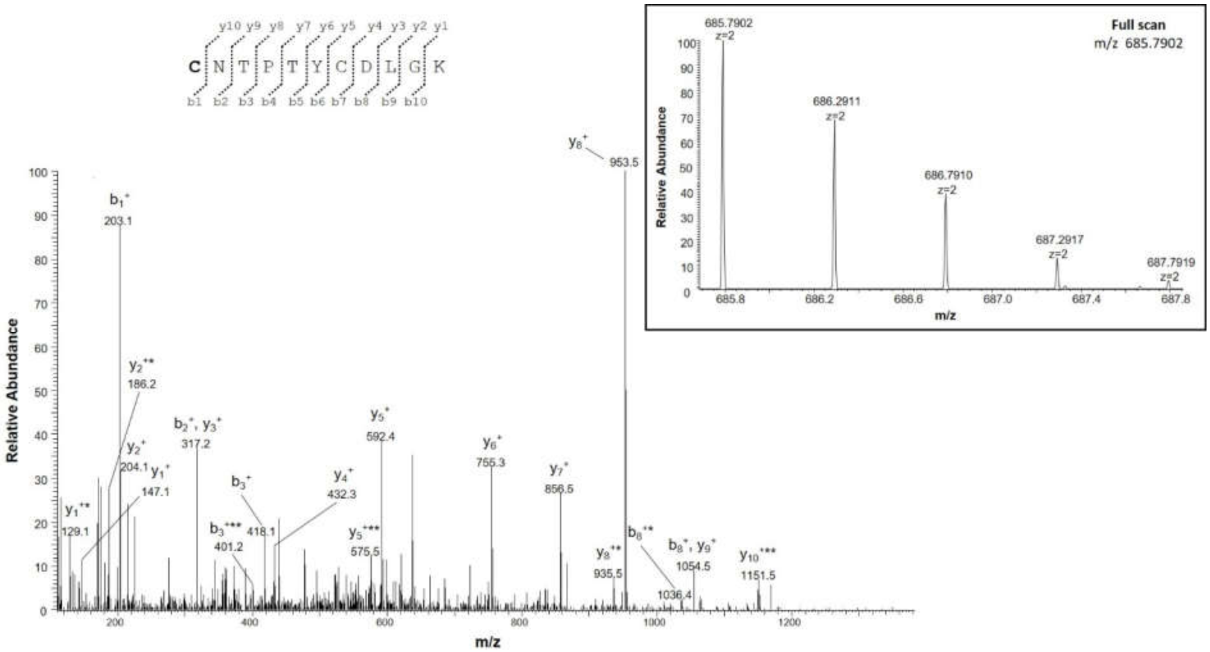

Supplementary Figure S3. MS/MS spectrum of the doubly charged molecular ion at m/z 685.7902 (calculated 685.7897) of the N-terminal acetylated tryptic peptide of VDAC3 from NSC34 cell line with Cys[2] and Cys[8] in the carboxyamidomethylated form. The inset shows the full scan mass spectrum of molecular ion. Fragment ions originated from the neutral loss of H_2O are indicated by an asterisk. Fragment ions originated from the neutral loss of NH_3 are indicated by two asterisks.

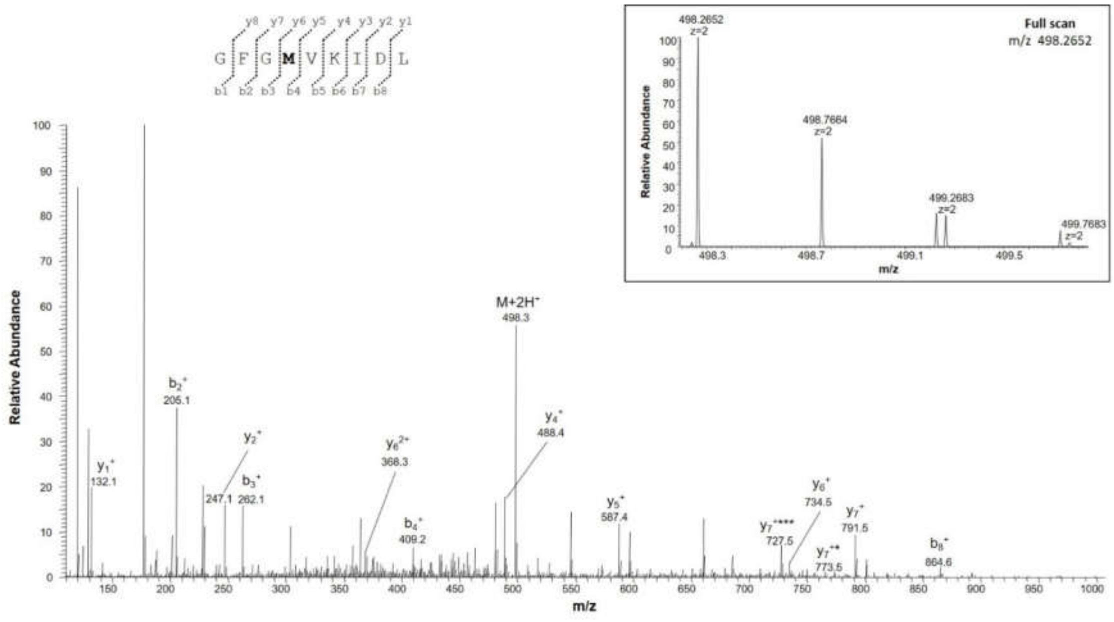

(A)

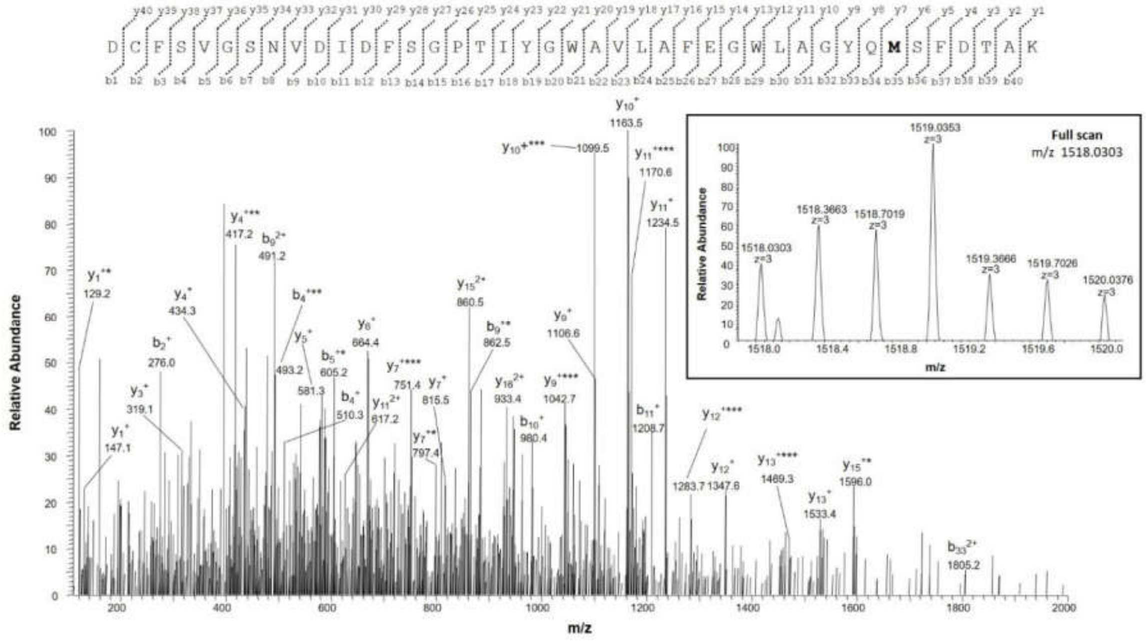

(B)

Supplementary Figure S4. A. MS/MS spectrum of the doubly charged molecular ion at m/z 498.2652 (calculated 437.7103) of the VDAC3 chymotryptic peptide from NSC34-SOD1WT cell line containing the methionine residue 26 in the oxidized form of methionine sulfoxide. The inset shows the full scan mass spectrum of molecular ion. Fragment ion originated from the neutral loss of H_2O is indicated by an asterisk. Fragment ion originated from the neutral loss of methanesulfenic acid (CH_3SOH, 64 Da) is indicated by three asterisks. **B.** MS/MS spectrum of the triply charged molecular ion at m/z 1518.0303 (calculated 1518.0315) of the VDAC3 tryptic peptide from NSC34-SOD1WT cell line containing the methionine residue 155 in the oxidized form of methionine sulfoxide. The inset shows the full scan mass spectrum of molecular ion. Fragment ions originated from the neutral loss of H_2O are indicated by an asterisk. Fragment ions originated from the neutral loss of NH_3 are indicated by two asterisks. Fragment ions originated from the neutral loss of methanesulfenic acid (CH_3SOH, 64 Da) are indicated by three asterisks.

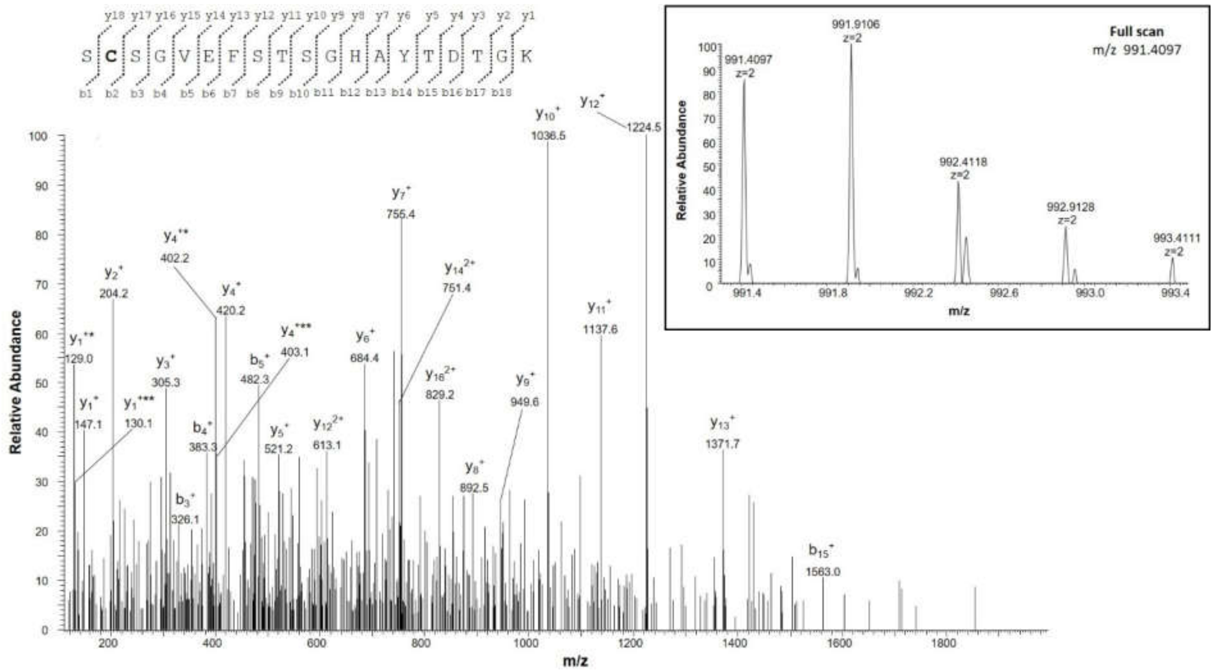
S C S G V E F S T S G H A Y T D T G K
Relative Abundance
m/z
Full scan
m/z 991.4097
991.4097 z=2
991.9106 z=2
992.4118 z=2
992.9128 z=2
993.4111 z=2
y10+ 1036.5
y12+ 1224.5
y7+ 755.4
y4++ 402.2
y2+ 204.2
y4+ 420.2
y14 2+ 751.4
y1++ 129.0
y4+++ 403.1
y6+ 684.4
y11+ 1137.6
y3+ 305.3
b5+ 482.3
y16 2+ 829.2
y9+ 949.6
y1+ 147.1
y1+++ 130.1
b4+ 383.3
y5+ 521.2
y12 2+ 613.1
y13+ 1371.7
y8+ 892.5
b3+ 326.1
b15+ 1563.0

(A)

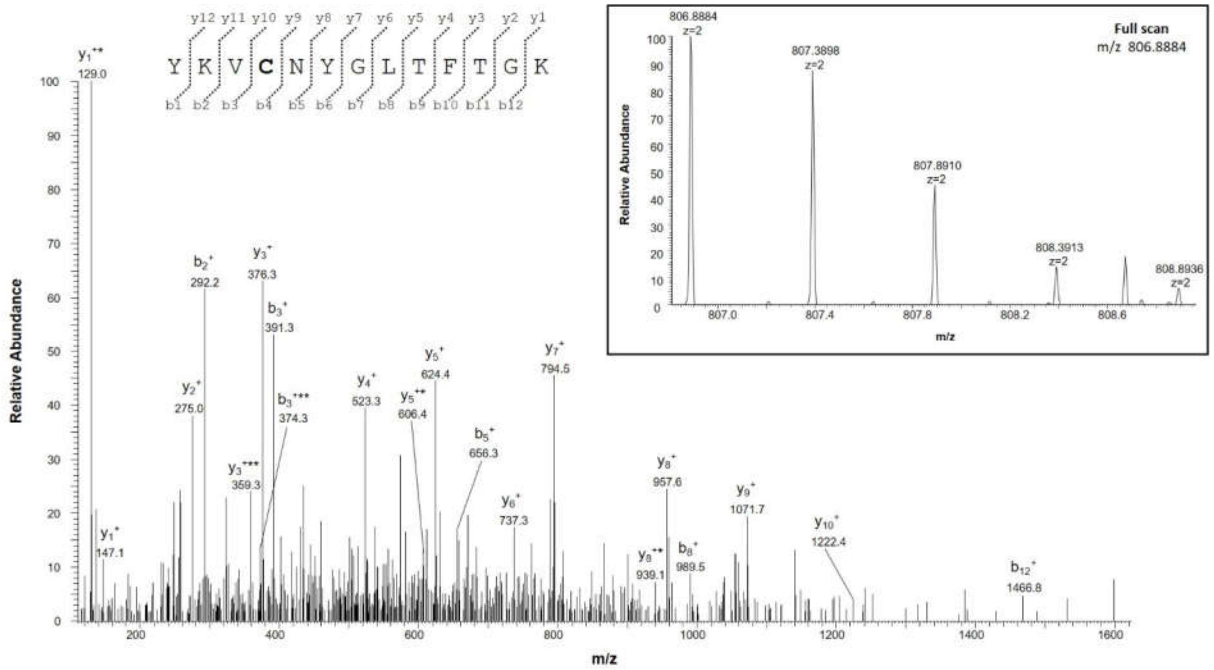
Y K V C N Y G L T F T G K
Relative Abundance
m/z
Full scan
m/z 806.8884
806.8884 z=2
807.3898 z=2
807.8910 z=2
808.3913 z=2
808.8936 z=2
y1++ 129.0
b2+ 292.2
y3+ 376.3
b3+ 391.3
y2+ 275.0
y5+ 624.4
y7+ 794.5
y4+ 523.3
y5++ 606.4
b3+++ 374.3
b5+ 656.3
y3+++ 359.3
y8+ 957.6
y9+ 1071.7
y6+ 737.3
y1+ 147.1
y8++ 939.1
b8+ 989.5
y10+ 1222.4
b12+ 1466.8

(B)

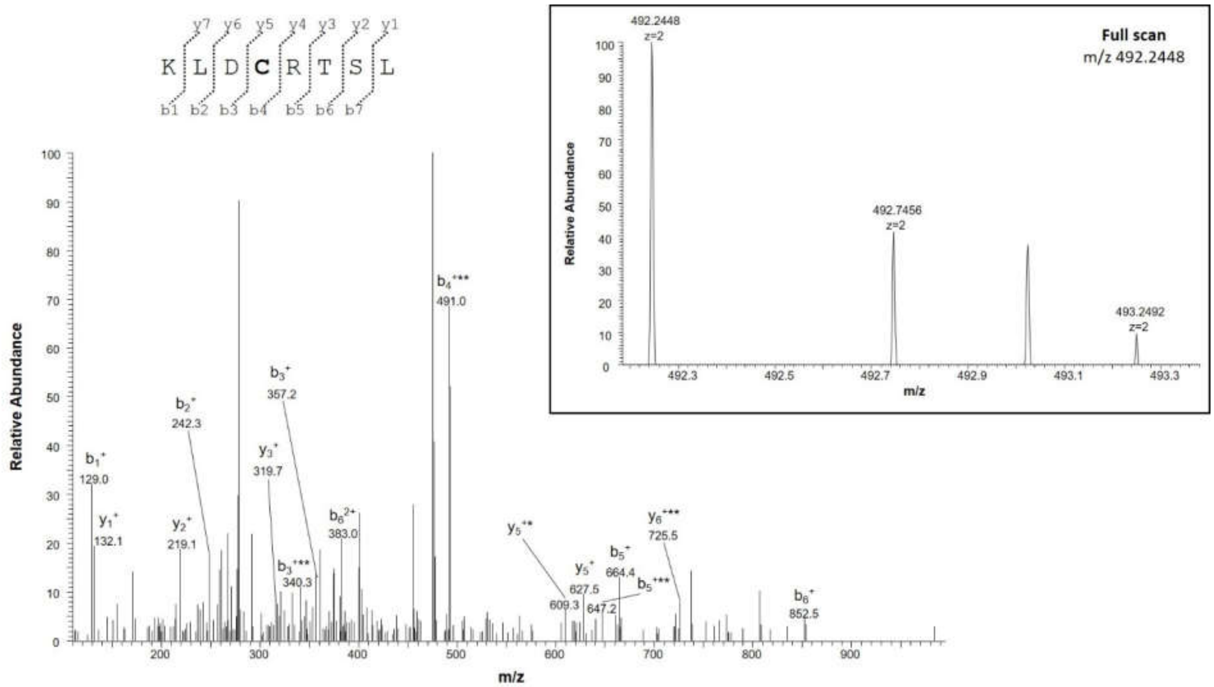

(C)

Supplementary Figure S5. A. MS/MS spectrum of the doubly charged molecular ion at m/z 991.4097 (calculated 991.4079) of the VDAC3 tryptic peptide from NSC34-SOD1WT cell line containing cysteine residue 36 in the form of sulfonic acid. The inset shows the full scan mass spectrum of molecular ion. Fragment ions originated from the neutral loss of H_2O are indicated by an asterisk. Fragment ion originated from the neutral loss of NH_3 is indicated by two asterisks. **B.** MS/MS spectrum of the doubly charged molecular ion at m/z 806.8884 (calculated 806.8877) of the VDAC3 tryptic peptide from NSC34-SOD1WT cell line containing cysteine residue 65 in the form of sulfonic acid. The inset shows the full scan mass spectrum of molecular ion. Fragment ions originated from the neutral loss of H_2O are indicated by an asterisk. Fragment ions originated from the neutral loss of NH_3 are indicated by two asterisks. **C.** MS/MS spectrum of the doubly charged molecular ion at m/z 492.2448 (calculated 492.2455) of the VDAC3 chymotryptic peptide from NSC34-SOD1WT cell line containing cysteine residue 229 in the form of sulfonic acid. The inset shows the full scan mass spectrum of molecular ion. Fragment ion originated from the neutral loss of H_2O is indicated by an asterisk. Fragment ions originated from the neutral loss of NH_3 are indicated by two asterisks.

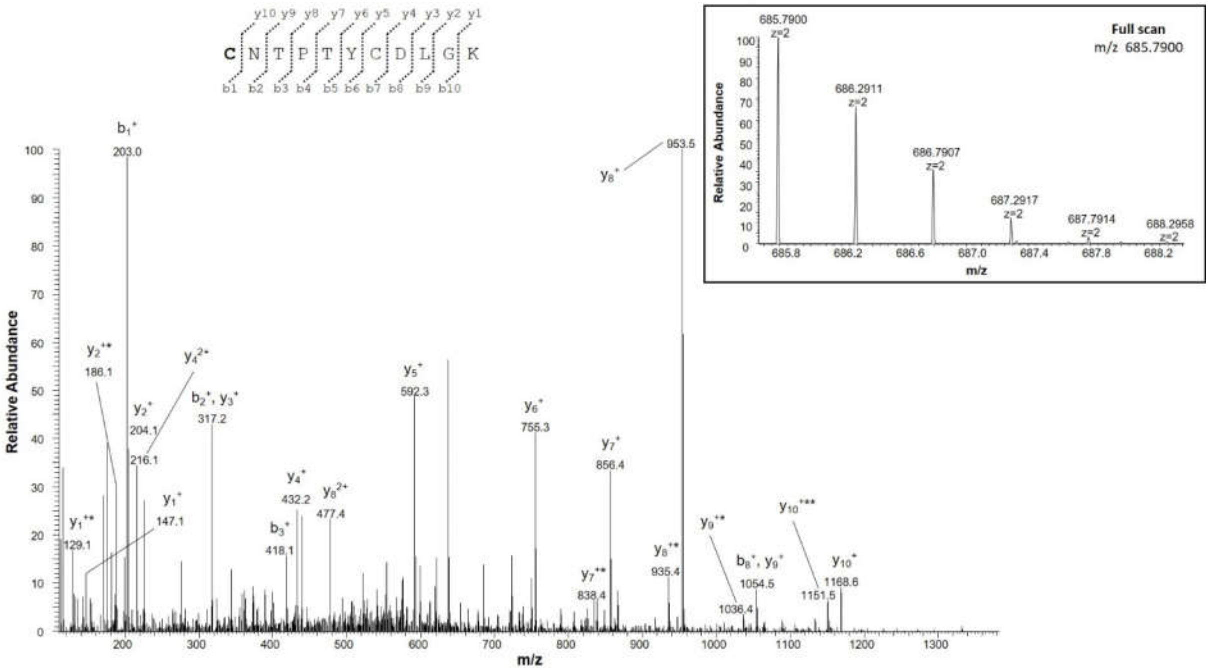

Supplementary Figure S6. MS/MS spectrum of the doubly charged molecular ion at m/z 685.7902 (calculated 685.7897) of the N-terminal acetylated tryptic peptide of VDAC3 from NSC34 SOD1WT cell line with Cys^2 and Cys^8 in the carboxyamidomethylated form. The inset shows the full scan mass spectrum of molecular ion. Fragment ions originated from the neutral loss of H_2O are indicated by an asterisk. Fragment ions originated from the neutral loss of NH_3 are indicated by two asterisks.

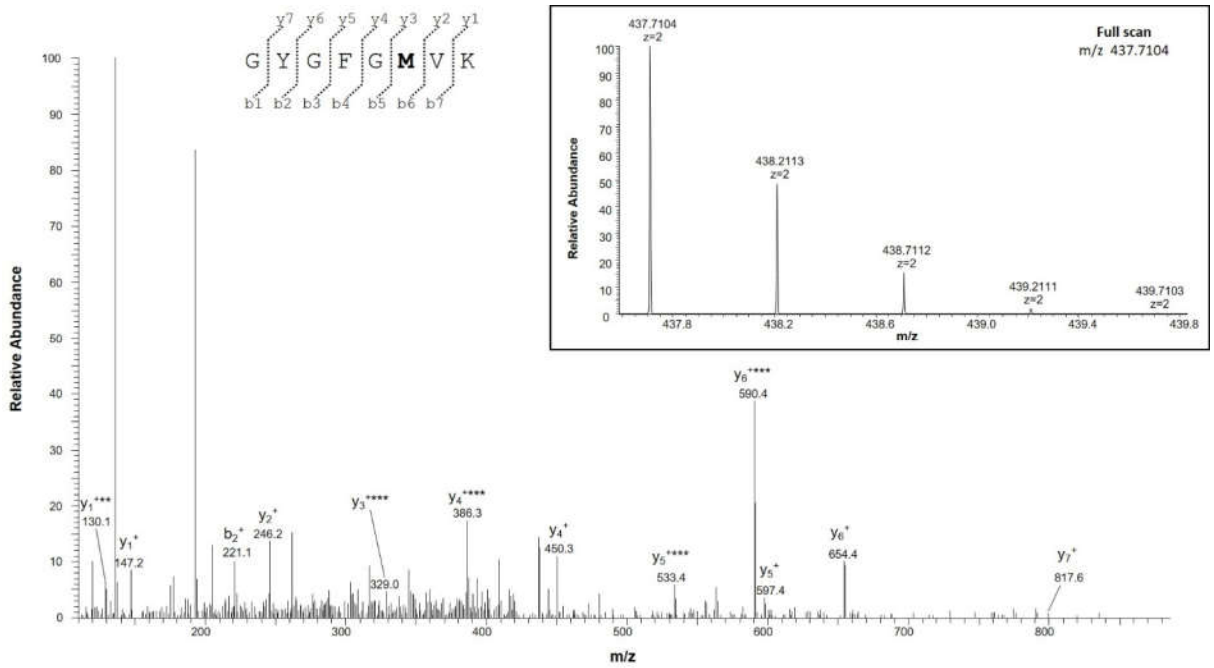

(A)

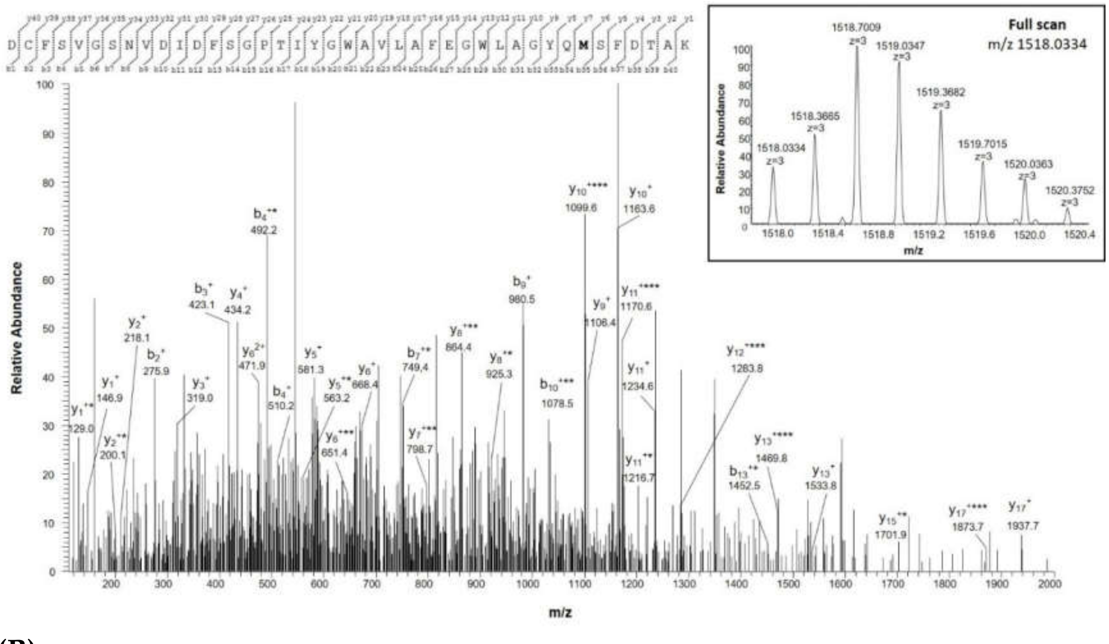

(B)

Supplementary Figure S7. A. MS/MS spectrum of the doubly charged molecular ion at m/z 437.7104 (calculated 437.7103) of the VDAC3 tryptic peptide from NSC34-SOD1G93A cell line containing the methionine residue 26 in the oxidized form of methionine sulfoxide. The inset shows the full scan mass spectrum of molecular ion. Fragment ion originated from the neutral loss of NH_3 is indicated by two asterisks. Fragment ions originated from the neutral loss of methanesulfenic acid (CH_3SOH, 64 Da) are indicated by three asterisks. **B.** MS/MS spectrum of the triply charged molecular ion at m/z 1518.0334 (calculated 1518.0315) of the VDAC3 tryptic peptide from NSC34-SOD1G93A cell line containing the methionine residue 155 in the oxidized form of methionine sulfoxide. The inset shows the full scan mass spectrum of molecular ion. Fragment ions originated from the neutral loss of H_2O are indicated by an asterisk. Fragment ions originated from the neutral loss of NH_3 are indicated by two asterisks. Fragment ions originated from the neutral loss of methanesulfenic acid (CH_3SOH, 64 Da) are indicated by three asterisks.

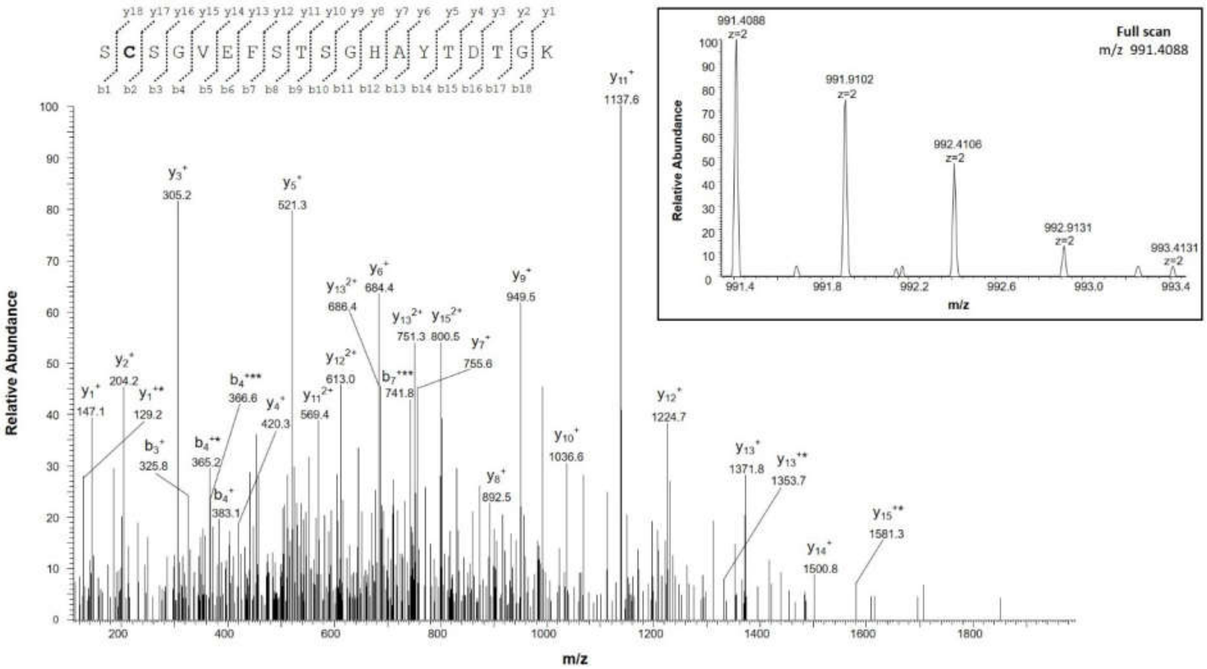

y18 y17 y16 y15 y14 y13 y12 y11 y10 y9 y8 y7 y6 y5 y4 y3 y2 y1
S C S G V E F S T S G H A Y T D T G K
b1 b2 b3 b4 b5 b6 b7 b8 b9 b10 b11 b12 b13 b14 b15 b16 b17 b18
Relative Abundance
m/z
y11+ 1137.6
y3+ 305.2
y5+ 521.3
y6+ 684.4
y13 2+ 686.4
y13 2+ 751.3
y15 2+ 800.5
y7+ 755.6
y9+ 949.5
y12 2+ 613.0
b7+** 741.8
y2+ 204.2
y1+ 147.1
y1+* 129.2
b4+** 366.6
y4+ 420.3
y11 2+ 569.4
b3+ 325.8
b4+* 365.2
b4+ 383.1
y12+ 1224.7
y10+ 1036.6
y8+ 892.5
y13+ 1371.8
y13+* 1353.7
y15+* 1581.3
y14+ 1500.8
Full scan
m/z 991.4088
991.4088 z=2
991.9102 z=2
992.4106 z=2
992.9131 z=2
993.4131 z=2

(A)

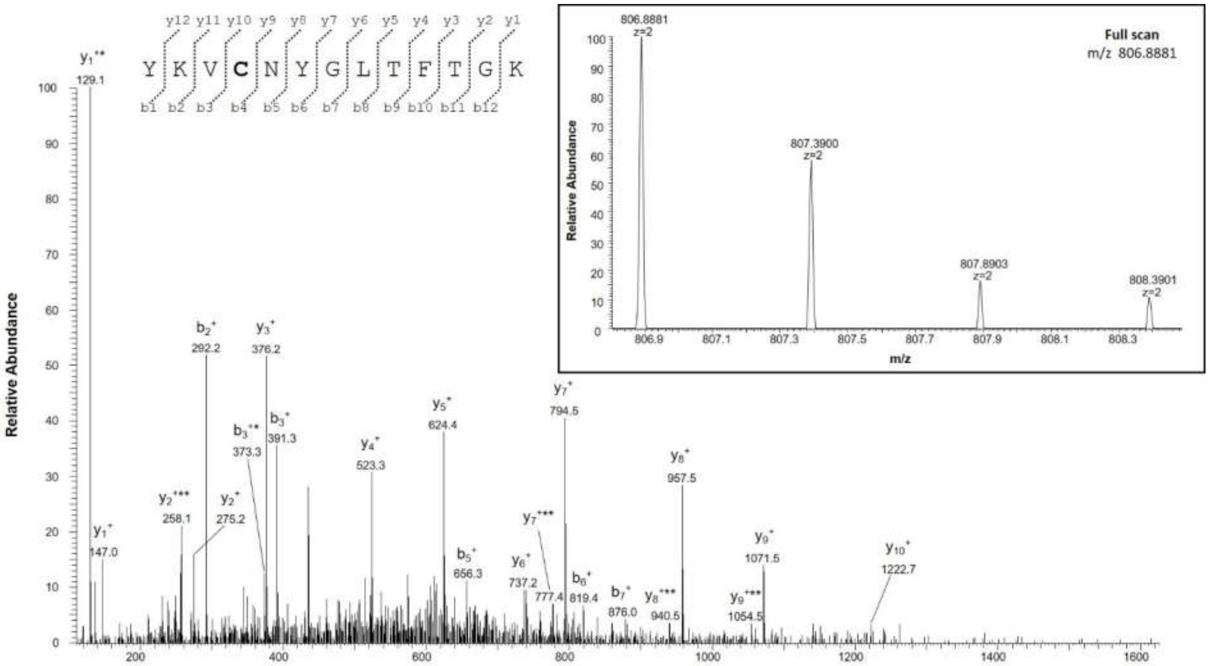

y12 y11 y10 y9 y8 y7 y6 y5 y4 y3 y2 y1
Y K V C N Y G L T F T G K
b1 b2 b3 b4 b5 b6 b7 b8 b9 b10 b11 b12
Relative Abundance
m/z
y1+* 129.1
y1+ 147.0
b2+ 292.2
y3+ 376.2
b3+* 373.3
b3+ 391.3
y2+** 258.1
y2+ 275.2
y4+ 523.3
y5+ 624.4
b5+ 656.3
y7+ 794.5
y7+** 777.4
y6+ 737.2
b6+ 819.4
b7+ 876.0
y8+** 940.5
y8+ 957.5
y9+ 1071.5
y9+** 1054.5
y10+ 1222.7
Full scan
m/z 806.8881
806.8881 z=2
807.3900 z=2
807.8903 z=2
808.3901 z=2

(B)

K[226]L D **C** R T S L[233]

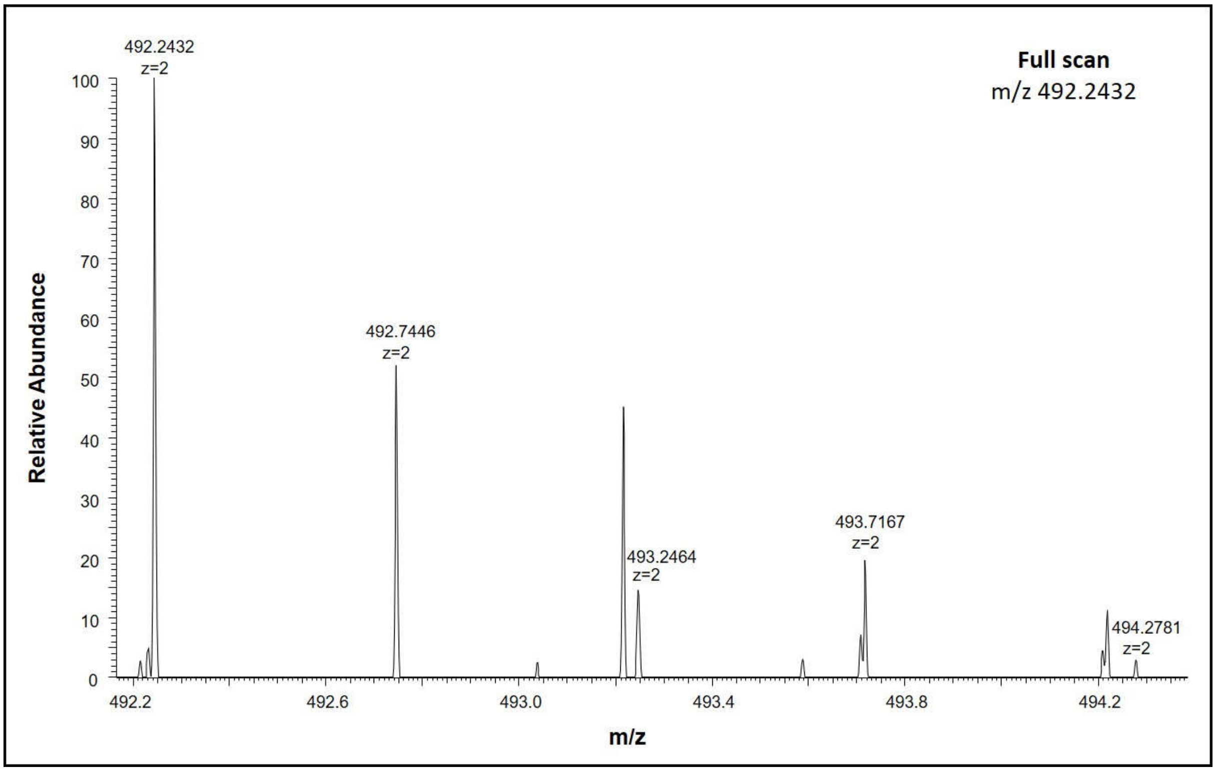

(C)

Supplementary Figure S8. A. MS/MS spectrum of the doubly charged molecular ion at m/z 991.4088 (calculated 991.4079) of the VDAC3 tryptic peptide from NSC34-SOD1G93A cell line containing cysteine residue 36 in the form of sulfonic acid. The inset shows the full scan mass spectrum of molecular ion. Fragment ions originated from the neutral loss of H_2O are indicated by an asterisk. Fragment ions originated from the neutral loss of NH_3 are indicated by two asterisks. **B.** MS/MS spectrum of the doubly charged molecular ion at m/z 806.8881 (calculated 806.8877) of the VDAC3 tryptic peptide from NSC34-SOD1G93A cell line containing cysteine residue 65 in the form of sulfonic acid. The inset shows the full scan mass spectrum of molecular ion. Fragment ion originated from the neutral loss of H_2O is indicated by an asterisk. Fragment ions originated from the neutral loss of NH_3 are indicated by two asterisks. **C.** Full scan mass spectrum of the doubly charged molecular ion at m/z 492.2432 (calculated 492.2455) of the VDAC3 chymotryptic peptide from NSC34-SOD1G93A cell line containing cysteine residue 229 in the form of sulfonic acid.

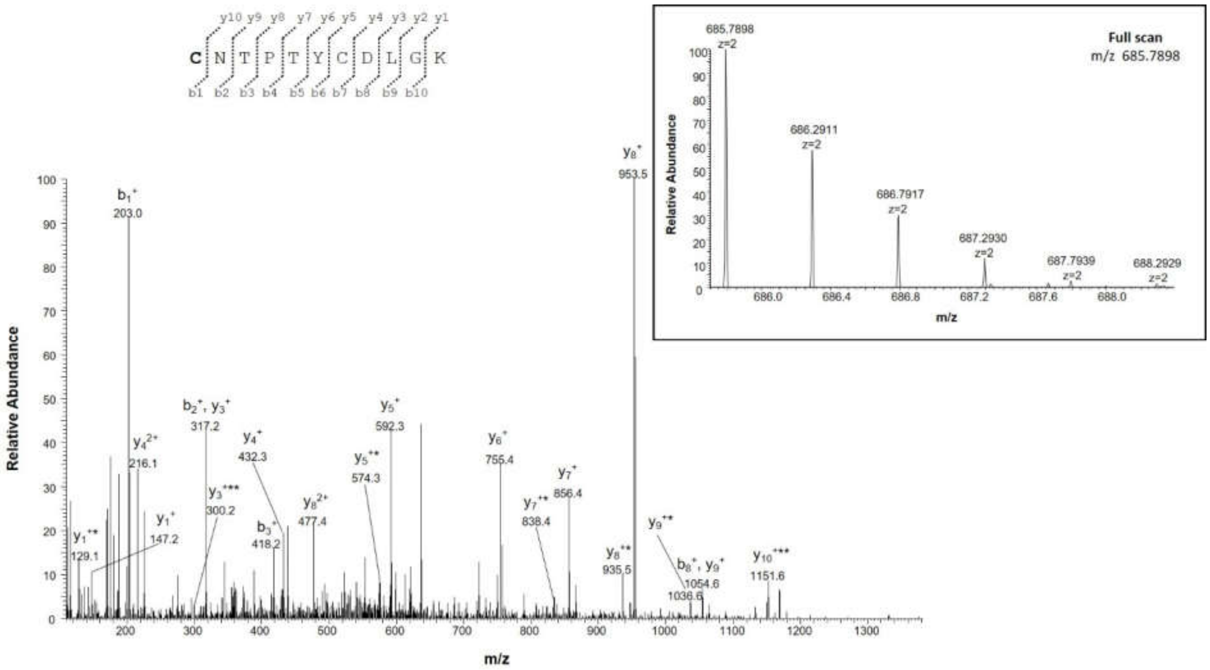

Supplementary Figure S9. MS/MS spectrum of the doubly charged molecular ion at m/z 685.7898 (calculated 685.7897) of the N-terminal acetylated tryptic peptide of VDAC3 from NSC34-SOD1G93A cell line with Cys[2] and Cys[8] in the carboxyamidomethylated form. The inset shows the full scan mass spectrum of molecular ion. Fragment ions originated from the neutral loss of H_2O are indicated by an asterisk. Fragment ions originated from the neutral loss of NH_3 are indicated by two asterisks.

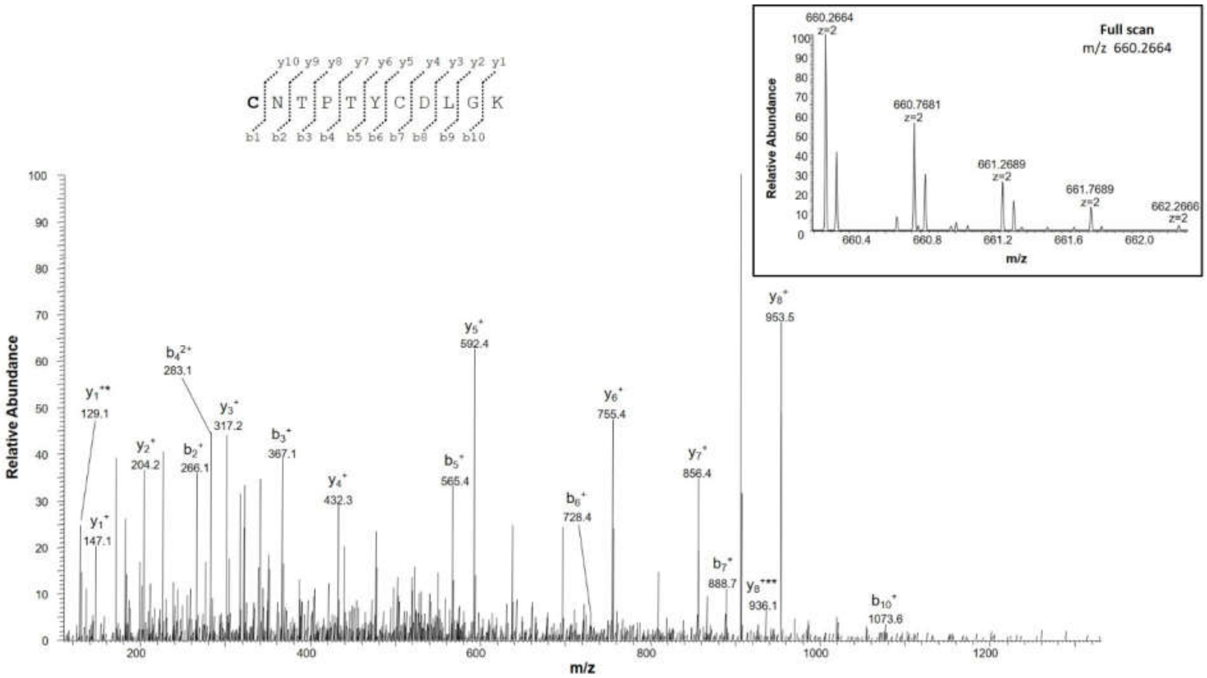

Supplementary Figure S10. MS/MS spectrum of the doubly charged molecular ion at m/z 660.2664 (calculated 660.2663) of the N-terminal tryptic peptide of VDAC3 from the cell line NSC34-SOD1G93A with cysteine residue 2 in the form of sulfonic acid and cysteine residue 8 in the carboxyamidomethylated form. The inset shows the full scan mass spectrum of the molecular ion. The fragment ion originating from the neutral loss of H_2O is indicated by an asterisk. The fragment ion originating from the neutral loss of NH_3 is indicated by two asterisks.

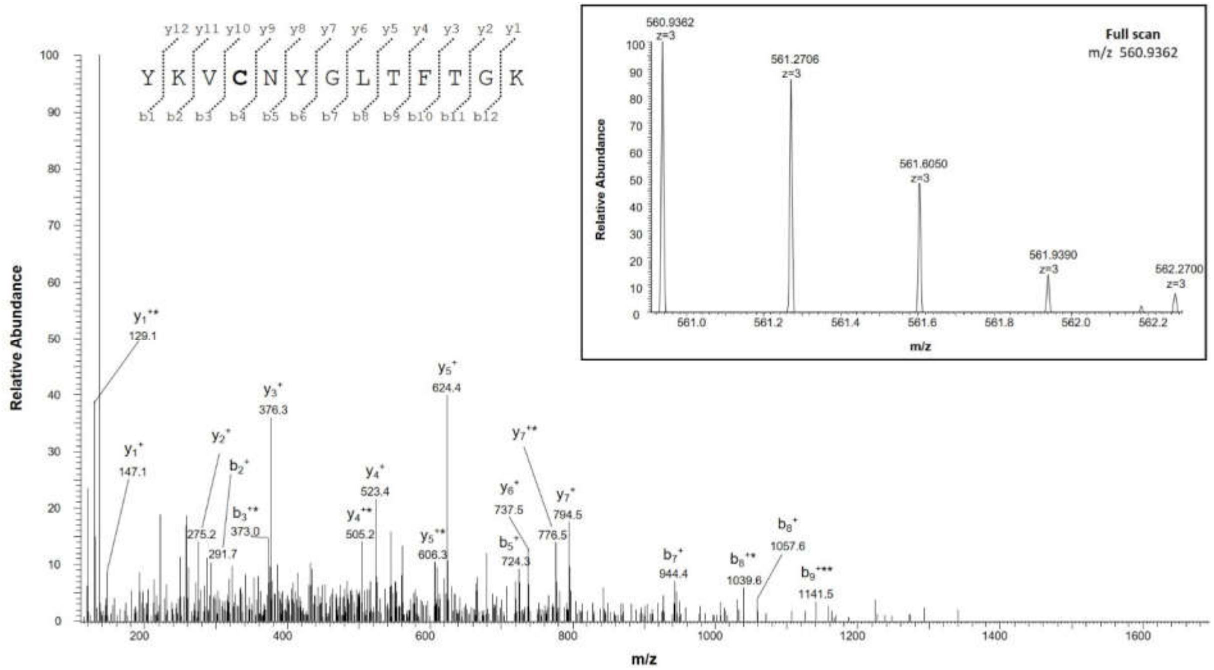
y12 y11 y10 y9 y8 y7 y6 y5 y4 y3 y2 y1
Y K V C N Y G L T F T G K
b1 b2 b3 b4 b5 b6 b7 b8 b9 b10 b11 b12
Relative Abundance
m/z
y1++ 129.1
y1+ 147.1
y2+ 275.2
b2+ 291.7
b3++ 373.0
y3+ 376.3
y4++ 505.2
y4+ 523.4
y5++ 606.3
y5+ 624.4
b5+ 724.3
y6+ 737.5
y7++ 776.5
y7+ 794.5
b7+ 944.4
b8++ 1039.6
b8+ 1057.6
b9+++ 1141.5
Full scan
m/z 560.9362
560.9362 z=3
561.2706 z=3
561.6050 z=3
561.9390 z=3
562.2700 z=3

(A)

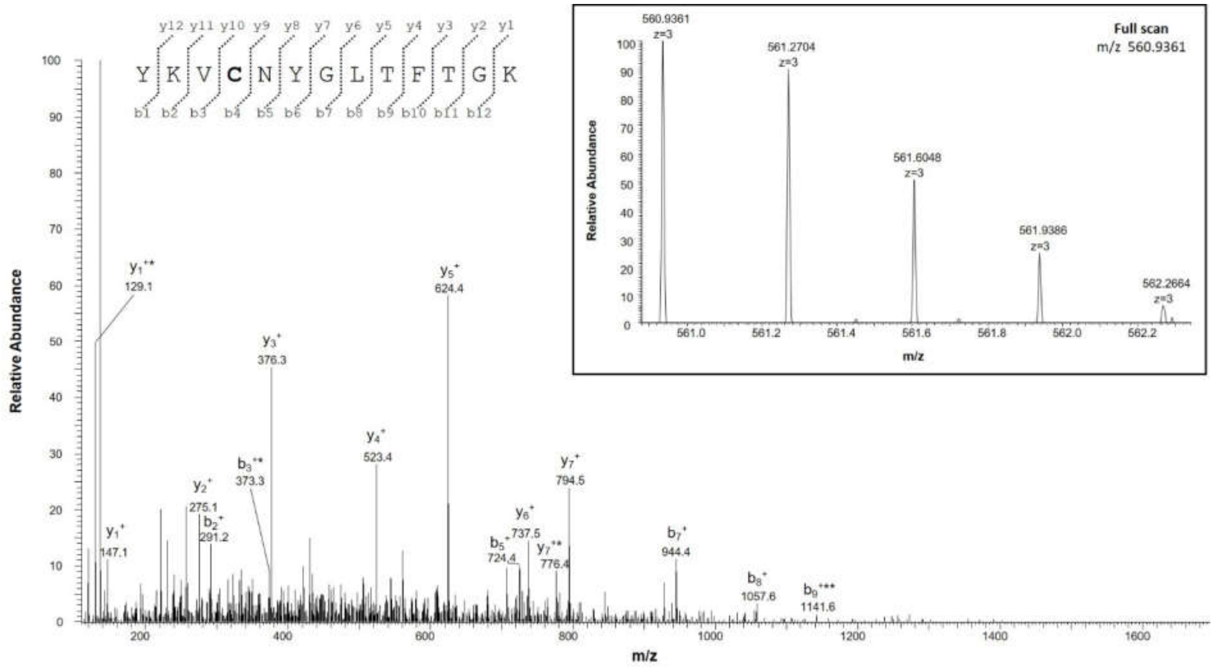
y12 y11 y10 y9 y8 y7 y6 y5 y4 y3 y2 y1
Y K V C N Y G L T F T G K
b1 b2 b3 b4 b5 b6 b7 b8 b9 b10 b11 b12
Relative Abundance
m/z
y1++ 129.1
y1+ 147.1
y2+ 275.1
b2+ 291.2
b3++ 373.3
y3+ 376.3
y4+ 523.4
y5+ 624.4
b5+ 724.4
y6+ 737.5
y7++ 776.4
y7+ 794.5
b7+ 944.4
b8+ 1057.6
b9+++ 1141.6
Full scan
m/z 560.9361
560.9361 z=3
561.2704 z=3
561.6048 z=3
561.9386 z=3
562.2664 z=3

(B)

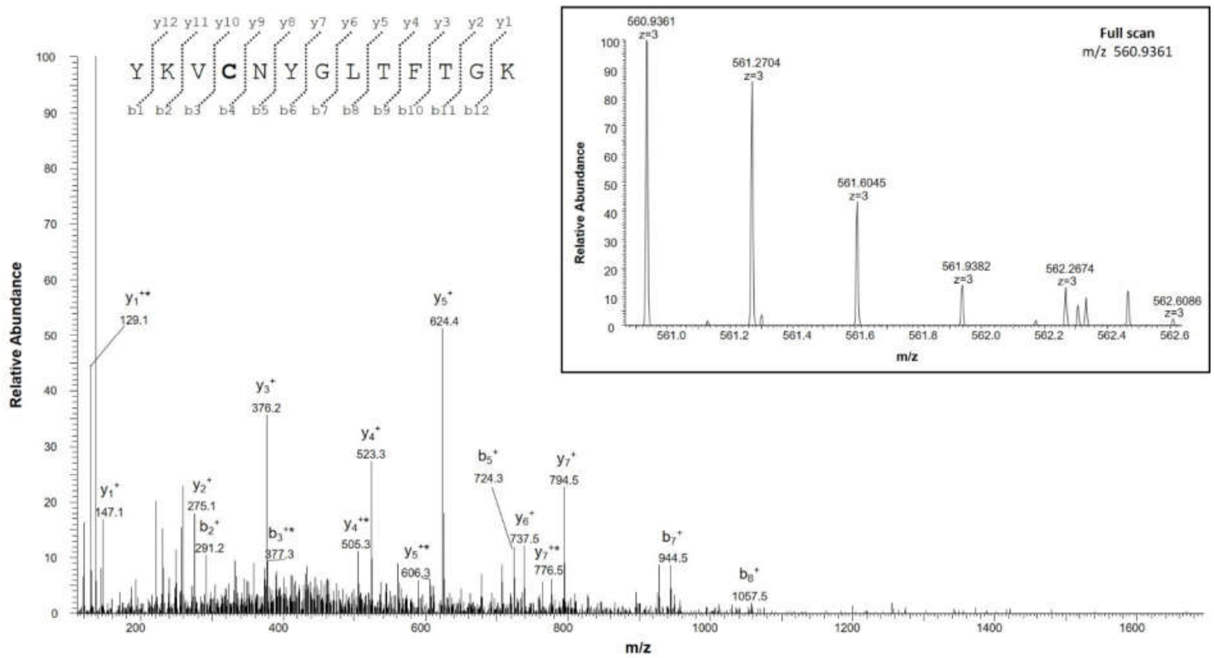

(C)

Supplementary Figure S11. A. MS/MS spectrum of the triply charged molecular ion at m/z 560.9362 (calculated 560.9367) of the VDAC3 tryptic peptide from NSC34 cell line containing cysteine residue 65 in the succinated form. The inset shows the full scan mass spectrum of molecular ion. Fragment ions originated from the neutral loss of H_2O are indicated by an asterisk. Fragment ion originated from the neutral loss of NH_3 is indicated by two asterisks. **B.** MS/MS spectrum of the triply charged molecular ion at m/z 560.9361 (calculated 560.9367) of the VDAC3 tryptic peptide from NSC34-SOD1WT cell line containing cysteine residue 65 in the succinated form. The inset shows the full scan mass spectrum of molecular ion. Fragment ions originated from the neutral loss of H_2O are indicated by an asterisk. Fragment ion originated from the neutral loss of NH_3 is indicated by two asterisks. **C.** MS/MS spectrum of the triply charged molecular ion at m/z 560.9361 (calculated 560.9367) of the VDAC3 tryptic peptide from NSC34SOD1G93A cell line containing cysteine residue 65 in the succinated form. The inset shows the full scan mass spectrum of molecular ion. Fragment ions originated from the neutral loss of H_2O are indicated by an asterisk.

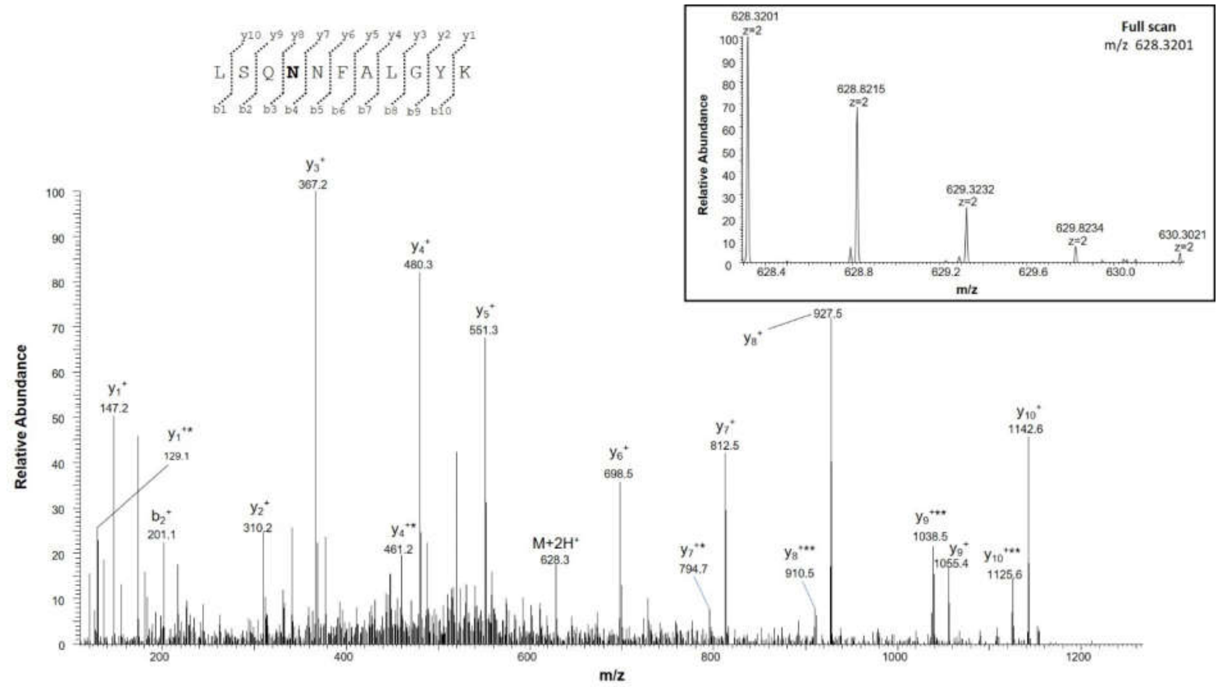

L S Q N N F A L G Y K
Full scan
m/z 628.3201
Relative Abundance
m/z

(A)

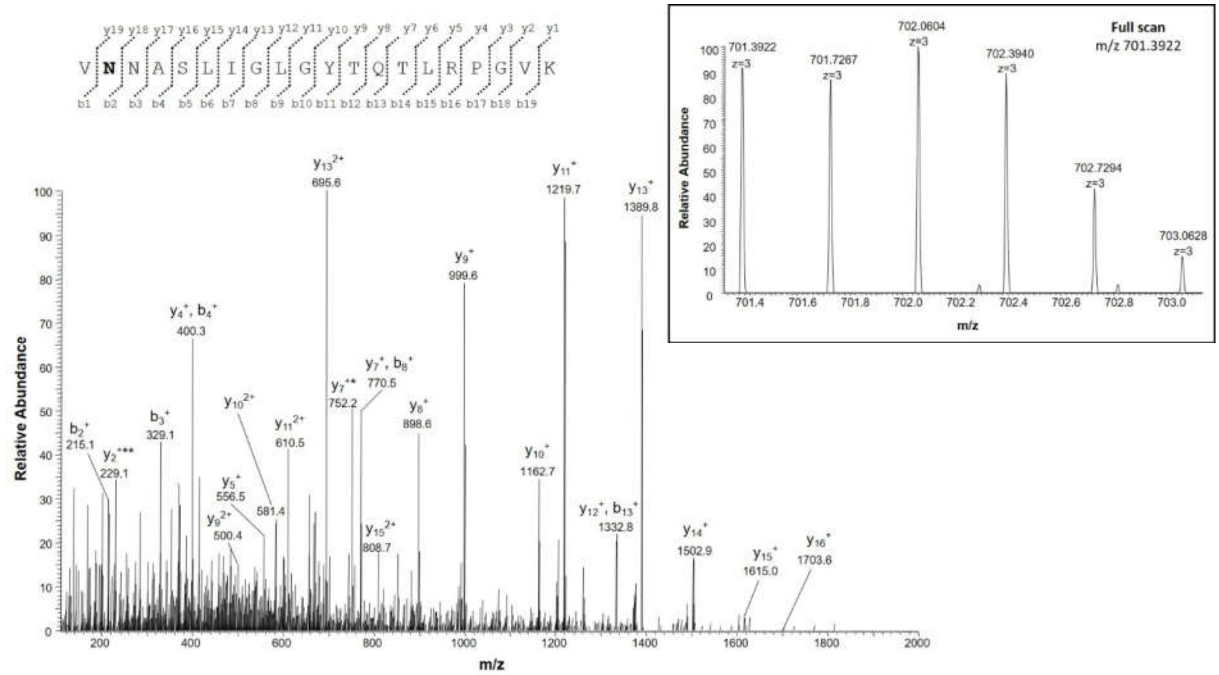

V N N A S L I G L G Y T Q T L R P G V K
Full scan
m/z 701.3922
Relative Abundance
m/z

(B)

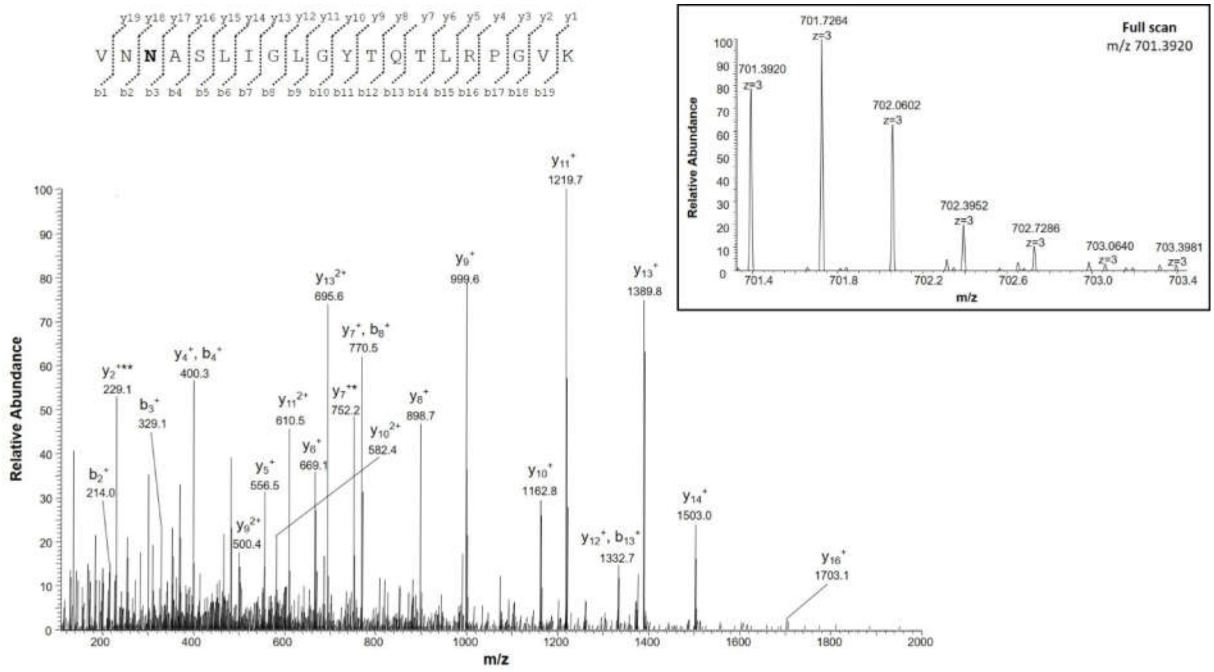
y19 y18 y17 y16 y15 y14 y13 y12 y11 y10 y9 y8 y7 y6 y5 y4 y3 y2 y1
V N N A S L I G L G Y T Q T L R P G V K
b1 b2 b3 b4 b5 b6 b7 b8 b9 b10 b11 b12 b13 b14 b15 b16 b17 b18 b19
Full scan
m/z 701.3920
701.3920 z=3
701.7264 z=3
702.0602 z=3
702.3952 z=3
702.7286 z=3
703.0640 z=3
703.3981 z=3
Relative Abundance
m/z
y11+ 1219.7
y13+ 1389.8
y9+ 999.6
y13 2+ 695.6
y7+, b8+ 770.5
y4+, b4+ 400.3
y2+** 229.1
b3+ 329.1
y11 2+ 610.5
y7+* 752.2
y10 2+ 582.4
y8+ 898.7
y6+ 669.1
y5+ 556.5
b2+ 214.0
y8 2+ 500.4
y10+ 1162.8
y14+ 1503.0
y12+, b13+ 1332.7
y16+ 1703.1

(C)

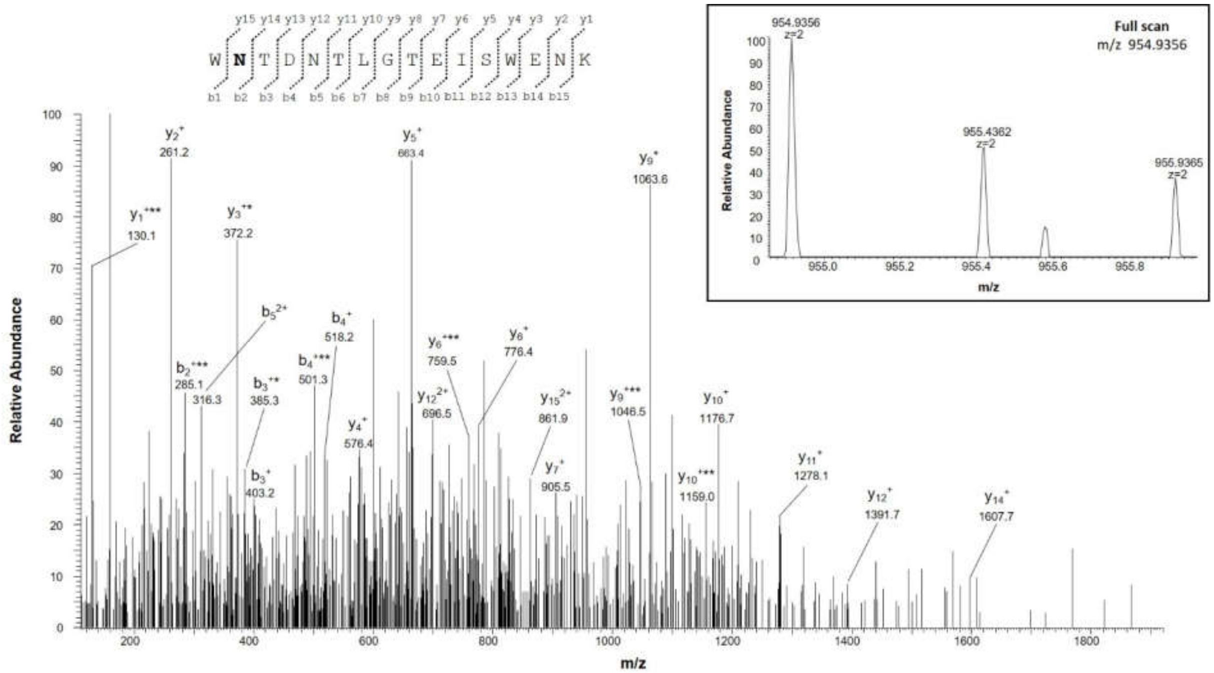
y15 y14 y13 y12 y11 y10 y9 y8 y7 y6 y5 y4 y3 y2 y1
W N T D N T L G T E I S W E N K
b1 b2 b3 b4 b5 b6 b7 b8 b9 b10 b11 b12 b13 b14 b15
Full scan
m/z 954.9356
954.9356 z=2
955.4362 z=2
955.9365 z=2
Relative Abundance
m/z
y2+ 261.2
y5+ 663.4
y9+ 1063.6
y1+** 130.1
y3+* 372.2
b5 2+ 316.3
b4+ 518.2
b2+** 285.1
b4+** 501.3
y6+** 759.5
y6+ 776.4
b3+* 385.3
y12 2+ 696.5
y15 2+ 861.9
y9+** 1046.5
y10+ 1176.7
y4+ 576.4
b3+ 403.2
y7+ 905.5
y10+** 1159.0
y11+ 1278.1
y12+ 1391.7
y14+ 1607.7

(D)

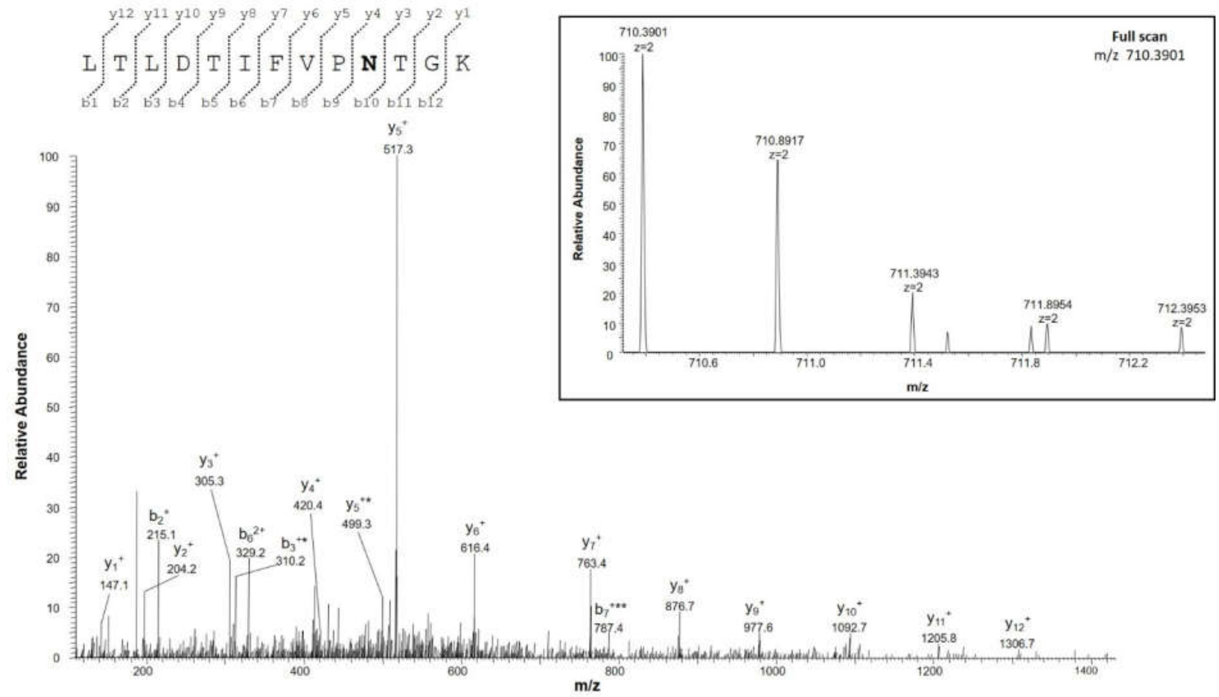

(E)

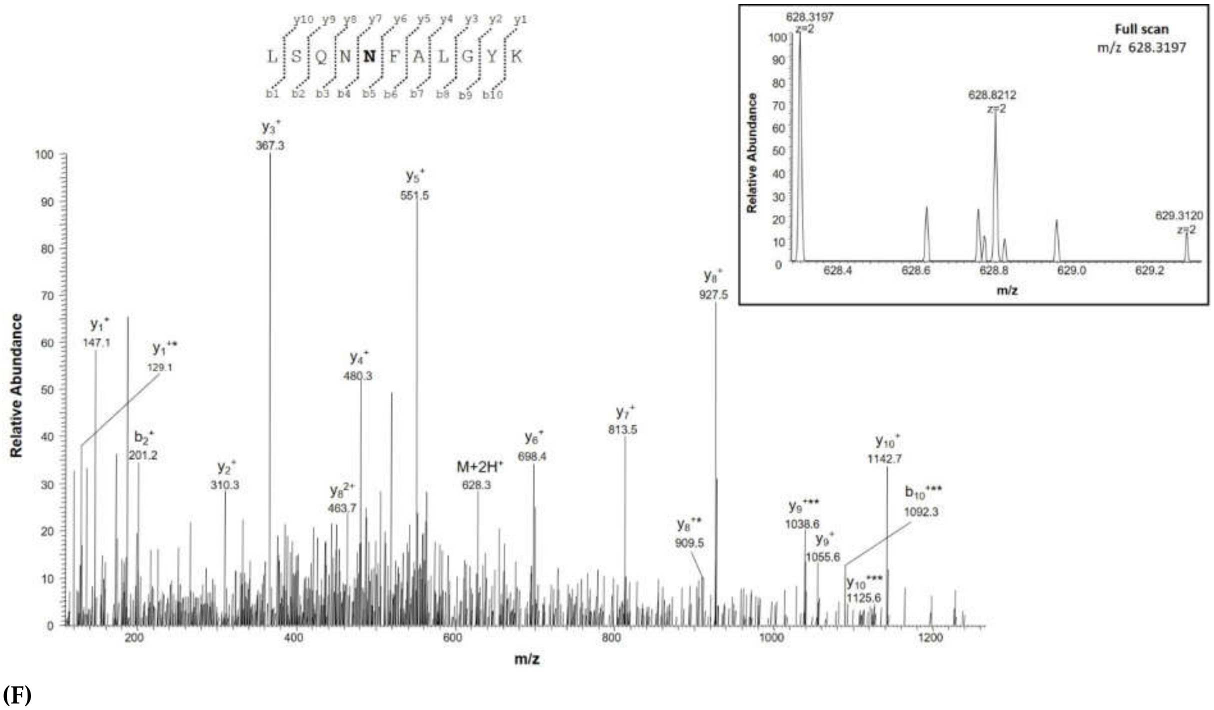

(F)

Supplementary Figure S12. A. MS/MS spectrum of the doubly charged molecular ion at m/z 628.3201 (calculated 628.3197) of the VDAC3 tryptic peptide from NSC34-SOD1G93A cell line containing the asparagine residue 167 in the deamidated form. The inset shows the full scan mass spectrum of molecular ion. Fragment ions originated from the neutral loss of H_2O are indicated by an asterisk. Fragment ions originated from the neutral loss of NH_3 are indicated by two asterisks. **B.** MS/MS spectrum of the triply charged molecular ion at m/z 701.3922 (calculated 701.3920) of the VDAC3 tryptic peptide from NSC34-SOD1G93A cell line containing the asparagine residue 238 in the deamidated form. The inset shows the full scan mass spectrum of molecular ion. Fragment ion originated from the neutral loss of H_2O is indicated by an asterisk. Fragment ion originated from the neutral loss of NH_3 is indicated by two asterisks. **C.** MS/MS spectrum of the triply charged molecular ion at m/z 701.3920 (calculated 701.3920) of the

VDAC3 tryptic peptide from NSC34-SOD1G93A cell line containing the asparagine residue 239 in the deamidated form. The inset shows the full scan mass spectrum of molecular ion. Fragment ion originated from the neutral loss of H_2O is indicated by an asterisk. Fragment ion originated from the neutral loss of NH_3 is indicated by two asterisk. **D.** MS/MS spectrum of the doubly charged molecular ion at m/z 954.9356 (calculated 954.9344) of the VDAC3 tryptic peptide from NSC34-SOD1G93A cell line containing the asparagine residue 76 in the deamidated form. The inset shows the full scan mass spectrum of molecular ion. Fragment ions originated from the neutral loss of H_2O are indicated by an asterisk. Fragment ions originated from the neutral loss of NH_3 are indicated by two asterisk. **E.** MS/MS spectrum of the doubly charged molecular ion at m/z 710.3901 (calculated 710.3904) of the VDAC3 tryptic peptide from NSC34-SOD1G93A cell line containing the asparagine residue 106 in the deamidated form. The inset shows the full scan mass spectrum of molecular ion. Fragment ions originated from the neutral loss of H_2O are indicated by an asterisk. Fragment ion originated from the neutral loss of NH_3 is indicated by two asterisk. **F.** MS/MS spectrum of the doubly charged molecular ion at m/z 628.3197 (calculated 628.3197) of the VDAC3 tryptic peptide from NSC34-SOD1G93A cell line containing the asparagine residue 168 in the deamidated form. The inset shows the full scan mass spectrum of molecular ion. Fragment ions originated from the neutral loss of H_2O are indicated by an asterisk. Fragment ions originated from the neutral loss of NH_3 are indicated by two asterisks.

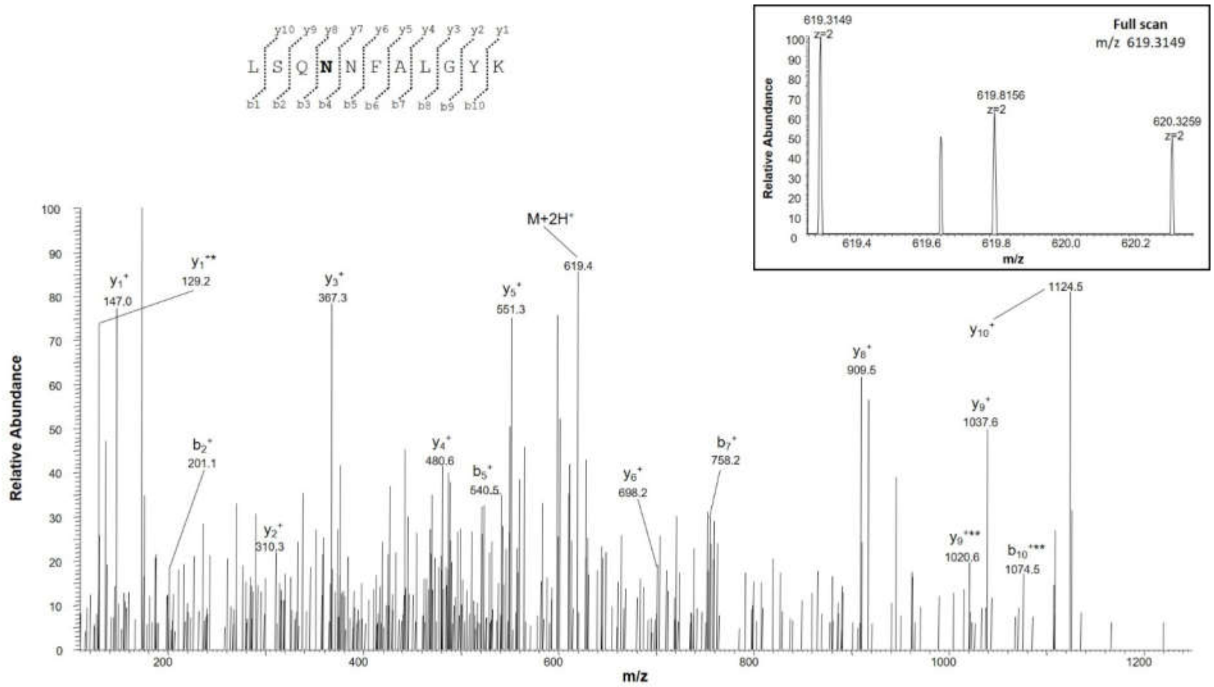

y10 y9 y8 y7 y6 y5 y4 y3 y2 y1
L S Q N N F A L G Y K
b1 b2 b3 b4 b5 b6 b7 b8 b9 b10
Full scan
m/z 619.3149
619.3149
z=2
619.8156
z=2
620.3259
z=2
Relative Abundance
m/z
M+2H+
619.4
y1+
147.0
y1+*
129.2
y3+
367.3
y5+
551.3
1124.5
y10+
y8+
909.5
y9+
1037.6
b2+
201.1
y4+
480.6
b5+
540.5
y6+
698.2
b7+
758.2
y2+
310.3
y9+**
1020.6
b10+**
1074.5

(A)

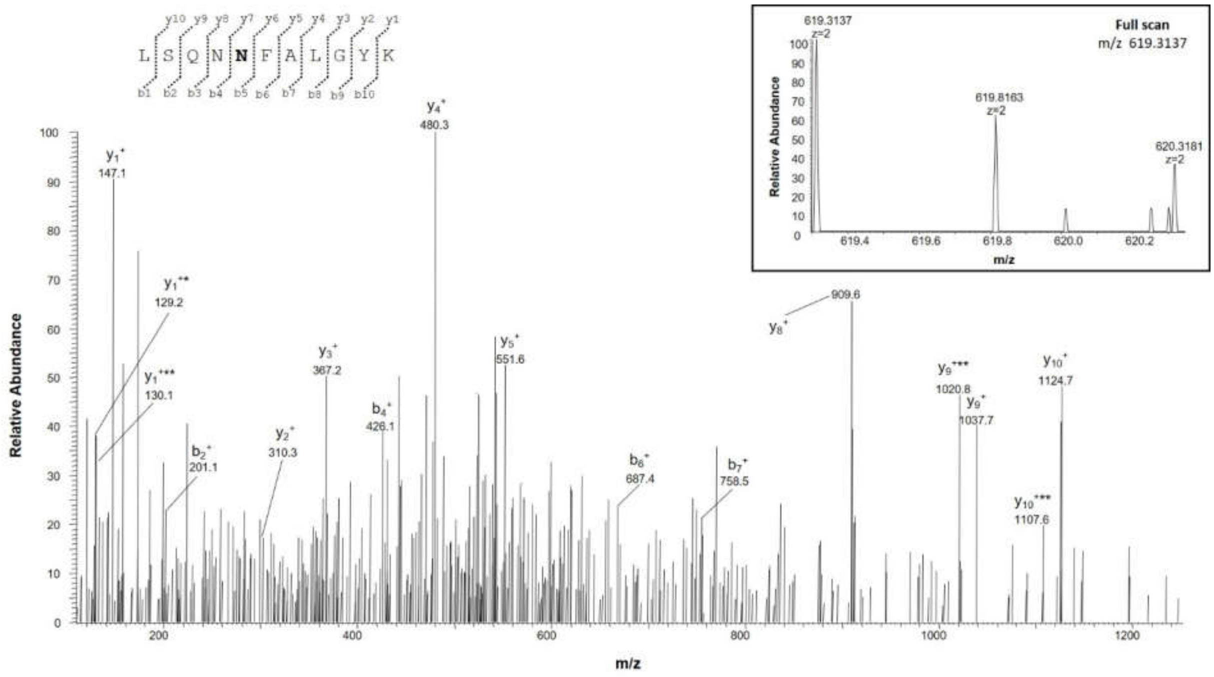

y10 y9 y8 y7 y6 y5 y4 y3 y2 y1
L S Q N N F A L G Y K
b1 b2 b3 b4 b5 b6 b7 b8 b9 b10
Full scan
m/z 619.3137
619.3137
z=2
619.8163
z=2
620.3181
z=2
Relative Abundance
m/z
y4+
480.3
y1+
147.1
y1+*
129.2
y1+**
130.1
909.6
y8+
y3+
367.2
y5+
551.6
b4+
426.1
y9+**
1020.8
y9+
1037.7
y10+
1124.7
b2+
201.1
y2+
310.3
b6+
687.4
b7+
758.5
y10+**
1107.6

(B)

L S Q **N** **N** F A L G Y K

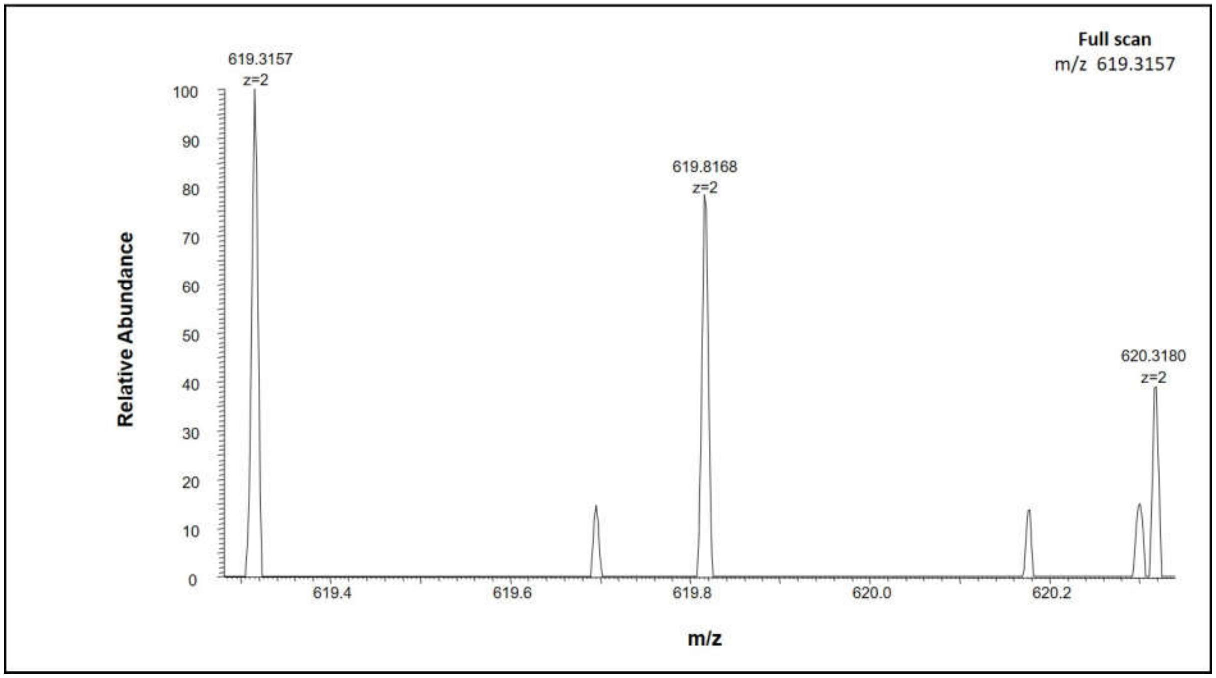

(C)

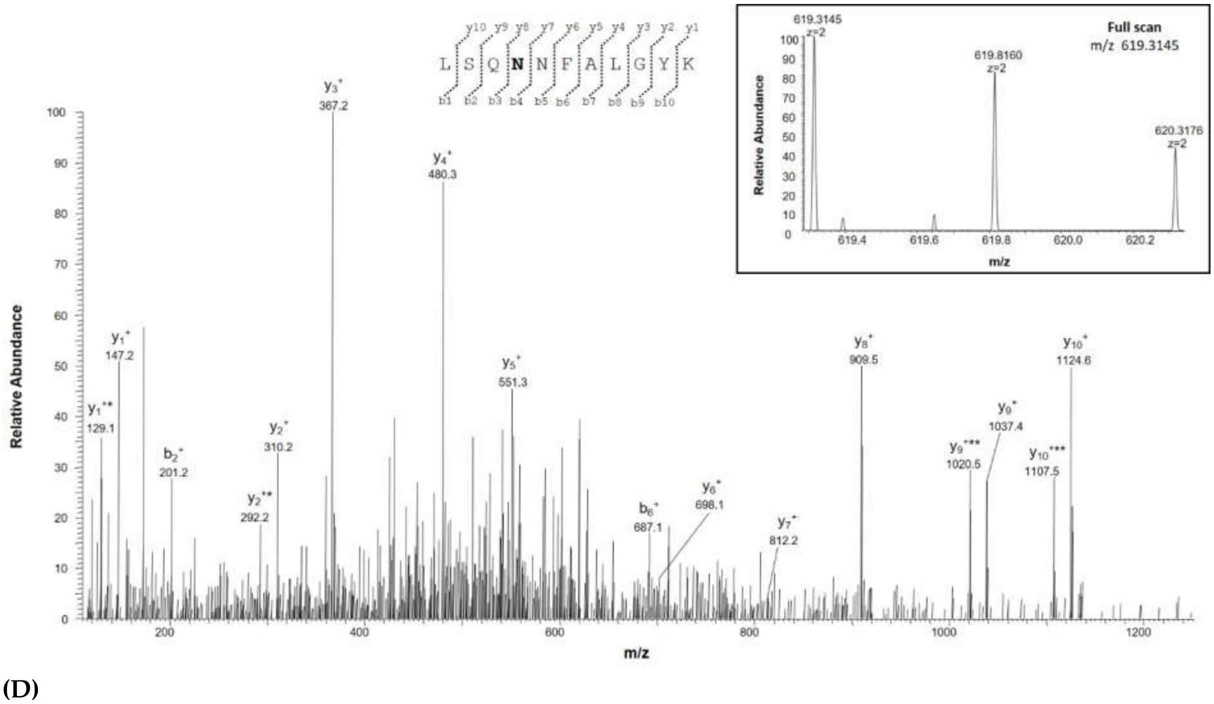

(D)

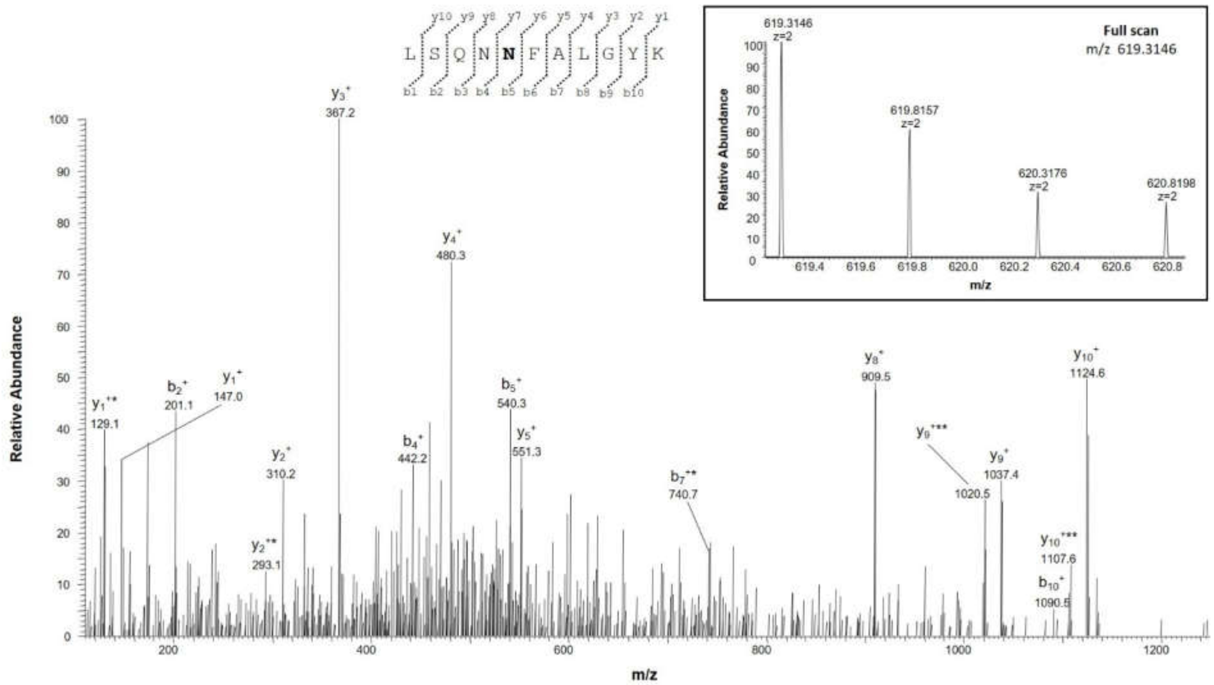
y10 y9 y8 y7 y6 y5 y4 y3 y2 y1
L S Q N N F A L G Y K
b1 b2 b3 b4 b5 b6 b7 b8 b9 b10
Full scan
m/z 619.3146
619.3146 z=2
619.8157 z=2
620.3176 z=2
620.8198 z=2
Relative Abundance
m/z
y3+ 367.2
y4+ 480.3
y1++ 129.1
b2+ 201.1
y1+ 147.0
y2+ 310.2
y2++ 293.1
b4+ 442.2
b5+ 540.3
y5+ 551.3
b7++ 740.7
y8+ 909.5
y9+++ 1020.5
y9+ 1037.4
y10+ 1124.6
y10+++ 1107.6
b10+ 1090.5

(E)

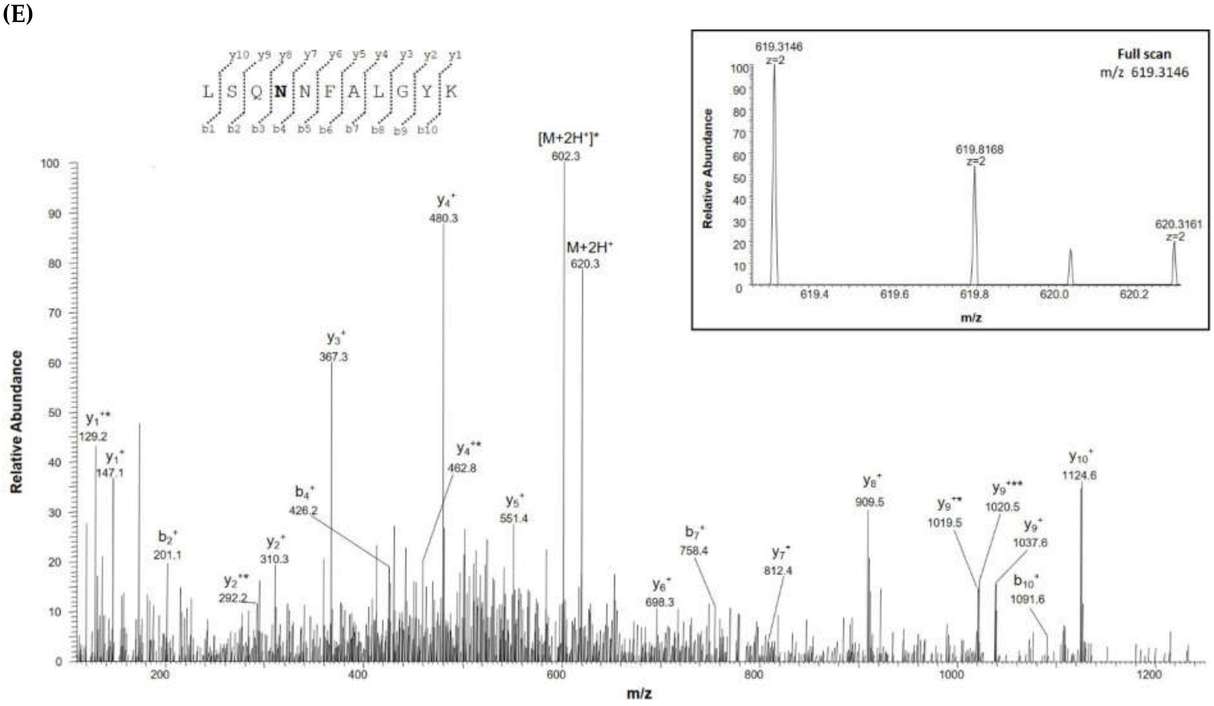
y10 y9 y8 y7 y6 y5 y4 y3 y2 y1
L S Q N N F A L G Y K
b1 b2 b3 b4 b5 b6 b7 b8 b9 b10
Full scan
m/z 619.3146
619.3146 z=2
619.8168 z=2
620.3161 z=2
Relative Abundance
m/z
[M+2H+]+ 602.3
M+2H+ 620.3
y4+ 480.3
y3+ 367.3
y1++ 129.2
y1+ 147.1
y4++ 462.8
b4+ 426.2
y5+ 551.4
b2+ 201.1
y2+ 310.3
y2++ 292.2
y6+ 698.3
b7+ 758.4
y7+ 812.4
y8+ 909.5
y9++ 1019.5
y9+++ 1020.5
y9+ 1037.6
b10+ 1091.6
y10+ 1124.6

(F)

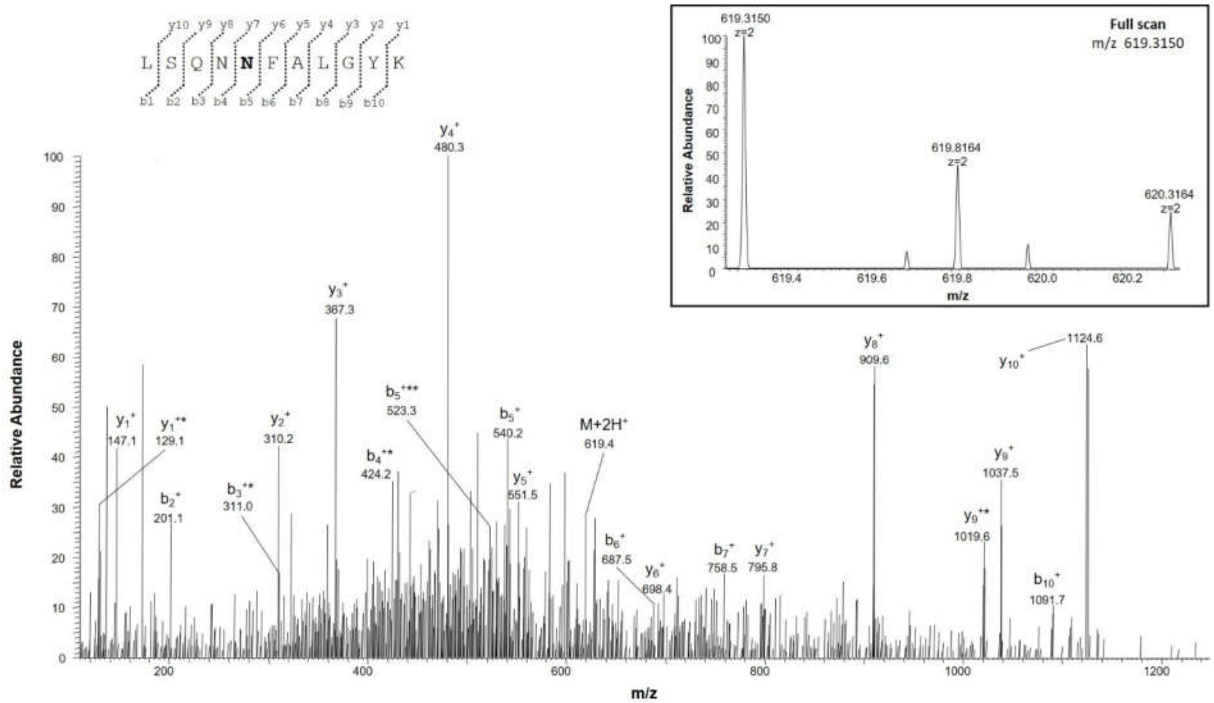

(G)

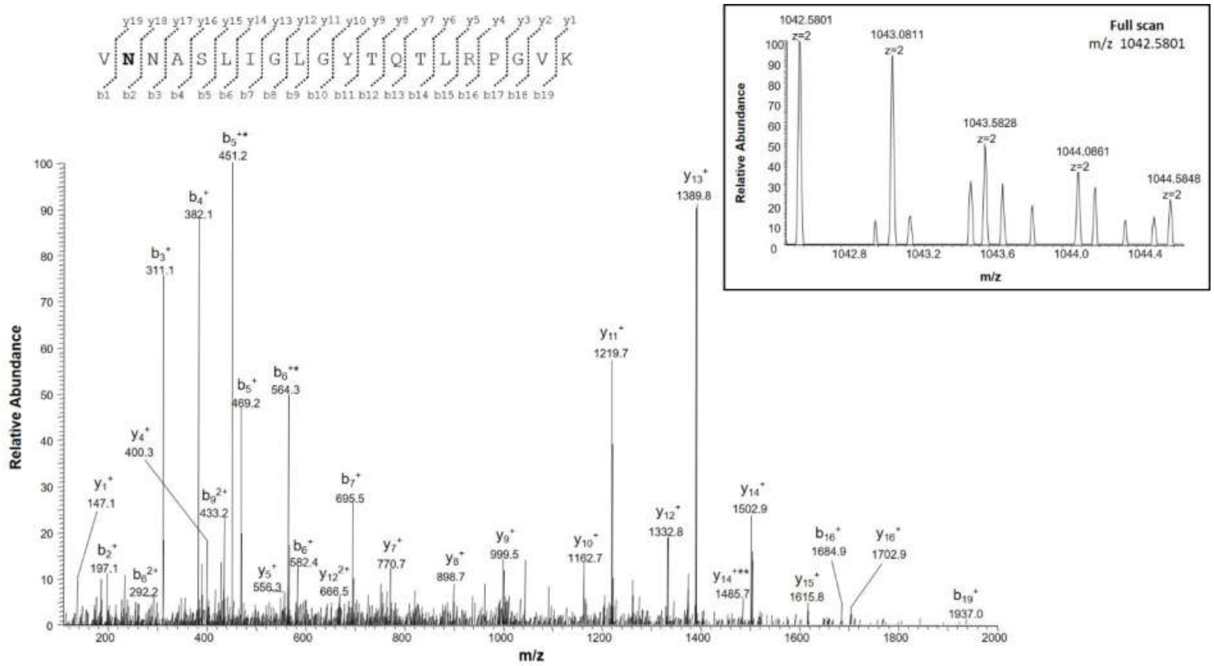

(H)

Supplementary Figure S13. A. MS/MS spectrum of the doubly charged molecular ion at m/z 619.3149 (calculated 619.3144) of the VDAC3 tryptic peptide from NSC34 cell line containing the asparagine residue 167 in succinimide intermediate form. The inset shows the full scan mass spectrum of molecular ion. Fragment ion originated from the neutral loss of H_2O is indicated by an asterisk. Fragment ions originated from the neutral loss of NH_3 are indicated by two asterisks. **B.** MS/MS spectrum of the doubly charged molecular ion at m/z 619.3137 (calculated 619.3144) of the VDAC3 tryptic peptide from NSC34 cell line containing the asparagine residue 168 in succinimide intermediate form. The inset shows the full scan mass spectrum of molecular ion. Fragment ion originated from the neutral loss of H_2O is indicated by an asterisk. Fragment ions originated from the neutral loss of NH_3 are indicated by two asterisks. **C.** Full scan mass spectrum of the doubly charged molecular ion at m/z 619.3157 (calculated 619.3144) of

the VDAC3 tryptic peptide from NSC34 cell line containing the asparagine residue 167 or asparagine residue 168 in succinimide intermediate form. **D.** MS/MS spectrum of the doubly charged molecular ion at m/z 619.3145 (calculated 619.3144) of the VDAC3 tryptic peptide from NSC34-SOD1WT cell line containing the asparagine residue 167 in succinimide intermediate form. The inset shows the full scan mass spectrum of molecular ion. Fragment ions originated from the neutral loss of H_2O are indicated by an asterisk. Fragment ions originated from the neutral loss of NH_3 are indicated by two asterisks. **E.** MS/MS spectrum of the doubly charged molecular ion at m/z 619.3146 (calculated 619.3144) of the VDAC3 tryptic peptide from NSC34-SOD1WT cell line containing the asparagine residue 168 in succinimide intermediate form. The inset shows the full scan mass spectrum of molecular ion. Fragment ions originated from the neutral loss of H_2O are indicated by an asterisk. Fragment ions originated from the neutral loss of NH_3 are indicated by two asterisks. **F.** MS/MS spectrum of the doubly charged molecular ion at m/z 619.3146 (calculated 619.3144) of the VDAC3 tryptic peptide from NSC34-SOD1G93A cell line containing the asparagine residue 167 in succinimide intermediate form. The inset shows the full scan mass spectrum of molecular ion. Fragment ions originated from the neutral loss of H_2O are indicated by an asterisk. Fragment ion originated from the neutral loss of NH_3 is indicated by two asterisks. **G.** MS/MS spectrum of the doubly charged molecular ion at m/z 619.3150 (calculated 619.3144) of the VDAC3 tryptic peptide from NSC34-SOD1G93A cell line containing the asparagine residue 168 in succinimide intermediate form. The inset shows the full scan mass spectrum of molecular ion. Fragment ions originated from the neutral loss of H_2O are indicated by an asterisk. Fragment ion originated from the neutral loss of NH_3 is indicated by two asterisks. **H.** MS/MS spectrum of the doubly charged molecular ion at m/z 1042.5801 (calculated 1042.5788) of the VDAC3 tryptic peptide from NSC34-SOD1WT cell line containing the asparagine residue 238 in succinimide intermediate form. The inset shows the full scan mass spectrum of molecular ion. Fragment ions originated from the neutral loss of H_2O are indicated by an asterisk. Fragment ion originated from the neutral loss of NH_3 is indicated by two asterisks.

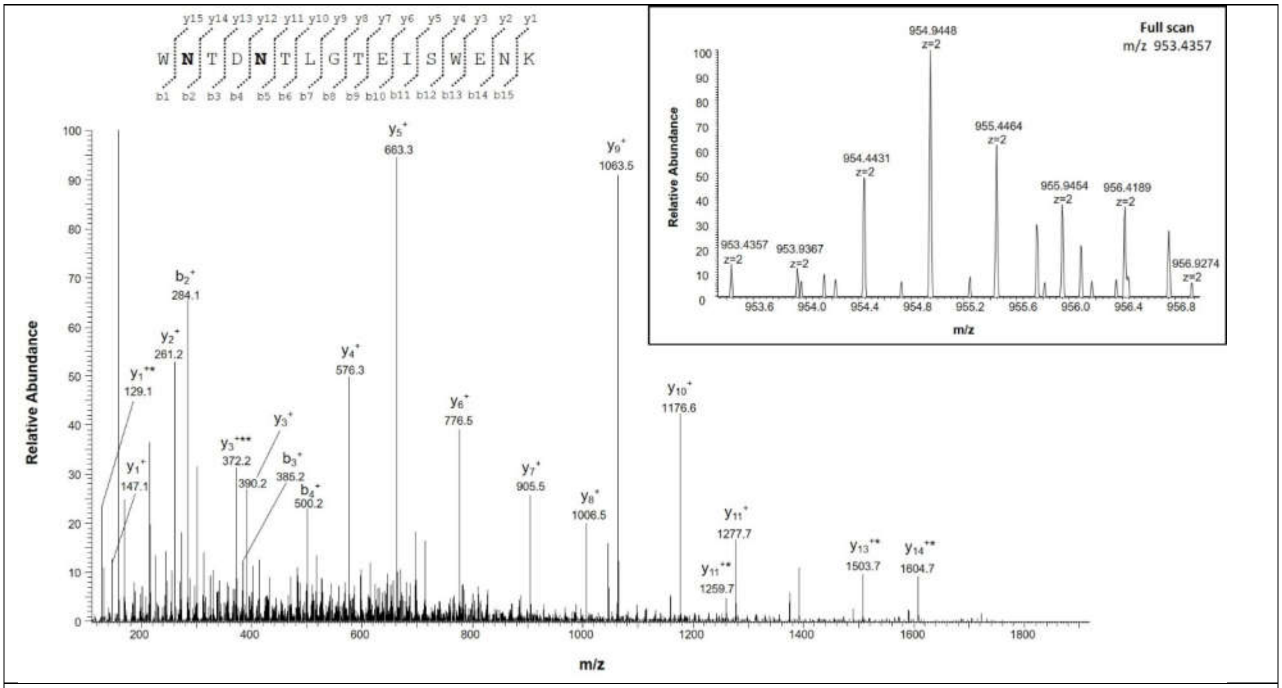

(A)

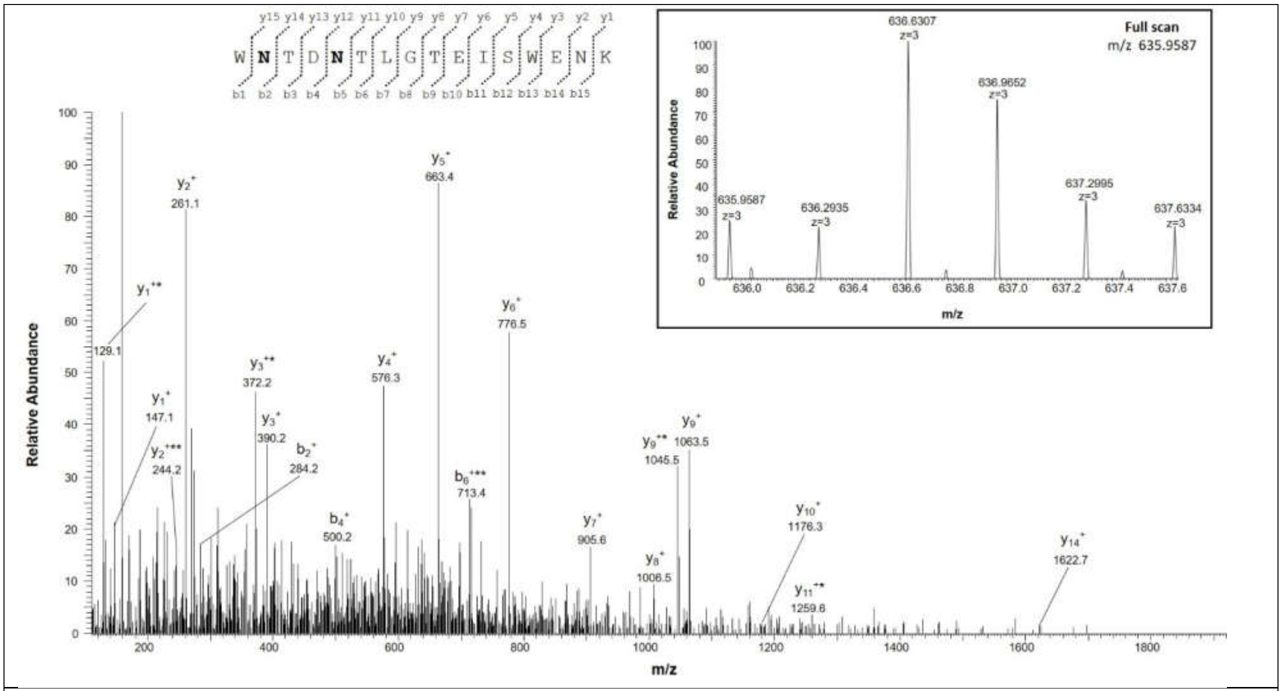

(B)

Supplementary Figure S14. A. MS/MS spectrum of the doubly charged molecular ion at m/z 953.4357 (calculated 953.4408) of the VDAC3 tryptic peptide from NSC34 cell line containing asparagine residue 76 in succinimide intermediate form and asparagine residue 79 in isoaspartate methyl ester form. The inset shows the full scan mass spectrum of molecular ion. Fragment ions originated from the neutral loss of H_2O are indicated by an asterisk. Fragment ion originated from the neutral loss of NH_3 is indicated by two asterisks. **B.** MS/MS spectrum of the triply charged molecular ion at m/z 635.9587 (calculated 635.9632) of the VDAC3 tryptic peptide from NSC34-SOD1WT cell line containing asparagine residue 76 in succinimide intermediate form and asparagine residue 79 in isoaspartate methyl ester form. The inset shows the full scan mass spectrum of molecular ion. Fragment ions originated from the neutral loss of H_2O are indicated by an asterisk. Fragment ion originated from the neutral loss of NH_3 is indicated by two asterisks.

```
CLUSTAL O(1.2.4) multiple sequence alignment

hVDAC3      MCNTPTYCDLGKAAKDVFNKGYGFGMVKIDLKTKSCSGVEFSTSGHAYTDTGKASGNLET    60
mVDAC3      MCNTPTYCDLGKAAKDVFNKGYGFGMVKIDLKTKSCSGVEFSTSGHAYTDTGKASGNLET    60
            ************************************************************

hVDAC3      KYKVCNYGLTFTQKWNTDNTLGTEISWENKLAEGLKLTLDTIFVPNTGKKSGKLKASYKR    120
mVDAC3      KYKVCNYGLTFTQKWNTDNTLGTEISWENKLAEGLKLTLDTIFVPNTGKKSGKLKASYRR    120
            **********************************************************:*

hVDAC3      DCFSVGSNVDIDFSGPTIYGWAVLAFEGWLAGYQMSFDTAKSKLSQNNFALGYKAADFQL    180
mVDAC3      DCFSLGSNVDIDFSGPTIYGWAVLAFEGWLAGYQMSFDTAKSKLSQNNFALGYKAADFQL    180
            ****:*******************************************************

hVDAC3      HTHVNDGTEFGGSIYQKVNEKIETSINLAWTAGSNNTRFGIAAKYMLDCRTSLSAKVNNA    240
mVDAC3      HTHVNDGTEFGGSIYQKVNERIETSINLAWTAGSNNTRFGIAAKYKLDCRTSLSAKVNNA    240
            ********************:************************* *************

hVDAC3      SLIGLGYTQTLRPGVKLTLSALIDGKNFSAGGHKVGLGFELEA 283
mVDAC3      SLIGLGYTQTLRPGVKLTLSALIDGKNFNAGGHKVGLGFELEA 283
            ****************************.**************
```

NW Score	Identities	Positives	Gaps
1481	278/283(98%)	282/283(99%)	0/283(0%)

```
Query  1    MCNTPTYCDLGKAAKDVFNKGYGFGMVKIDLKTKSCSGVEFSTSGHAYTDTGKASGNLET  60
            MCNTPTYCDLGKAAKDVFNKGYGFGMVKIDLKTKSCSGVEFSTSGHAYTDTGKASGNLET
Sbjct  1    MCNTPTYCDLGKAAKDVFNKGYGFGMVKIDLKTKSCSGVEFSTSGHAYTDTGKASGNLET  60

Query  61   KYKVCNYGLTFTQKWNTDNTLGTEISWENKLAEGLKLTLDTIFVPNTGKKSGKLKASYKR  120
            KYKVCNYGLTFTQKWNTDNTLGTEISWENKLAEGLKLTLDTIFVPNTGKKSGKLKASY+R
Sbjct  61   KYKVCNYGLTFTQKWNTDNTLGTEISWENKLAEGLKLTLDTIFVPNTGKKSGKLKASYRR  120

Query  121  DCFSVGSNVDIDFSGPTIYGWAVLAFEGWLAGYQMSFDTAKSKLSQNNFALGYKAADFQL  180
            DCFS+GSNVDIDFSGPTIYGWAVLAFEGWLAGYQMSFDTAKSKLSQNNFALGYKAADFQL
Sbjct  121  DCFSLGSNVDIDFSGPTIYGWAVLAFEGWLAGYQMSFDTAKSKLSQNNFALGYKAADFQL  180

Query  181  HTHVNDGTEFGGSIYQKVNEKIETSINLAWTAGSNNTRFGIAAKYMLDCRTSLSAKVNNA  240
            HTHVNDGTEFGGSIYQKVNE+IETSINLAWTAGSNNTRFGIAAKY LDCRTSLSAKVNNA
Sbjct  181  HTHVNDGTEFGGSIYQKVNERIETSINLAWTAGSNNTRFGIAAKYKLDCRTSLSAKVNNA  240

Query  241  SLIGLGYTQTLRPGVKLTLSALIDGKNFSAGGHKVGLGFELEA  283
            SLIGLGYTQTLRPGVKLTLSALIDGKNF+AGGHKVGLGFELEA
Sbjct  241  SLIGLGYTQTLRPGVKLTLSALIDGKNFNAGGHKVGLGFELEA  283
```

Supplementary Figure S15. Sequence alignments of hVDAC3 and mVDAC3 proteins obtained with Clustal omega (left) and with BLAST (right). The percentage of identity (98%) and similarity (99%) indicates no significant differences between hVDAC3 and mVDAC3 proteins.

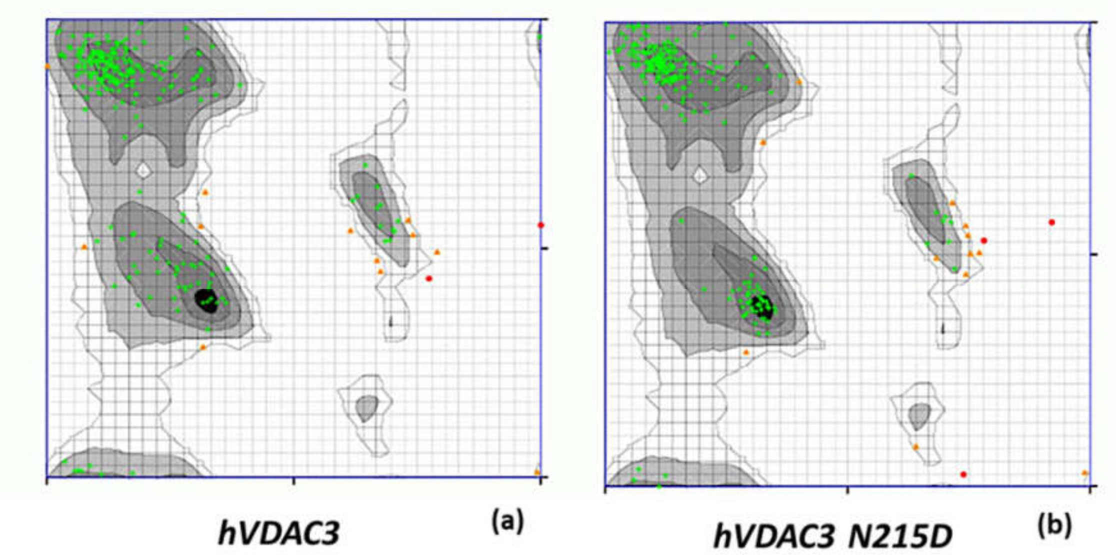

Supplementary Figure S16. Ramachandran plots (RP) for hVDAC3 wt **(A)** and hVDAC3 N215D **(B)** were produced by the free web tool https://zlab.umassmed.edu/bu/rama/. Dark gray areas are the most favored conformations; in light gray areas the conformations are also favored but show higher stericity. Orange triangles in the white area represent amino acids with a permitted conformation but with a very high degree of stericity, while red dots represent amino acids with outlier conformations.

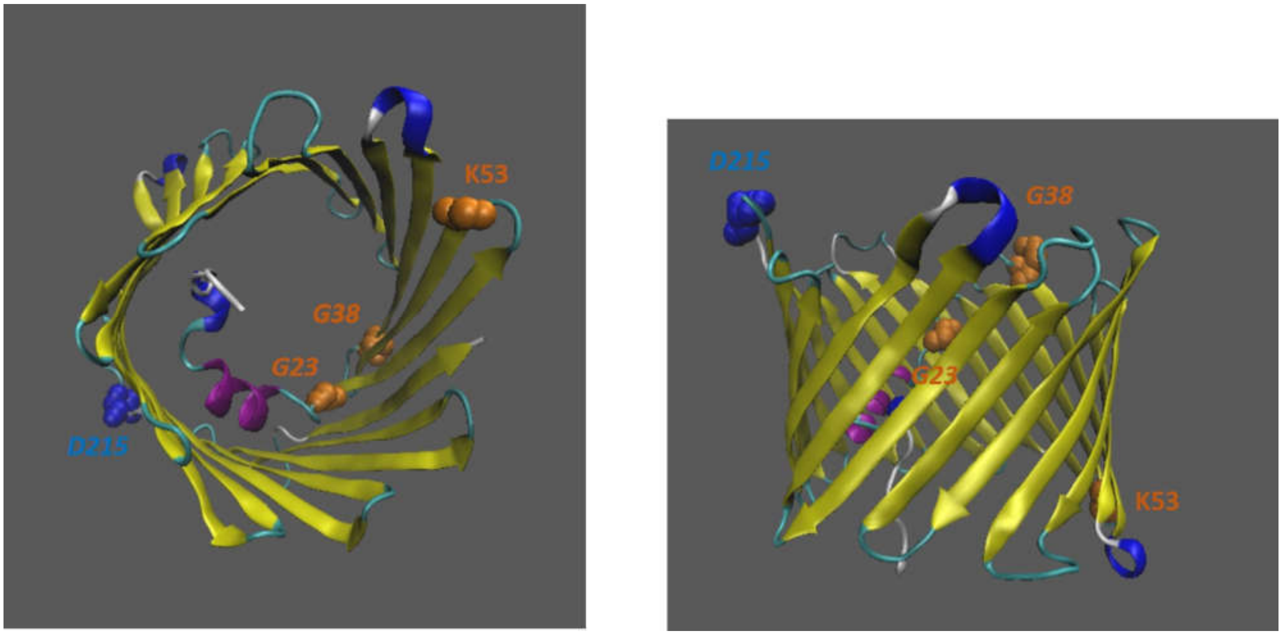

Supplementary Figure S17. Structural predictions of hVDAC3 obtained by VMD software: top view (on the left) and side view (on the right) were. In blue, the Asn215 residue subjected to deamination in Asp in NSC34 SOD1G93A cells. In orange, amino acids located in 'not allowed' regions in RP of VDAC3 N215D.

TABLES

Table S1. Tryptic peptides found in the analysis of VDAC3 from the cell line NSC34 after DTT reduction, carboxyamidomethylation and in-solution digestion.

The retention time, experimentally measured and calculated monoisotopic m/z of the molecular ions, position in the sequence and peptide sequence of the fragments present in the tryptic digest analyzed in triplicate of reduced and carboxyamidomethylated VDAC3 are reported. All sequences were confirmed by MS/MS. These sequences were used to construct the sequence coverage shown in Figure 1.

Technical triplicate	Frag. n.	Rt (min)	Monoisotopic m/z		Position in the sequence	Peptide sequence
			Measured	Calculated		
I	1	40.36	685.7902 (2+)	685.7897	2-12	[*]*C*NTPTY*C*DLGK
II		39.88				
III		39.72	685.7901 (2+)			
I	2	38.33	429.7125 (2+)	429.7128	21-28	GYGFGMVK
II		37.74	429.7124 (2+)			
III		37.53				
I	3	28.56	995.9264 (2+)	995.9260	35-53	S*C*SGVEFSTSGHAYTDTGK
II		28.08	995.9267 (2+)			
III		27.60	995.9265 (2+)			
I	4	37.92	811.4069 (2+)	811.4060	62-74	YKV*C*NYGLTFTQK
II		37.32	811.4071 (2+)			
III		37.19	811.4070 (2+)			
I	5	40.18	665.8275 (2+)	665.8269	64-74	V*C*NYGLTFTQK
II		39.71	665.8273 (2+)			
III		39.58	665.8274 (2+)			
I	6	51.12	954.4430 (2+)	954.4424	75-90	WNTDNTLGTEISWENK
II		50.68				
III		50.65	954.4432 (2+)			
I	7	57.93	677.3889 (3+)	677.3893	91-109	LAEGLKLTLDTIFVPNTGK
II		57.51	677.3898 (3+)			
I	8	53.23	720.0882 (3+)	720.0876	91-110	LAEGLKLTLDTIFVPNTGKK
I	9	55.35	709.8995 (2+)	709.8984	97-109	LTLDTIFVPNTGK
II		54.91	709.8989 (2+)			
III		54.85	709.8993 (2+)			
I	10	47.80	773.9462 (3+)	773.9456	97-110	LTLDTIFVPNTGKK
II		47.33	516.2998 (3+)	516.2999		
III		47.26	773.9462 (2+)	773.9456		
I	11	31.84	735.3917 (2+)	735.3910	162-174	SKLSQNNFALGYK

II		31.43	735.3921 (2+)			
III		31.11	490.5969 (3+)	490.5967		
I	12	38.78	627.8282 (2+)	627.8277	164-174	LSQNNFALGYK
II		38.15				
III		38.00	627.8281 (2+)			
I	13	39.68	845.7355 (3+)	845.7352	175-197	AADFQLHTHVNDGTEFGGSIYQK
II		39.39				
III		39.30	845.7353 (3+)			
I	14	49.29	616.6469 (2+)	616.6464	202-218	IETSINLAWTAGSNNTR
II		48.83				
III		48.81	924.4666 (2+)	924.4662		
I	15	45.44	896.8400 (2+)	896.8396	231-256	TSLSAKVNNASLIGLGYTQTLRPGVK
II		45.02	896.8396 (2+)			
III		44.93	896.8400 (3+)			
I	16	50.91	860.4736 (2+)	860.4736	237-252	VNNASLIGLGYTQTLR
II		50.46	860.4732 (2+)			
III		50.42	860.4740 (2+)			
I	17	74.30	701.0646 (3+)	701.0637	237-256	VNNASLIGLGYTQTLRPGVK
II		44.94				
III		43.09	701.0698 (3+)			
III	18	43.79	619.3395 (3+)	619.3390	257-274	LTLSALIDGKNFNAGGHK
I	19	60.54	934.4886 (1+)	934.4880	275-283	VGLGFELEA
II		60.21	934.4892 (1+)			
III		60.06	467.7475 (2+)	467.7477		

C*: N-terminal acetylated; ***C*: cysteine carboxyamidomethylated.

Table S2. Chymotryptic peptides found in the analysis of VDAC3 from the cell line NSC34 after DTT reduction carboxyamidomethylation and in-solution digestion.
The retention time, experimentally measured and calculated monoisotopic m/z of the molecular ions, position in the sequence and peptide sequence of fragments present in the chymotryptic digest analyzed in triplicate of reduced and carboxyamidomethylated VDAC3 are reported. These sequences were used to construct the sequence coverage shown in Figure 1.

**Technical replicate**	**Frag. n.**	**Rt (min)**	**Monoisotopic m/z**		**Position in the sequence**	**Peptide sequence**
			**Measured**	**Calculated**		
I	1	34.32	820.8748 (2+)	820.8745	2-15	**C*NTPTY***C*DLGKAAK
II		34.46	820.8748 (2+)			
III		34.10	820.8749 (2+)			

I	2	53.70	1001.4565 (2+)	1001.4562	2-18	*CNTPTYCDLGKAAKDVF
II		53.73	1001.4564 (2+)			
III		53.72	1001.4562 (2+)			
I	3	35.22	408.5412 (3+)	408.5417	8-18	CDLGKAAKDVF
II		35.40	612.3085 (2+)	612.3084		
III		35.05	612.3086 (2+)			
I	4	54.74	490.2672 (2+)	490.2682	23-31	GFGMVKIDL
II		54.79	490.2674 (2+)			
III		54.76	490.2677 (2+)			
I	5	30.59	685.8349 (2+)	685.8352	30-41	DLKTKSCSGVEF
II		30.89	685.8350 (2+)			
III		30.57	685.8351 (2+)			
I	6	22.06	730.0109 (3+)	730.0105	42-62	STSGHAYTDTGKASGNLETKY
III		22.26	730.0103 (3+)			
I	7	25.69	859.9149 (2+)	859.9158	47-62	AYTDTGKASGNLETKY
II		26.10	859.9156 (2+)			
III		25.68	859.9161 (2+)			
I	8	20.20	742.8656 (2+)	742.8653	49-62	TDTGKASGNLETKY
II		20.32	742.8654 (2+)			
III		20.21	742.8655 (2+)			
II	9	21.92	491.7459 (2+)	491.7462	54-62	ASGNLETKY
III		21.75	491.7461 (2+)			
III	10	58.05	675.8129 (2+)	675.8128	76-87	NTDNTLGTEISW
I	11	59.52	1103.0387 (2+)	1103.0377	76-95	NTDNTLGTEISWENKLAEGL
II		59.60	1103.0386 (2+)			
III		59.61	1103.0388 (2+)			
II	12	48.10	516.2998 (3+)	516.3000	96-109	KLTLDTIFVPNTGK
I	13	40.76	558.9982 (3+)	558.9984	96-110	KLTLDTIFVPNTGKK
II		40.88	558.9977 (3+)			
III		40.63	558.9981 (3+)			
II	14	38.13	687.4089 (3+)	687.4092	96-114	KLTLDTIFVPNTGKKSGKL
II	15	38.37	717.4039 (2+)	717.4041	98-110	TLDTIFVPNTGKK
III		37.99	717.4041 (2+)			
I	16	35.37	607.0163 (3+)	607.0162	98-114	TLDTIFVPNTGKKSGKL
II		35.52	607.0161 (3+)			
III		35.18	607.0158 (3+)			
II	17	29.30	610.3383 (2+)	610.3382	100-110	DTIFVPNTGKK

III		29.01	610.3375 (2+)			
I	18	28.04	535.6387 (3+)	535.6390	100-114	DTIFVPNTGKKSGKL
II		28.49	535.6383 (3+)			
III		28.26	535.6385 (3+)			
I	19	57.05	1066.5061 (1+)	1066.5057	124-133	SLGSNVDIDF
II		57.11	1066.5059 (1+)			
III		57.10	1066.5062 (1+)			
I	20	62.56	842.9081 (2+)	842.9074	124-139	SLGSNVDIDFSGPTIY
II		62.56	842.9078(2+)			
III		62.61	842.9078 (2+)			
I	21	72.45	628.3217 (2+)	628.3217	154-164	QMSFDTAKSKL
II		78.88	628.3201 (2+)			
III		73.53	628.3201 (2+)			
I	22	20.97	498.7718 (2+)	498.7722	156-164	SFDTAKSKL
II		21.09	498.7724 (2+)			
III		20.96	498.7724 (2+)			
I	23	31.48	793.8953 (2+)	793.8946	156-169	SFDTAKSKLSQNNF
II		31.68	793.8948 (2+)			
III	24	22.54	676.8440 (2+)	676.8444	158-169	DTAKSKLSQNNF
I	25	37.61	690.8131 (2+)	690.8131	179-190	**Q**LHTHVNDGTEF
II		37.77	690.8132 (2+)			
III		37.39	690.8130 (2+)			
I	26	24.95	466.5533 (3+)	466.5535	179-190	QLHTHVNDGTEF
II		25.28	699.3265 (2+)	699.3261		
III		25.02	699.3263 (2+)			
I	27	47.80	929.4249 (2+)	929.4243	179-195	**Q**LHTHVNDGTEFGGSIY
II		47.82	929.4248 (2+)			
III		47.82	929.4247 (2+)			
I	28	37.25	625.6279 (3+)	625.6277	179-195	QLHTHVNDGTEFGGSIY
III		37.06	937.9377 (2+)			
I	29	24.65	510.2251 (2+)	510.2256	182-190	THVNDGTEF
II		25.03	510.2251 (2+)			
III		24.80	510.2253 (2+)			
I	30	41.22	748.8369 (2+)	748.8368	182-195	THVNDGTEFGGSIY
II		41.34	748.8369 (2+)			
III		41.10	748.8371 (2+)			
III	31	35.49	650.3481 (2+)	650.3493	196-206	**Q**KVNERIETSI

II	32	35.86	707.3706 (2+)	707.3708	196-207	**Q**KVNERIETSIN
I	33	44.94	509.6103 (3+)	509.6112	196-208	**Q**KVNERIETSINL
II		44.96	509.6103 (3+)			
III		44.90	763.9132 (2+)	763.9128		
I	34	35.55	772.4262 (2+)	772.4261	196-208	QKVNERIETSINL
II		35.71	772.4262 (2+)			
III		35.35	772.4261 (2+)			
II	35	55.13	892.4713 (2+)	892.4710	196-210	**Q**KVNERIETSINLAW
I	36	47.35	600.9933 (3+)	600.9922	196-210	QKVNERIETSINLAW
III		47.36	600.9924 (3+)			
I	37	40.83	537.7938 (2+)	537.7936	200-208	ERIETSINL
II		40.91	537.7936 (2+)			
III		40.60	537.7935 (2+)			
II	38	19.24	496.7635 (2+)	496.7638	226-233	KLD***C***RTSL
III		19.16	496.7635 (2+)			
I	39	42.56	593.8436 (2+)	593.8437	234-245	SAKVNNASLIGL
II		42.67	593.8435 (2+)			
III		42.44	593.8433 (2+)			
I	40	46.12	703.8868 (2+)	703.8861	234-247	SAKVNNASLIGLGY
II		46.20	703.8871 (2+)			
III		46.14	703.8865 (2+)			
I	41	50.69	589.8251 (2+)	589.8249	258-268	TLSALIDGKNF
II		50.81	589.8245 (2+)			
III		50.77	589.8248 (2+)			
I	42	37.48	482.7589 (2+)	482.7591	260-268	SALIDGKNF
III		37.75	482.7585 (2+)			
I	43	27.03	528.7828 (2+)	528.7834	269-279	NAGGHKVGLGF
II		27.45	528.7834 (2+)			
III		27.17	528.7828 (2+)			

C*: N-terminal acetylated ;*C***: cysteine carboxyamidomethylated; **Q**: pyroglutamic acid form.

Table S3. Retention time, experimentally measured and calculated monoisotopic m/z of the molecular ions, position in the sequence and peptide sequence of sulfur containing tryptic fragments found in the analysis of VDAC3 from the NSC34 cell line reduced with DTT, carboxyamidomethylated and digested in-solution. Fragment 5 was used to construct the sequence coverage shown in Figure 1.

Technical triplicate	Frag. n.	Rt (min)	Monoisotopic m/z		Position in the sequence	Peptide sequence
			Measured	Calculated		

I		30.41	437.7104 (2+)			
II	1	29.92	437.7104 (2+)	437.7103	21-28	GYGFG**M**VK
III		29.58	437.7104 (2+)			
I		33.14	991.4087 (2+)			
II	2	32.61	991.4086 (2+)	991.4079	35-53	S**C**SGVEFSTSGHAYTDTGK
III		32.29	991.4083 (2+)			
I		44.17	806.8884 (2+)			
II	3	43.58	806.8884 (2+)	806.8877	62-74	YKV**C**NYGLTFTQK
III		43.53	806.8884(2+)			
I		48.57	661.3068 (2+)			
II	4	48.10	661.3078 (2+)	661.3085	64-74	V**C**NYGLTFTQK
III		48.02	661.3087 (2+)			
I		88.95	1518.0330 (3+)			
II	5	88.92	1518.0326 (3+)	1518.0315	121-161	D*C*FSLGSNVDIDFSGPTIYGW AVLAFEGWLAGYQ**M**SFDTA K
III		88.57	1518.0348 (3+)			
I		22.83	674.3146 (2+)			
II	6	22.35	674.3146 (2+)	674.3166	150-161	LAGYQ**M**SFDTAK
III		21.96	674.3148 (2+)			

C: cysteine carboxyamidomethylated; **C**: cysteine oxidized to sulfonic acid; **M**: methionine sulfoxide.

Table S4. Retention time, experimentally measured and calculated monoisotopic m/z of the molecular ions, position in the sequence and peptide sequence of sulfur containing chymotryptic fragments found in the analysis of VDAC3 from the NSC34 cell line reduced with DTT, carboxyamidomethylated and digested in-solution.

Technical triplicate	Frag. n.	Rt (min)	Monoisotopic m/z		Position in the sequence	Peptide sequence
			Measured	Calculated		
I		49.78	498.2652 (2+)			
II	1	49.90	498.2652 (2+)	498.2657	23-31	GFG**M**VKIDL
III		49.82	498.2651 (2+)			
I		69.45	636.3119 (2+)			
II	2	70.57	636.3101 (2+)	636.3192	154-164	Q**M**SFDTAKSKL
III		69.17	636.3193 (2+)			
I		22.98	492.2451 (2+)			
II	3	23.27	492.2453 (2+)	492.2455	226-233	KLD**C**RTSL
III		23.08	492.2452 (2+)			

C: cysteine oxidized to sulfonic acid; **M**: methionine sulfoxide.

Table S5. Ox/Red ratio of the absolute intensities of the molecular ions of sulfur containing tryptic peptides found in the analysis of VDAC3 from NSC34 cell line reduced with DTT, carboxyamidomethylated and digested in-solution.

Technical replicate	Peptide	Position in the sequence	Measured monoisotopic m/z	Absolute intensity	Ratio Ox/Red
I	GYGFG**M**VK	21-28	437.7104 (2+)	$4.9 \cdot 10^{6}$	10.2

	GYGFGMVK		429.7125 (2+)	$4.8 \cdot 10^5$	
II	GYGFG**M**VK		437.7104 (2+)	$4.4 \cdot 10^6$	9.2
	GYGFGMVK		429.7124 (2+)	$4.8 \cdot 10^5$	
III	GYGFG**M**VK		437.7104 (2+)	$4.6 \cdot 10^6$	10.9
	GYGFGMVK		429.7124 (2+)	$4.2 \cdot 10^5$	
I	S**C**SGVEFSTSGHAYTDTGK	35-53	991.4087 (2+)	$1.7 \cdot 10^4$	0.07
	S*C*SGVEFSTSGHAYTDTGK		995.9264 (2+)	$2.3 \cdot 10^5$	
II	S**C**SGVEFSTSGHAYTDTGK		991.4086 (2+)	$2.2 \cdot 10^4$	0.09
	S*C*SGVEFSTSGHAYTDTGK		995.9267 (2+)	$2.3 \cdot 10^5$	
III	S**C**SGVEFSTSGHAYTDTGK		991.4083 (2+)	$1.8 \cdot 10^4$	0.08
	S*C*SGVEFSTSGHAYTDTGK		995.9265 (2+)	$2.3 \cdot 10^5$	
I	YKV**C**NYGLTFTQK	62-74	806.8884 (2+)	$2.3 \cdot 10^5$	0.9
	YKV*C*NYGLTFTQK		811.4069 (2+)	$2.6 \cdot 10^5$	
II	YKV**C**NYGLTFTQK		806.8884 (2+)	$2.3 \cdot 10^5$	0.9
	YKV*C*NYGLTFTQK		811.4071(2+)	$2.6 \cdot 10^5$	
III	YKV**C**NYGLTFTQK		806.8884(2+)	$2.1 \cdot 10^5$	0.8
	YKV*C*NYGLTFTQK		811.4070 (2+)	$2.5 \cdot 10^5$	

C: cysteine carboxyamidomethylated; **C**: cysteine oxidized to sulfonic acid; **M**: methionine sulfoxide.

Table S6. Ox/Red ratio of the absolute intensities of molecular ions of sulfur containing chymotryptic peptides found in the analysis of VDAC3 from NSC34 cell line reduced with DTT, carboxyamidomethylated and digested in-solution.

Technical replicate	Peptide	Position in the sequence	Measured monoisotopic *m/z*	Absolute intensity	Ratio Ox/Red
I	GFG**M**VKIDL	23-31	498.2652 (2+)	$1.1 \cdot 10^6$	13.4
	GFGMVKIDL		490.2672 (2+)	$8.2 \cdot 10^4$	
II	GFG**M**VKIDL		498.2652 (2+)	$1.2 \cdot 10^6$	12.6
	GFGMVKIDL		490.2674 (2+)	$9.5 \cdot 10^4$	
III	GFG**M**VKIDL		498.2651 (2+)	$1.2 \cdot 10^6$	9.2
	GFGMVKIDL		490.2677 (2+)	$1.3 \cdot 10^5$	
I	Q**M**SFDTAKSKL	154-164	636.3119 (2+)	$1.2 \cdot 10^4$	0.8
	QMSFDTAKSKL		628.3217 (2+)	$1.6 \cdot 10^4$	
II	Q**M**SFDTAKSKL		636.3101 (2+)	$9.2 \cdot 10^3$	0.5
	QMSFDTAKSKL		628.3201 (2+)	$1.7 \cdot 10^4$	
III	Q**M**SFDTAKSKL		636.3193 (2+)	$9.5 \cdot 10^3$	0.2
	QMSFDTAKSKL		628.3201 (2+)	$4.2 \cdot 10^4$	
II	KLD**C**RTSL	226-233	492.2453 (2+)	$2.0 \cdot 10^5$	0.1
	KLD*C*RTSL		496.7635 (2+)	$1.7 \cdot 10^6$	
III	KLD**C**RTSL		492.2452 (2+)	$1.7 \cdot 10^5$	0.1
	KLD*C*RTSL		496.7635 (2+)	$2.6 \cdot 10^6$	

C: cysteine carboxyamidomethylated; **C**: cysteine oxidized to sulfonic acid; **M**: methionine sulfoxide.

Table S7. Tryptic peptides found in the analysis of VDAC3 from the NSC34-SOD1WT cell line after DTT reduction, carboxyamidomethylation and in-solution digestion.
The retention time, experimentally measured and calculated monoisotopic m/z of the molecular ions, position in the sequence and peptide sequence of fragments present in the tryptic digest analyzed in triplicate of reduced and carboxyamidomethylated VDAC3 are reported. All sequences were confirmed by MS/MS. These sequences were used to construct the sequence coverage reported in Figure 1.

Technical triplicate	Frag. n.	Rt (min)	Monoisotopic m/z		Position in the sequence	Peptide sequence
			Measured	Calculated		

I		40.73	685.7900 (2+)			
II	1	40.77	685.7901 (2+)	685.7897	2-12	[*]***C***NTPTY***C***DLGK
III		40.18	685.7900 (2+)			
I		38.50				
II	2	38.68	429.7126 (2+)	429.7126	21-28	GYGFGMVK
III		38.07				
I		28.88	995.9266 (2+)			
II	3	28.92	995.9268 (2+)	995.9260	35-53	S***C***SGVEFSTSGHAYTDTGK
III		28.29	995.9266 (2+)			
I		38.07	811.4070 (2+)			
II	4	38.28	811.4072 (2+)	811.4060	62-74	YKV***C***NYGLTFTQK
III		37.64	811.4069 (2+)			
I		40.52	665.8274 (2+)			
II	5	40.53	665.8274 (2+)	665.8269	64-74	V***C***NYGLTFTQK
III		39.97	665.8275 (2+)			
I		51.53	954.4431 (2+)	954.4424		
II	6	51.32	636.6307 (3+)	636.6305	75-90	WNTDNTLGTEISWENK
III		51.04	954.4427 (2+)	954.4424		
I		58.16	677.3895 (3+)			
II	7	57.82	677.3898 (3+)	677.3893	91-109	LAEGLKLTLDTIFVPNTGK
III		57.68	677.3901 (3+)			
II	8	53.23	720.0878 (3+)	720.0876	91-110	LAEGLKLTLDTIFVPNTGKK
III		53.09				
I		55.61	709.8995 (2+)			
II	9	55.32	709.8992 (2+)	709.8984	97-109	LTLDTIFVPNTGK
III		55.10	709.8990 (2+)			
I		48.15	773.9463 (2+)	773.9456		
II	10	47.94	516.2999 (3+)	516.2999	97-110	LTLDTIFVPNTGKK
III		47.55	773.9459 (2+)	773.9456		
I		89.93	1512.6986 (3+)			
II	11	90.37	1512.6926 (3+)	1512.6995	121-161	D***C***FSLGSNVDIDFSGPTIYGWAVLAFE GWLAGYQMSFDTAK
III		89.98	1512.7026 (3+)			
I		32.20	490.5965 (3+)			
II	12	32.36	490.5967 (3+)	490.5967	162-174	SKLSQNNFALGYK
III		31.67	490.5965 (3+)			
I	13	38.93	627.8281 (2+)	627.8277	164-174	LSQNNFALGYK
II		39.06	627.8280 (2+)			

III		38.45	627.8281 (2+)			
I		40.16	845.7356 (3+)			
II	14	40.07	845.7352 (3+)	845.7352	175-197	AADFQLHTHVNDGTEFGGSIYQK
III		39.54	845.7358 (3+)			
I		49.73	924.4667 (2+)			
II	15	49.42	924.4669 (2+)	924.4662	202-218	IETSINLAWTAGSNNTR
III		49.04	924.4667 (2+)			
I		45.83	896.8406 (3+)			
II	16	45.74	896.8401 (3+)	896.8402	231-256	TSLSAKVNNASLIGLGYTQTLRPGVK
III		45.17	896.8403 (3+)			
I		51.32	860.4737 (2+)			
II	17	51.06	860.4738 (2+)	860.4736	237-252	VNNASLIGLGYTQTLR
III		50.77	860.4736 (2+)			
I		46.19	701.0648 (3+)			
II	18	46.12		701.0640	237-256	VNNASLIGLGYTQTLRPGVK
III		45.54	701.0649 (3+)			
II	19	17.86	422.7067 (2+)	422.7067	267-274	NFNAGGHK
III		17.81	422.7066 (2+)			
I		60.84	467.7475 (2+)	467.7477		
II	20	60.54	934.4890 (1+)	934.4880	275-283	VGLGFELEA
III		60.37	934.4879 (1+)			

*C: N-terminal acetylated; C: cysteine carboxyamidomethylated.

Table S8. Chymotryptic peptides found in the analysis of VDAC3 from the NSC34-SOD1WT cell line after DTT reduction, carboxyamidomethylation and in-solution digestion.
The retention time, experimentally measured and calculated monoisotopic m/z of the molecular ions, position in the sequence and peptide sequence of fragments present in the chymotryptic digest of reduced and carboxyamidomethylated VDAC3 are reported. These sequences were used to construct the sequence coverage shown in Figure 1.

Technical triplicate	Frag. n.	Rt (min)	Monoisotopic m/z		Position in the sequence	Peptide sequence
			Measured	Calculated		
I		33.83	820.8746 (2+)			
II	1	34.02	820.8748 (2+)	820.8745	2-15	*CNTPTYCDLGKAAK
III		34.51				
I		53.47	1001.4565 (2+)			
II	2	53.58	1001.4564 (2+)	1001.4562	2-18	*CNTPTYCDLGKAAKDVF
III		53.88	1001.4568 (2+)			
I	3	52.08	822.0481 (3+)	822.0476	2-22	*CNTPTYCDLGKAAKDVFNKGY
II		52.19	822.0478 (3+)			

I	4	34.76	612.3084 (2+)	612.3084	8-18	*C*DLGKAAKDVF
II		35.00	612.3082 (2+)			
III		35.39				
I	5	34.46	532.2932 (2+)	532.2933	9-18	DLGKAAKDVF
II		34.69	532.2977 (2+)			
I	6	54.45	490.2679 (2+)	490.2682	23-31	GFGMVKIDL
II		54.61	490.2677 (2+)			
III		54.82	490.2685 (2+)			
I	7	29.89	452.2701 (2+)	452.2708	25-32	GMVKIDLK
II		30.11	452.2702 (2+)			
III		30.46				
I	8	30.19	685.8351 (2+)	685.8352	30-41	DLKTKS*C*SGVEF
II		30.43	685.8350 (2+)			
III		30.85	685.8351 (2+)			
I	9	21.91	730.0110 (3+)	730.0105	42-62	STSGHAYTDTGKASGNLETKY
II		22.00	730.0105 (3+)			
III		22.59	730.0111 (3+)			
I	10	25.51	859.9153 (2+)	859.9158	47-62	AYTDTGKASGNLETKY
II		25.58	859.9160 (2+)			
III		26.05	859.9154 (2+)			
I	11	19.95	742.8658 (2+)	742.8656	49-62	TDTGKASGNLETKY
II		20.08	742.8655 (2+)			
III		20.42	742.8660 (2+)			
I	12	57.78	675.8130 (2+)	675.8128	76-87	NTDNTLGTEISW
II		57.92	675.8129 (2+)			
I	13	59.29	1103.0375 (2+)	1103.0377	76-95	NTDNTLGTEISWENKLAEGL
II		59.46	1103.0387 (2+)			
III		59.67	1103.0380 (2+)			
I	14	53.92	773.8918 (2+)	773.8915	82-95	GTEISWENKLAEGL
II		54.03	773.8921 (2+)			
III		54.30	773.8911 (+2			
I	15	42.19	855.4575 (1+)	855.4576	88-95	**E**NKLAEGL
II		42.41	855.4578 (1+)			
		30.62	873.4693 (1+)	873.4682		ENKLAEGL
I	16	47.71	516.3002 (3+)	516.3000	96-109	KLTLDTIFVPNTGK
II		47.82	516.2993 (3+)			
III		48.15	516.2994 (3+)			

I		40.36	558.9982 (3+)			
II	17	40.52	558.9979 (3+)	558.9984	96-110	KLTLDTIFVPNTGKK
III		41.03				
I		37.56	687.4091 (3+)			
II	18	37.69	687.4088 (3+)	687.4092	96-114	KLTLDTIFVPNTGKKSGKL
III		38.23	687.4093 (3+)			
I		37.74	478.6045 (3+)	478.6054		
II	19	37.86	717.4038 (2+)	717.4041	98-110	TLDTIFVPNTGKK
III		38.43	717.4041 (2+)			
I		34.82	607.0160 (3+)			
II	20	35.07	607.0162 (3+)	607.0162	98-114	TLDTIFVPNTGKKSGKL
III		35.49	607.0157 (3+)			
I		28.65	610.3375 (2+)			
II	21	28.83	610.3380 (2+)	610.3382	100-110	DTIFVPNTGKK
III		29.21	610.3386 (2+)			
I		27.83	535.6385 (3+)			
II	22	27.96	535.6392 (3+)	535.6390	100-114	DTIFVPNTGKKSGKL
III		28.33	535.6388 (3+)			
I		56.85	1066.5059 (1+)	1066.5057		
II	23	56.97	533.7565 (2+)	533.7568	124-133	SLGSNVDIDF
III		57.19	1066.5059 (1+)	1066.5057		
I		62.41	842.9078 (2+)			
II	24	62.54	842.9081 (2+)	842.9074	124-139	SLGSNVDIDFSGPTIY
III		62.70	842.9084 (2+)			
I		20.67	498.7718 (2+)			
II	25	20.75	498.7719 (2+)	498.7722	156-164	SFDTAKSKL
III		21.20	498.7718 (2+)			
I		30.97	793.8950 (2+)			
II	26	31.28	793.8945 (2+)	793.8946	156-169	SFDTAKSKLSQNNF
III		31.74	793.8950 (2+)			
I		37.11	690.8134 (2+)			
II		37.25	690.8132 (2+)	690.8131		**Q**LHTHVNDGTEF
III	27	37.84	690.8135 (2+)		179-190	
I		24.73	699.3268 (2+)	699.3261		
II		24.78	466.5527 (3+)	466.5535		QLHTHVNDGTEF
III		25.25	699.3264 (2+)	699.3261		
I	28	47.48	929.4248 (2+)	929.4243	179-195	**Q**LHTHVNDGTEFGGSIY

II		47.61	929.4247 (2+)			
III		47.96	929.4244 (2+)			
I		36.75	625.6276 (3+)	625.6277		QLHTHVNDGTEFGGSIY
II		36.86	625.6274 (3+)			
I	29	24.40	510.2255 (2+)	510.2256	182-190	THVNDGTEF
II		24.53	510.2252 (2+)			
III		25.01	510.2255 (2+)			
I	30	40.80	748.8367 (2+)	748.8368	182-195	THVNDGTEFGGSIY
II		40.99	748.8372 (2+)			
III		41.53				
I	31	23.74	550.2909 (2+)	550.2912	196-204	**Q**KVNERIET
I	32	35.20	650.3488 (2+)	650.3493	196-206	**Q**KVNERIETSI
II		35.47	650.3487 (2+)			
III		35.88	650.3484 (2+)			
I	33	44.71	763.9130 (2+)	763.9128	196-208	**Q**KVNERIETSINL
II		44.86	509.6104 (3+)	509.6111		
III		45.30	763.9131 (2+)	763.9128		
I		35.04	772.4263 (2+)	772.4261		QKVNERIETSINL
II		35.30	772.4268 (2+)			
III		35.69	772.4265 (2+)			
I	34	40.38	537.7932 (2+)	537.7936	200-208	ERIETSINL
II		40.54	537.7934 (2+)			
III		41.06	537.7935 (2+)			
I	35	49.00	924.4677 (2+)	924.4665	202-218	IETSINLAWTAGSNNTR
II		49.07	924.4675 (2+)			
I	36	27.06	496.7631 (2+)	496.7638	226-233	KLD**C**RTSL
II		27.56	496.7630 (2+)			
III		27.16	496.7632 (2+)			
I	37	42.18	593.8433 (2+)	593.8437	234-245	SAKVNNASLIGL
II		42.37	593.8436 (2+)			
III		42.85	593.8434 (2+)			
I	38	45.86	703.8870 (2+)	703.8861	234-247	SAKVNNASLIGLGY
II		46.02	703.8868 (2+)			
III		46.46	703.8867 (2+)			
I	39	50.50	589.8246 (2+)	589.8250	258-268	TLSALIDGKNF
II		50.54	589.8248 (2+)			
III		50.91	589.8250 (2+)			

I	40	37.44	482.7589 (2+)	482.7591	260-268	SALIDGKNF
II		37.14	482.7590 (2+)			
III		37.71	482.7584 (2+)			
I	41	26.84	528.7830 (2+)	528.7834	269-279	NAGGHKVGLGF
II		26.92	528.7832 (2+)			
III		27.35	528.7827(2+)			
I	42	40.16	500.2605 (3+)	500.2604	269-283	NAGGHKVGLGFELEA

C*: N-terminal acetylated; *C*: cysteine carboxyamidomethylated; **E/**Q**: pyroglutamic acid form.

Table S9. Retention time, experimentally measured and calculated monoisotopic m/z of the molecular ions, position in the sequence and peptide sequence of sulfur containing tryptic fragments found in the analysis of VDAC3 from the NSC34-SOD1WT cell line, reduced with DTT, carboxyamidomethylated and digested in-solution.

Technical triplicate	Frag. n.	Rt (min)	Monoisotopic m/z		Position in the sequence	Peptide sequence
			Measured	Calculated		
I	1	37.83	660.2665 (2+)	660.2663	2-12	**C**NTPTY*C*DLGK
II		37.99	660.2664 (2+)			
III		37.43	660.2666 (2+)			
I	2	33.47	991.4077 (2+)	991.4079	35-53	S**C**SGVEFSTSGHAYTDTGK
II		33.49	991.4097 (2+)			
III		32.93	991.4090 (2+)			
I	3	47.37	806.8885 (2+)	806.8877	62-74	YKV**C**NYGLTFTQK
II		44.21	806.8884 (2+)			
III		43.74	806.8885 (2+)			
I	4	49.01	661.3087 (2+)	661.3085	64-74	V**C**NYGLTFTQK
II		48.80	661.3077 (2+)			
III		48.37	661.3087 (2+)			
I	5	89.10	1518.0358 (3+)	1518.0315	121-161	D*C*FSLGSNVDIDFSGPTIYGW AVLAFEGWLAGYQ**M**SFDTA K
II		88.86	1518.0345 (3+)			
III		88.86	1518.0303 (3+)			
I	6	23.32	674.3146 (2+)	674.3166	150-161	LAGYQ**M**SFDTAK
II		23.20	674.3144 (2+)			
III		22.60	674.3145 (2+)			

C: cysteine carboxyamidomethylated; **C**: cysteine oxidized to sulfonic acid; **M**: methionine sulfoxide.

Table S10. Retention time, experimentally measured and calculated monoisotopic m/z of the molecular ions, position in the sequence and peptide sequence of sulfur containing chymotryptic fragments found in the analysis of VDAC3 from the NSC34-SOD1WT cell line reduced with DTT, carboxyamidomethylated and digested in-solution.

Technical triplicate	Frag. n.	Rt (min)	Monoisotopic m/z		Position in the sequence	Peptide sequence
			Measured	Calculated		
I	1	49.51	498.2652 (2+)	498.2657	23-31	GFG**M**VKIDL
II		49.59				

III		50.04				
I	2	23.02	460.2677 (2+)	460.2682	25-32	G**M**VKIDLK
II		23.14				
III		23.66	460.2678 (2+)			
I	3	34.95	492.2428 (2+)	492.2455	226-233	KLD**C**RTSL
II		30.94	492.2448 (2+)			
III		35.10	492.2432 (2+)			

C: cysteine oxidized to sulfonic acid; **M**: methionine sulfoxide.

Table S11. Ox/Red ratio of the absolute intensities of the molecular ions of sulfur containing tryptic peptides found in the analysis of VDAC3 from NSC34-SOD1WT cell line reduced with DTT, carboxyamidomethylated and digested in-solution.

Technical replicate	Peptide	Position in the sequence	Measured monoisotopic *m/z*	Absolute intensity	Ratio Ox/Red
I	S**C**SGVEFSTSGHAYTDTGK	35-53	991.4077 (2+)	$3.3 \cdot 10^4$	0.04
	S*C*SGVEFSTSGHAYTDTGK		995.9266 (2+)	$7.7 \cdot 10^5$	
II	S**C**SGVEFSTSGHAYTDTGK		991.4097 (2+)	$4.6 \cdot 10^4$	0.06
	S*C*SGVEFSTSGHAYTDTGK		995.9268 (2+)	$7.1 \cdot 10^5$	
III	S**C**SGVEFSTSGHAYTDTGK		991.4090 (2+)	$3.9 \cdot 10^4$	0.06
	S*C*SGVEFSTSGHAYTDTGK		995.9266 (2+)	$6.8 \cdot 10^5$	
I	YKV**C**NYGLTFTQK	62-74	806.8885 (2+)	$2.9 \cdot 10^5$	0.7
	YKV*C*NYGLTFTQK		811.4070 (2+)	$4.2 \cdot 10^5$	
II	YKV**C**NYGLTFTQK		806.8884 (2+)	$2.8 \cdot 10^5$	0.6
	YKV*C*NYGLTFTQK		811.4072 (2+)	$4.4 \cdot 10^5$	
III	YKV**C**NYGLTFTQK		806.8885 (2+)	$2.8 \cdot 10^5$	0.5
	YKV*C*NYGLTFTQK		811.4069 (2+)	$5.8 \cdot 10^5$	
I	D*C*FSLGSNVDIDFSGPTIYGWAVLAFE GWLAGYQ**M**SFDTAK	121-161	1518.0358 (3+)	$4.7 \cdot 10^4$	8.1
	D*C*FSLGSNVDIDFSGPTIYGWAVLAFE GWLAGYQMSFDTAK		1512.6986 (3+)	$5.8 \cdot 10^3$	
II	D*C*FSLGSNVDIDFSGPTIYGWAVLAFE GWLAGYQ**M**SFDTAK		1518.0345 (3+)	$6.1 \cdot 10^4$	5.5
	D*C*FSLGSNVDIDFSGPTIYGWAVLAFE GWLAGYQMSFDTAK		1512.6926 (3+)	$1.1 \cdot 10^4$	
III	D*C*FSLGSNVDIDFSGPTIYGWAVLAFE GWLAGYQ**M**SFDTAK		1518.0303 (3+)	$7.5 \cdot 10^4$	6.3
	D*C*FSLGSNVDIDFSGPTIYGWAVLAFE GWLAGYQMSFDTAK		1512.7026 (3+)	$1.2 \cdot 10^4$	

C: cysteine carboxyamidomethylated; **C**: cysteine oxidized to sulfonic acid; **M**: methionine sulfoxide.

Table S12. Ox/Red ratio of the absolute intensities of molecular ions of sulfur containing chymotryptic peptides found in the analysis of VDAC3 from NSC34-SOD1WT cell line reduced with DTT, carboxyamidomethylated and digested in-solution.

Technical replicate	Peptide	Position in the sequence	Measured monoisotopic m/z	Absolute intensity	Ratio Ox/Red
I	GFG**M**VKIDL	23-31	498.2652 (2+)	$1.8 \cdot 10^6$	12.9
	GFGMVKIDL		490.2679 (2+)	$1.4 \cdot 10^5$	
II	GFG**M**VKIDL		498.2651 (2+)	$1.9 \cdot 10^6$	10.6
	GFGMVKIDL		490.2677 (2+)	$1.8 \cdot 10^5$	
III	GFG**M**VKIDL		498.2652 (2+)	$1.4 \cdot 10^6$	12.7
	GFGMVKIDL		490.2685 (2+)	$1.1 \cdot 10^5$	
I	KLD**C**RTSL	226-233	492.2428 (2+)	$8.6 \cdot 10^4$	0.7

	KLD*C*RTSL		496.7631 (2+)	$1.3 \cdot 10^{5}$	
II	KLD<u>C</u>RTSL		492.2448 (2+)	$4.0 \cdot 10^{4}$	0.4
	KLD*C*RTSL		496.7630 (2+)	$9.0 \cdot 10^{4}$	
III	KLD<u>C</u>RTSL		492.2432 (2+)	$5.2 \cdot 10^{5}$	0.5
	KLD*C*RTSL		496.7632 (2+)	$1.0 \cdot 10^{6}$	

C: cysteine carboxyamidomethylated; <u>C</u>: cysteine oxidized to sulfonic acid; <u>M</u>: methionine sulfoxide.

Table S13. Retention time, experimentally measured and calculated monoisotopic m/z of the molecular ions, position in the sequence and peptide sequence of sulfur containing tryptic fragments found in the analysis of VDAC3 from the NSC34-SOD1G93A cell line reduced with DTT, carboxyamidomethylated and digested in-solution. Fragment 7 was used to construct the sequence coverage reported in Figure 1.

Technical triplicate	Frag. n.	Rt (min)	Monoisotopic m/z		Position in the sequence	Peptide sequence
			Measured	Calculated		
I	1	37.18	660.2664 (2+)	660.2663	2-12	<u>C</u>NTPTY*C*DLGK
II		37.09	660.2666 (2+)			
III		37.12	660.2665 (2+)			
I	2	30.13	437.7105 (2+)	437.7103	21-28	GYGFG<u>M</u>VK
II		29.93	437.7104 (2+)			
III		29.85	437.7106 (2+)			
I	3	32.82	991.4088 (2+)	991.4079	35-53	S<u>C</u>SGVEFSTSGHAYTDTGK
II		32.74	991.4086 (2+)			
III		32.75	991.4088 (2+)			
I	4	43.72	806.8881 (2+)	806.8877	62-74	YKV<u>C</u>NYGLTFTQK
II		43.62	806.8885 (2+)			
III		43.64	806.8882 (2+)			
I	5	48.35	661.3076 (2+)	661.3085	64-74	V<u>C</u>NYGLTFTQK
II		48.20	661.3084 (2+)			
III		48.21	661.3089 (2+)			
I	7	88.74	1518.0302 (3+)	1518.0315	121-161	D*C*FSLGSNVDIDFSGPTIYGWAVLAFEGWLAGYQ<u>M</u>SFDTAK
II		88.64	1518.0334 (3+)			
III		88.70	1518.0331 (3+)			
I	8	22.48	674.3145 (2+)	674.3166	150-161	LAGYQ<u>M</u>SFDTAK
II		22.41	674.3146 (2+)			
III		23.31	674.3147 (2+)			

C: cysteine carboxyamidomethylated; <u>C</u>: cysteine oxidized to sulfonic acid; <u>M</u>: methionine sulfoxide.

Table S14. Retention time, experimentally measured and calculated monoisotopic m/z of the molecular ions, position in the sequence and peptide sequence of sulfur containing chymotryptic fragments found in the analysis of VDAC3 from the NSC34 -SOD1G93A cell line, reduced with DTT, carboxyamidomethylated and digested in-solution. Fragment 1 was used to construct the sequence coverage reported in Figure 1.

Technical triplicate	Frag. n.	Rt (min)	Monoisotopic m/z		Position in the sequence	Peptide sequence
			Measured	Calculated		
I	1	49.64	498.2650 (2+)	498.2657	23-31	GFG**M**VKIDL
II		49.71	498.2650 (2+)			
III		49.81	498.2650 (2+)			
I	2	48.14	492.2433 (2+)	492.2455	226-233	KLD**C**RTSL
II		48.77	492.2433 (2+)			
III		48.59	492.2432 (2+)			

C: cysteine oxidized to sulfonic acid;**M**: methionine sulfoxide.

Table S15. Tryptic peptides found in the analysis of VDAC3 from the NSC34-SOD1G93A cell line after DTT reduction, carboxyamidomethylation and in-solution digestion.
The retention time, experimentally measured and calculated monoisotopic m/z of the molecular ions, position in the sequence and peptide sequence of fragments present in the tryptic digest analyzed in triplicate of reduced and carboxyamidomethylated VDAC3 are reported. All sequences were confirmed by MS/MS. These sequences were used to construct the sequence coverage shown in Figure 1.

Technical triplicate	Frag. n.	Rt (min)	Monoisotopic m/z		Position in the sequence	Peptide sequence
			Measured	Calculated		
I	1	40.12	685.7898 (2+)	685.7897	2-12	[*]*C*NTPTY*C*DLGK
II		39.96	685.7900 (2+)			
III						
I	2	28.34	995.9266 (2+)	995.9260	35-53	S*C*SGVEFSTSGHAYTDTGK
II		28.15	995.9269 (2+)			
III		28.07	995.9268 (2+)			
I	3	37.46	811.4070 (2+)	811.4060	62-74	YKV*C*NYGLTFTQK
II		37.31	811.4067 (2+)			
III		37.39	811.4069 (2+)			
I	4	39.89	665.8273 (2+)	665.8269	64-74	V*C*NYGLTFTQK
II		39.75	665.8274 (2+)			
III		39.78	665.8271 (2+)			
I	5	51.01	954.4428 (2+)	954.4424	75-90	WNTDNTLGTEISWENK
III		50.85	954.4433 (2+)			
I	6	57.73	677.3885 (3+)	677.3893	91-109	LAEGLKLTLDTIFVPNTGK
II		57.63	677.3898 (3+)			
I	7	53.09	720.0883 (3+)	720.0876	91-110	LAEGLKLTLDTIFVPNTGKK
I	8	55.13	709.8992 (2+)	709.8984	97-109	LTLDTIFVPNTGK
II		55.05	709.8993 (2+)			

III		55.11	709.8990 (2+)			
I	9	47.46	516.3003 (3+)	516.2999	97-110	LTLDTIFVPNTGKK
II		47.32	773.9465 (2+)	773.9456		
III		47.38	516.2999 (3+)	516.2999		
I	10	31.59	490.5967 (3+)	490.5967	162-174	SKLSQNNFALGYK
II		31.39	735.3917 (2+)	735.3910		
III		31.42	490.5965 (3+)	490.5967		
I	11	38.36	627.8282 (2+)	627.8277	164-174	LSQNNFALGYK
II		38.21	627.8282 (2+)			
III			627.8281 (2+)			
I	12	40.98	845.7349 (3+)	845.7352	175-197	AADFQLHTHVNDGTEFGGSIYQK
II		39.37	845.7360 (3+)			
III		39.39	845.7355 (3+)			
III	13	64.47	978.5167 (3+)	978.5125	198-224	VNERIETSINLAWTAGSNNTRFGIAAK
I	14	49.07	924.4669 (2+)	924.4662	202-218	IETSINLAWTAGSNNTR
II		48.93	924.4660 (2+)			
III		48.89	924.4663 (2+)			
I	15	45.16	896.8386 (3+)	896.8402	231-256	TSLSAKVNNASLIGLGYTQTLRPGVK
II		45.09	896.8406 (3+)			
I	16	50.74	860.4741 (2+)	860.4736	237-252	VNNASLIGLGYTQTLR
II		50.67	860.4735 (2+)			
III		50.55	860.4741 (2+)			
I	17	45.51	701.0646 (3+)	701.0640	237-256	VNNASLIGLGYTQTLRPGVK
II		45.45	701.0642 (3+)			
III		45.41	701.0663 (3+)			
II	18	44.00	619.3395 (3+)	619.3390	257-274	LTLSALIDGKNFNAGGHK
I	19	60.42	934.4878 (1+)	934.4880	275-283	VGLGFELEA
II		60.29	934.4890 (1+)			
III		60.30	934.4893 (1+)			

******C***: N-terminal acetylated; ***C***: cysteine carboxyamidomethylated.

Table S16. Chymotryptic peptides found in the analysis of VDAC3 from the NSC34-SOD1G93A cell line after DTT reduction, carboxyamidomethylation and in-solution digestion.
The retention time, experimentally measured and calculated monoisotopic m/z of the molecular ions, position in the sequence and peptide sequence of fragments present in the chymotryptic digest of reduced and carboxyamidomethylated VDAC3 are reported. These sequences were used to construct the sequence coverage reported in Figure 1.

Technical triplicate	Frag. n.	Rt (min)	Monoisotopic m/z		Position in the sequence	Peptide sequence
			Measured	Calculated		

I	1	33.97	820.8749 (2+)	820.8745	2-15	*CNTPTYCDLGKAAK
II		34.03	820.8748 (2+)			
I	2	53.55	1001.4567 (2+)	1001.4562	2-18	*CNTPTYCDLGKAAKDVF
II		53.68	1001.4569 (2+)			
III		53.64	1001.4568 (2+)			
I	3	52.09	822.0482 (3+)	822.0476	2-22	*CNTPTYCDLGKAAKDVFNKGY
I	4	34.84	612.3083 (2+)	612.3084	8-18	CDLGKAAKDVF
II		34.97	612.3062 (2+)			
III	5	34.70	532.2967 (2+)	532.2933	9-18	DLGKAAKDVF
I	6	30.32	457.5587 (3+)	457.5594	30-41	DLKTKSCSGVEF
III		30.57	685.8350 (2+)	685.8352		
I	7	21.74	730.0109 (3+)	730.0105	42-62	STSGHAYTDTGKASGNLETKY
II		22.16	730.0106 (3+)			
III		22.21	730.0101 (3+)			
I	8	25.45	573.6129 (3+)	573.6131	47-62	AYTDTGKASGNLETKY
II		25.81	573.6126 (3+)			
III		25.76	859.9165 (2+)	859.9158		
I	9	19.85	742.8658 (2+)	742.8656	49-62	TDTGKASGNLETKY
II		20.02	742.8658 (2+)			
III		20.16				
I	10	57.87	675.8130 (2+)	675.8128	76-87	NTDNTLGTEISW
III		57.93				
II	11	59.41	1103.0388 (2+)	1103.0377	76-95	NTDNTLGTEISWENKLAEGL
II	12	54.12	773.8921 (2+)	773.8915	82-95	GTEISWENKLAEGL
III		54.10	773.8917 (2+)			
I	13	30.46	873.4681 (1+)	873.4682	88-95	ENKLAEGL
III		30.76	873.4679 (1+)			
I	14	47.82	516.3004 (3+)	516.3000	96-109	KLTLDTIFVPNTGK
II		47.89	516.3000 (3+)			
I	15	40.32	558.9977 (3+)	558.9984	96-110	KLTLDTIFVPNTGKK
II		40.62				
III		40.80	558.9979 (3+)			
I	16	37.56	687.4091 (3+)	687.4092	96-114	KLTLDTIFVPNTGKKSGKL
III		38.00	687.4088 (3+)			
I	17	37.79	717.4039 (2+)	717.4041	98-110	TLDTIFVPNTGKK
II		37.93	478.6046 (3+)	478.6054		
III		38.15	717.4073 (2+)	717.4041		

I	18	35.01	607.0168 (3+)	607.0162	98-114	TLDTIFVPNTGKKSGKL
II		35.08	607.0167 (3+)			
III		35.21	607.0161 (3+)			
I	19	28.64	610.3385 (2+)	610.3382	100-110	DTIFVPNTGKK
II		29.00	610.3384 (2+)			
III		28.87	610.3376 (2+)			
I	20	27.78	535.6383 (3+)	535.6390	100-114	DTIFVPNTGKKSGKL
II		28.14	535.6386 (3+)			
III	21	50.54	423.2501 (3+)	423.2710	107-118	TGKKSGKLKASY
I	22	56.94	533.7565 (2+)	533.7568	124-133	SLGSNVDIDF
II		56.97				
III		57.00				
I	23	62.53	842.9078 (2+)	842.9074	124-139	SLGSNVDIDFSGPTIY
II		62.51	842.9080 (2+)			
III		62.48	842.9082 (2+)			
I	24	64.10	507.2402 (2+)	507.2405	145-153	AFEGWLAGY
I	25	20.51	498.7722 (2+)	498.7722	156-164	SFDTAKSKL
II		20.73	498.7721 (2+)			
III		20.90	498.7724 (2+)			
III	26	31.29	793.8947 (2+)	793.8946	156-169	SFDTAKSKLSQNNF
I	27	37.20	690.8129 (2+)	690.8131	179-190	**Q**LHTHVNDGTEF
II		37.36	690.8130 (2+)			
III		37.50	690.8135 (2+)			
I		24.62	699.3266 (2+)	699.3261		QLHTHVNDGTEF
II		25.09	466.5532 (3+)	466.5535		
III		24.99	699.3263 (2+)	699.3261		
I	28	47.59	929.4252 (2+)	929.4243	179-195	**Q**LHTHVNDGTEFGGSIY
II		47.66	929.4251 (2+)			
III		47.80	929.4250 (2+)			
I		36.84	625.6275 (3+)	625.6277		QLHTHVNDGTEFGGSIY
II		37.04	937.9395 (2+)	937.9376		
III		37.16	625.6274 (3+)	625.6277		
I	29	24.30	510.2256 (2+)	510.2256	182-190	THVNDGTEF
II		24.70	510.2249 (2+)			
III		24.74	510.2255 (2+)			
I	30	40.87	748.8365 (2+)	748.8368	182-195	THVNDGTEFGGSIY
II		41.04	748.8370 (2+)			

III		41.19	748.8365 (2+)			
II		24.07				
	31		550.2906 (2+)	550.2912	196-204	**Q**KVNERIET
III		24.12				
I		35.35	650.3465 (2+)			
II	32	35.43	650.3492 (2+)	650.3493	196-206	**Q**KVNERIETSI
III		35.55	650.3477 (2+)			
II		44.81	509.6107 (3+)	509.6111		**Q**KVNERIETSINL
III		46.31	763.9136 (2+)	763.9128		
I	33	35.17	772.4260 (2+)		196-208	
II		35.27	772.4263 (2+)	772.4261		QKVNERIETSINL
III		35.41	772.4266 (2+)			
II		55.01	892.4719 (2+)			
	34			892.4710	196-210	**Q**KVNERIETSINLAW
III		55.04	892.4713 (2+)			
II	35	48.84	612.7916 (2+)	612.7920	209-219	AWTAGSNNTRF
I	36	43.06	484.2331 (2+)	499.2338	211-219	TAGSNNTRF
I		49.64				
			496.7637 (2+)			
II	37	49.98		496.7638	226-233	KLD***C***RTSL
III		50.09	496.7635 (2+)			
I		42.19	593.8431 (2+)			
II	38	42.43	593.8430 (2+)	593.8437	234-245	SAKVNNASLIGL
III		42.54	593.8432 (2+)			
I		45.91	703.8868 (2+)			
II	39	46.02	703.8873 (2+)	703.8861	234-247	SAKVNNASLIGLGY
III		46.20	703.8864 (2+)			
I		50.61				
			589.8246 (2+)			
II	40	50.66		589.8250	258-268	TLSALIDGKNF
III		50.69	589.8250 (2+)			
I		37.54	482.7583 (2+)			
II	41	37.69	482.7589 (2+)	482.7591	260-268	SALIDGKNF
III		37.85	482.7550 (2+)			
I		26.76				
II	42	27.24	528.7828 (2+)	528.7834	269-279	NAGGHKVGLGF
III		27.12				
I		40.16	749.8859 (2+)			
				749.8866		
II	43	40.39	749.8867 (2+)		269-283	NAGGHKVGLGFELEA
III		40.59	500.2599 (3+)	500.2604		

****C***: N-terminal acetylated; ***C***: cysteine carboxyamidomethylated; **Q**: pyroglutamic acid form.

Table S17. Ox/Red ratio of the absolute intensities of molecular ions of sulfur containing tryptic peptides found in the analysis of VDAC3 from NSC34-SOD1G93A cell line reduced with DTT, carboxyamidomethylated and digested in-solution.

Technical replicate	Peptide	Position in the sequence	Measured monoisotopic *m/z*	Absolute intensity	Ratio Ox/Red
I	S**C**SGVEFSTSGHAYTDTGK	35-53	991.4088 (2+)	$8.9 \cdot 10^4$	0.06
	S*C*SGVEFSTSGHAYTDTGK		995.9266 (2+)	$1.6 \cdot 10^6$	
II	S**C**SGVEFSTSGHAYTDTGK		991.4086 (2+)	$7.0 \cdot 10^4$	0.07
	S*C*SGVEFSTSGHAYTDTGK		995.9269 (2+)	$1.0 \cdot 10^6$	
III	S**C**SGVEFSTSGHAYTDTGK		991.4088 (2+)	$6.9 \cdot 10^4$	0.06
	S*C*SGVEFSTSGHAYTDTGK		995.9268 (2+)	$1.1 \cdot 10^6$	
I	YKV**C**NYGLTFTQK	62-74	806.8881 (2+)	$2.0 \cdot 10^5$	0.8
	YKV*C*NYGLTFTQK		811.4081 (2+)	$2.5 \cdot 10^5$	
II	YKV**C**NYGLTFTQK		806.8885 (2+)	$1.7 \cdot 10^5$	0.6
	YKV*C*NYGLTFTQK		811.4067 (2+)	$2.7 \cdot 10^5$	
III	YKV**C**NYGLTFTQK		806.8882 (2+)	$1.8 \cdot 10^5$	0.6
	YKV*C*NYGLTFTQK		811.4069 (2+)	$2.9 \cdot 10^5$	

C: cysteine carboxyamidomethylated; **C**: cysteine oxidized to sulfonic acid.

Table S18. Ox/Red ratio of the absolute intensities of molecular ions of sulfur containing chymotryptic peptides found in the analysis of VDAC3 from NSC34-SOD1G93A cell line reduced with DTT, carboxyamidomethylated and digested in-solution.

Technical replicate	Peptide	Position in the sequence	Measured monoisotopic *m/z*	Absolute intensity	Ratio Ox/Red
I	KLD**C**RTSL	226-233	492.2433 (2+)	$6.2 \cdot 10^5$	0.2
	KLD*C*RTSL		496.7637 (2+)	$3.5 \cdot 10^6$	
II	KLD**C**RTSL		492.2433 (2+)	$4.0 \cdot 10^5$	0.3
	KLD*C*RTSL		496.7637 (2+)	$1.3 \cdot 10^6$	
III	KLD**C**RTSL		492.2432 (2+)	$2.2 \cdot 10^6$	0.5
	KLD*C*RTSL		496.7635 (2+)	$4.1 \cdot 10^6$	

C: cysteine carboxyamidomethylated; **C**: cysteine oxidized to sulfonic acid.

Table S19A. Retention time, experimentally measured and calculated monoisotopic m/z of the molecular ions, position in the sequence and peptide sequence of tryptic fragment containing succinated cysteines found in the analysis of VDAC3 from the NSC34 cell line, reduced with DTT, carboxyamidomethylated and digested in-solution.

Technical triplicate	Frag. n.	Rt (min)	Monoisotopic m/z		Position in the sequence	Peptide sequence
			Measured	Calculated		
I	1	42.88	560.9362 (3+)	560.9367	62-74	YKV**C**NYGLTFTQK
II		42.30	560.9362 (3+)			
III		42.25	560.9362 (3+)			

C: cysteine succinated.

Table S19B. Retention time, experimentally measured and calculated monoisotopic m/z of the molecular ions, position in the sequence and peptide sequence of tryptic fragment containing succinated cysteines found in the analysis of VDAC3 from the NSC34 SOD1WT cell line, reduced with DTT, carboxyamidomethylated and digested in-solution.

Technical triplicate	Frag. n.	Rt (min)	Monoisotopic m/z		Position in the sequence	Peptide sequence
			Measured	Calculated		
I	1	43.13	560.9362 (3+)	560.9367	62-74	YKV<u>C</u>NYGLTFTQK
II		42.89	560.9361 (3+)			
III		42.52	560.9363 (3+)			

<u>C</u>: cysteine succinated.

Table S19C. Retention time, experimentally measured and calculated monoisotopic m/z of the molecular ions, position in the sequence and peptide sequence of tryptic fragment containing succinated cysteines found in the analysis of VDAC3 from the NSC34-SOD1G93A cell line, reduced with DTT, carboxyamidomethylated and digested in-solution.

Technical triplicate	Frag. n.	Rt (min)	Monoisotopic m/z		Position in the sequence	Peptide sequence
			Measured	Calculated		
I	1	42.44	560.9361 (3+)	560.9367	62-74	YKV<u>C</u>NYGLTFTQK
II		42.24	560.9360 (3+)			
III		42.38	560.9362 (3+)			

<u>C</u>: cysteine succinated.

Table 20A. Ratio of the absolute intensities of molecular ions of tryptic peptides containing succinated and non-succinated cysteines found in the analysis of VDAC3 from NSC34 cell line reduced with DTT, carboxyamidomethylated and digested in-solution.

Technical replicate	Peptide	Position in the sequence	Measured monoisotopic *m/z*	Absolute intensity	Ratio Succinated/Red
I	YKV<u>C</u>NYGLTFTQK	62-74	560.9362 (3+)	$6.8 \cdot 10^4$	0.3
	YKV*C*NYGLTFTQK		811.4069 (2+)	$2.6 \cdot 10^5$	
II	YKV<u>C</u>NYGLTFTQK		560.9362 (3+)	$6.4 \cdot 10^4$	0.3
	YKV*C*NYGLTFTQK		811.4071(2+)	$2.6 \cdot 10^5$	
III	YKV<u>C</u>NYGLTFTQK		560.9362 (3+)	$7.9 \cdot 10^4$	0.3
	YKV*C*NYGLTFTQK		811.4070 (2+)	$2.5 \cdot 10^5$	

C: cysteine carboxyamidomethylated; <u>C</u>: cysteine succinated.

Table 20B. Ratio of the absolute intensities of the molecular ions of tryptic peptides containing succinated and non-succinated cysteines found in the analysis of VDAC3 from NSC34-SOD1WT cell line reduced with DTT, carboxyamidomethylated and digested in-solution.

Technical replicate	Peptide	Position in the sequence	Measured monoisotopic *m/z*	Absolute intensity	Ratio Succinated/Red
I	YKV<u>C</u>NYGLTFTQK	62-74	560.9362 (3+)	$1.9 \cdot 10^5$	0.5
	YKV*C*NYGLTFTQK		811.4070 (2+)	$4.2 \cdot 10^5$	
II	YKV<u>C</u>NYGLTFTQK		560.9361 (3+)	$1.8 \cdot 10^5$	0.4
	YKV*C*NYGLTFTQK		811.4072 (2+)	$4.4 \cdot 10^5$	
III	YKV<u>C</u>NYGLTFTQK		560.9363 (3+)	$1.9 \cdot 10^5$	0.3
	YKV*C*NYGLTFTQK		811.4069 (2+)	$5.8 \cdot 10^5$	

C: cysteine carboxyamidomethylated; <u>C</u>: cysteine succinated.

Table 20C. Ratio of the absolute intensities of molecular ions of tryptic peptides containing succinated and non-succinated cysteines found in the analysis of VDAC3 from NSC34-SOD1G93A cell line reduced with DTT, carboxyamidomethylated and digested in-solution.

Technical replicate	Peptide	Position in the sequence	Measured monoisotopic *m/z*	Absolute intensity	Ratio Succinated/Red
I	YKV**C**NYGLTFTQK	62-74	560.9361 (3+)	$2.9 \cdot 10^{5}$	1.2
	YKV*C*NYGLTFTQK		811.4081 (2+)	$2.5 \cdot 10^{5}$	
II	YKV**C**NYGLTFTQK		560.9360 (3+)	$2.7 \cdot 10^{5}$	1.0
	YKV*C*NYGLTFTQK		811.4067 (2+)	$2.7 \cdot 10^{5}$	
III	YKV**C**NYGLTFTQK		560.9362 (3+)	$2.8 \cdot 10^{5}$	1.0
	YKV*C*NYGLTFTQK		811.4069 (2+)	$2.9 \cdot 10^{5}$	

C: cysteine carboxyamidomethylated; **C**: cysteine succinated.

Table S21. Ratio of the absolute intensities of the molecular ions of tryptic peptides containing deamidated and non-deamidated asparagine found in the analysis of VDAC3 from NSC34-SOD1G93A cell line reduced with DTT, carboxyamidomethylated and digested in-solution.

Technical triplicate	Peptide	Position in the sequence	Measured monoisotopic m/z	Absolute intensity	Ratio Deam/Norm
I	W**N**TDNTLGTEISWENK	75-90	954.9356 (2+)	$5.3 \cdot 10^{4}$	0.003
	WNTDNTLGTEISWENK		954.4428 (2+)	$1.9 \cdot 10^{7}$	
III	W**N**TDNTLGTEISWENK		954.9379 (2+)	$2.9 \cdot 10^{4}$	0.002
	WNTDNTLGTEISWENK		954.4433 (2+)	$1.3 \cdot 10^{7}$	
I	LTLDTIFVP**N**TGK	97-109	710.3901 (2+)	$8.1 \cdot 10^{4}$	0.003
	LTLDTIFVPNTGK		709.8992 (2+)	$2.7 \cdot 10^{7}$	
II	LTLDTIFVP**N**TGK		710.3900 (2+)	$6.2 \cdot 10^{4}$	0.002
	LTLDTIFVPNTGK		709.8993 (2+)	$2.8 \cdot 10^{7}$	
I	LSQ**N**NFALGYK	164-174	628.3201 (2+)	$3.2 \cdot 10^{5}$	0.01
	LSQNNFALGYK		627.8282 (2+)	$2.4 \cdot 10^{7}$	
II	LSQ**N**NFALGYK		628.3201 (2+)	$2.9 \cdot 10^{5}$	0.01
	LSQNNFALGYK		627.8282 (2+)	$2.4 \cdot 10^{7}$	
III	LSQ**N**NFALGYK		628.3198 (2+)	$3.0 \cdot 10^{5}$	0.01
	LSQNNFALGYK		627.8281 (2+)	$2.5 \cdot 10^{7}$	
I	LSQN**N**FALGYK		628.3197 (2+)	$5.2 \cdot 10^{4}$	0.002
	LSQNNFALGYK		627.8282 (2+)	$2.4 \cdot 10^{7}$	
II	LSQN**N**FALGYK		628.3193 (2+)	$7.2 \cdot 10^{4}$	0.003
	LSQNNFALGYK		627.8282 (2+)	$2.4 \cdot 10^{7}$	
III	LSQN**N**FALGYK		628.3190 (2+)	$7.8 \cdot 10^{4}$	0.003
	LSQNNFALGYK		627.8281 (2+)	$2.5 \cdot 10^{7}$	
I	IETSINLAWTAGS**N**NTR	202-218	924.9586 (2+)	$8.1 \cdot 10^{5}$	0.1
	IETSINLAWTAGSNNTR		924.4669 (2+)	$1.4 \cdot 10^{7}$	
II	IETSINLAWTAGS**N**NTR		924.9585 (2+)	$6.9 \cdot 10^{5}$	0.1
	IETSINLAWTAGSNNTR		924.4660 (2+)	$1.2 \cdot 10^{7}$	
III	IETSINLAWTAGS**N**NTR		924.9590 (2+)	$6.3 \cdot 10^{5}$	0.1
	IETSINLAWTAGSNNTR		924.4663 (2+)	$1.3 \cdot 10^{7}$	
I	V**N**NASLIGLGYTQTLRPGVK	237-256	701.3922 (3+)	$3.4 \cdot 10^{5}$	0.01
	VNNASLIGLGYTQTLRPGVK		701.0646 (3+)	$5.1 \cdot 10^{7}$	
II	V**N**NASLIGLGYTQTLRPGVK		701.3923 (3+)	$4.6 \cdot 10^{5}$	0.01
	VNNASLIGLGYTQTLRPGVK		701.0642 (3+)	$4.7 \cdot 10^{7}$	
III	V**N**NASLIGLGYTQTLRPGVK		701.3920 (3+)	$3.5 \cdot 10^{5}$	0.01
	VNNASLIGLGYTQTLRPGVK		701.0663 (3+)	$2.7 \cdot 10^{7}$	
I	VN**N**ASLIGLGYTQTLRPGVK		701.3930 (3+)	$5.4 \cdot 10^{5}$	0.01

	VNNASLIGLGYTQTLRPGVK	701.0646 (3+)	$5.1 \cdot 10^7$	
II	VN**N**ASLIGLGYTQTLRPGVK	701.3920 (3+)	$3.6 \cdot 10^5$	0.01
	VNNASLIGLGYTQTLRPGVK	701.0642 (3+)	$4.7 \cdot 10^7$	
III	VN**N**ASLIGLGYTQTLRPGVK	701.3923 (3+)	$3.7 \cdot 10^5$	0.01
	VNNASLIGLGYTQTLRPGVK	701.0663 (3+)	$2.7 \cdot 10^7$	

N: asparagine deamidated.

Table S22. Retention time, experimentally measured and calculated monoisotopic m/z of the molecular ions, position in the sequence and peptide sequence of tryptic fragments containing asparagine residues in deaminated form found in the analysis of VDAC3 from the NSC34-SOD1G93A cell line, reduced with DTT, carboxyamidomethylated and digested in-solution. All sequences were confirmed by MS/MS.

Technical triplicate	Frag. n.	Rt (min)	Monoisotopic m/z		Position in the sequence	Peptide sequence
			Measured	Calculated		
I	1	52.75	954.9356 (2+)	954.9344	75-90	W**N**TDNTLGTEISWENK
III		52.44	954.9379 (2+)			
I	2	56.51	710.3901 (2+)	710.3904	97-109	LTLDTIFVP**N**TGK
II		56.43	710.3900 (2+)			
I	3	40.60	628.3201 (2+)	628.3197	164-174	LSQ**N**NFALGYK
II		40.48				
III			628.3198 (2+)			
I		41.78	628.3197 (2+)			LSQN**N**FALGYK
II		41.66	628.3193 (2+)			
III		41.70	628.3190 (2+)			
I	4	50.48	924.9586 (2+)	924.9582	202-218	IETSINLAWTAGS**N**NTR
II		50.40	924.9585 (2+)			
III		50.32	924.9590 (2+)			
I	5	47.72	701.3922 (3+)	701.3920	237-256	V**N**NASLIGLGYTQTLRPGVK
II		47.50	701.3923 (3+)			
III		48.31	701.3920 (3+)			
I		50.05	701.3930 (3+)			VN**N**ASLIGLGYTQTLRPGVK
II		48.37	701.3920 (3+)			
III		47.51	701.3923 (3+)			

N: asparagine deamidated.

Table S23. Retention time, experimentally measured and calculated monoisotopic m/z of the molecular ions, position in the sequence and peptide sequence of tryptic fragments containing asparagine residues in succinimide intermediate form found in the analysis of VDAC3 from the NSC34 cell line digest reduced with DTT, carboxyamidomethylated and digested in-solution.

Technical triplicate	Frag. n.	Rt (min)	Monoisotopic m/z		Position in the sequence	Peptide sequence
			Measured	Calculated		
I	1	41.00	619.3149 (2+)	619.3144	164-174	LSQ**N**NFALGYK
II		40.61	619.3157 (2+)			LSQ**NN**FALGYK
III		40.61	619.3137 (2+)			LSQN**N**FALGYK

N: succinimide intermediate; **NN**: N167 or N168 residue in the succinimide intermediate form.

Table S24. Retention time, experimentally measured and calculated monoisotopic m/z of the molecular ions, position in the sequence and peptide sequence of tryptic fragments containing asparagine residues in succinimide intermediate form found in the analysis of VDAC3 from the NSC34-SOD1WT cell line reduced with DTT, carboxyamidomethylated and digested in-solution. All sequences were confirmed by MS/MS.

Technical triplicate	Frag. n.	Rt (min)	Monoisotopic m/z		Position in the sequence	Peptide sequence
			Measured	Calculated		
I	1	41.42	619.3146(2+)	619.3144	164-174	LSQN**N**FALGYK
II		41.40	619.3145 (2+)			LSQ**N**NFALGYK
III		40.88	619.3149 (2+)			
I	2	55.37	1042.5801 (2+)	1042.5788	237-256	V**N**NASLIGLGYTQTLRPGVK
II		55.04	1042.5809 (2+)			
III		54.82	1042.5808 (2+)			

N: succinimide intermediate.

Table S25. Retention time, experimentally measured and calculated monoisotopic m/z of the molecular ions, position in the sequence and peptide sequence of tryptic fragments containing asparagine residues in succinimide intermediate form found in the analysis of VDAC3 from the NSC34-SOD1G93A cell line reduced with DTT, carboxyamidomethylated and digested in-solution. All sequences were confirmed by MS/MS.

Technical triplicate	Frag. n.	Rt (min)	Monoisotopic m/z		Position in the sequence	Peptide sequence
			Measured	Calculated		
I	1	40.78	619.3150 (2+)	619.3144	164-174	LSQN**N**FALGYK
II		40.69	619.3146 (2+)			LSQ**N**NFALGYK
III		40.65	619.3153 (2+)			

N: succinimide intermediate.

Table S26. Ratio of the absolute intensities of molecular ions of tryptic peptides containing succinimide intermediate found in the analysis of VDAC3 from NSC34 cell line reduced with DTT, carboxyamidomethylated and digested in-solution.

Technical replicate	Peptide	Position in the sequence	Measured monoisotopic m/z	Absolute intensity	Ratio Succinimide/Norm
I	LSQ**N**NFALGYK	164-174	619.3149 (2+)	$3.0 \cdot 10^4$	0.002
	LSQNNFALGYK		627.8282 (2+)	$1.5 \cdot 10^7$	
II	LSQ**NN**FALGYK		619.3157 (2+)	$4.7 \cdot 10^4$	0.003
	LSQNNFALGYK		627.8282 (2+)	$1.6 \cdot 10^7$	
III	LSQN**N**FALGYK		619.3137 (2+)	$2.3 \cdot 10^4$	0.002
	LSQNNFALGYK		627.8281 (2+)	$1.4 \cdot 10^7$	

N: succinimide intermediate; **NN**: N167 or N168 residue in the succinimide intermediate form.

Table S27. Ratio of the absolute intensities of the molecular ions of the succinimide intermediate containing tryptic peptides found in the analysis of VDAC3 from NSC34-SOD1WT cell line digest reduced with DTT,

carboxyamidomethylated and digested in-solution.

Technical replicate	Peptide	Position in the sequence	Measured monoisotopic m/z	Absolute intensity	Ratio Succinimide/Norm
I	LSQN**N**FALGYK	164-174	619.3146 (2+)	$1.5 \cdot 10^5$	0.004
	LSQNNFALGYK		627.8281 (2+)	$3.8 \cdot 10^7$	
II	LSQ**N**NFALGYK		619.3145 (2+)	$1.2 \cdot 10^5$	0.003
	LSQNNFALGYK		627.8282 (2+)	$3.8 \cdot 10^7$	
III	LSQ**N**NFALGYK		619.3149 (2+)	$1.2 \cdot 10^5$	0.003
	LSQNNFALGYK		627.8281 (2+)	$3.5 \cdot 10^7$	
I	V**N**NASLIGLGYTQTLRPGVK	237-256	1042.5801 (2+)	$1.4 \cdot 10^5$	0.003
	VNNASLIGLGYTQTLRPGVK		701.0648 (3+)	$5.1 \cdot 10^7$	
II	V**N**NASLIGLGYTQTLRPGVK		1042.5809 (2+)	$1.6 \cdot 10^5$	0.004
	VNNASLIGLGYTQTLRPGVK		701.0648 (3+)	$3.8 \cdot 10^7$	
III	V**N**NASLIGLGYTQTLRPGVK		1042.5808 (2+)	$1.1 \cdot 10^5$	0.002
	VNNASLIGLGYTQTLRPGVK		701.0649 (3+)	$5.3 \cdot 10^7$	

N: succinimide intermediate.

Table S28. Ratio of the absolute intensities of molecular ions of tryptic peptides containing succinimide intermediate found in the analysis of VDAC3 from NSC34-SOD1G93A cell line reduced with DTT, carboxyamidomethylated and digested in-solution.

Technical replicate	Peptide	Position in the sequence	Measured monoisotopic m/z	Absolute intensity	Ratio Succinimide/Norm
I	LSQN**N**FALGYK	164-174	619.3150 (2+)	$1.3 \cdot 10^5$	0.01
	LSQNNFALGYK		627.8282 (2+)	$2.4 \cdot 10^7$	
II	LSQ**N**NFALGYK		619.3146 (2+)	$1.4 \cdot 10^5$	0.01
	LSQNNFALGYK		627.8282 (2+)	$2.4 \cdot 10^7$	
III	LSQ**N**NFALGYK		619.3153 (2+)	$1.2 \cdot 10^5$	0.01
	LSQNNFALGYK		627.8281 (2+)	$2.5 \cdot 10^7$	

N: succinimide intermediate.

Table S29. Distribution of secondary structures as predicted by Ramachandran plots analysis. The table reports the percentage of favored, allowed and disallowed conformations assumed for each amino acidic residue in VDAC3 and VDAC3 N215D.

	VDAC3	VDAC3 N215D
Residues in the favored regions	267 (95.01%)	266 (94.66%)
Residues in allowed regions	12 (4,27%)	12 (4,27%)
Residues in disallowed regions	2 (0.72%)	3 (1,07%)
